# Station and train surface microbiomes of Mexico City’s metro (subway/underground)

**DOI:** 10.1038/s41598-020-65643-4

**Published:** 2020-05-29

**Authors:** Apolinar Misael Hernández, Daniela Vargas-Robles, Luis David Alcaraz, Mariana Peimbert

**Affiliations:** 1 Departamento de Ciencias Naturales. Unidad Cuajimalpa, Universidad Autónoma Metropolitana, Ciudad de México, México; 2 Centro Amazónico de Investigación y Control de Enfermedades Tropicales Servicio Autónomo CAICET, Puerto Ayacucho, Venezuela; 3 Departamento de Biología Celular, Facultad de Ciencias, Universidad Nacional Autónoma de México, Ciudad de México, México

**Keywords:** Genetic markers, Environmental microbiology, Biodiversity

## Abstract

The metro is one of the more representative urban transportation systems of Mexico City, and it transports approximately 4.5 million commuters every day. Large crowds promote the exchange of microbes between humans. In this study, we determined the bacterial diversity profile of the Mexico City metro by massive sequencing of the 16S rRNA gene. We identified a total of 50,174 operational taxonomic units (OTUs) and 1058 genera. The metro microbiome was dominated by the phylum Actinobacteria and by the genera *Cutibacterium* (15%) (*C. acnes* 13%), *Corynebacterium* (13%), *Streptococcus* (9%), and *Staphylococcus* (5%) (*S. epidermidis;* 4%), reflecting the microbe composition of healthy human skin. The metro likely microbial sources were skin, dust, saliva, and vaginal, with no fecal contribution detected. A total of 420 bacterial genera were universal to the twelve metro lines tested, and those genera contributed to 99.10% of the abundance. The annual 1.6 billion ridership makes this public transport a main hub for microbe-host-environment interactions. Finally, this study shows that the microbial composition of the Mexico City metro comes from a mixture of environmental and human sources and that commuters are exposed to healthy composition of the human microbiota.

## Introduction

Public transport systems provide the ideal environment for the transmission of microorganisms, as they carry a multitude of passengers, and their microbiomes, daily. The metro can assemble an extensive repository of beneficial bacteria, such as commensals and symbionts, or harmful bacteria, becoming a vehicle for the transmission of infectious diseases. The Mexico City metropolitan area has a population of 21.3 million people, which makes it the largest city in the Western Hemisphere. Mexico City’s metro has more than 4 million users every day, totaling 1,647,475,013 users annually (https://metro.cdmx.gob.mx/) and is the second busiest metro of the American continent, after New York City’s, and the ninth busiest in the world^1^. It has been in use since 1969 and today has 12 lines and 196 stations; it travels through a network of 226.5 km and operates 365 days of the year, covering a radius of around 10 km around the urban sprawl of Mexico City. The various lines were individually constructed at different points in history, which has led to differences in the infrastructure and the wagon type between lines; although all lines have pneumatic wheels, some also have iron wheels. Most of the lines go through different sections, including underground, street level, and elevated above ground level, and are ventilated by extractors, fans, or open windows. The size of passenger influx differs by line and by the station, with a higher influx at terminal stations and transfer stations. Terminal stations connect the inner city to suburban areas, where most of the population lives. During this mass movement, passengers breathe the same air and touch the same surfaces, promoting a large-scale interchange of human and environmental microbiota. Several factors differentiate the metros of each city. In Mexico City, there are three rush hours per day: the commute to and from work and another peak at noon that seems to correspond to the schools’ schedule. The number of trips/person/days is 2.37, similar to London and Seville (2.31 and 2.33, respectively), but much lower than the New York and Paris averages (3.79 and 3.70, respectively)^2^.

Previous studies have indicated that a significant proportion of metro surface bacteria come from the skin of passengers, so the lifestyle of these passengers is also important. Among the chemical and physical factors directly influencing the microbiome, the most obvious are temperature and humidity. However, there are many other important factors, such as differences in the ventilation systems, whether or not the trains are pneumatic, and pollution. Therefore, studies from other networks can be used for reference, but the local diversity will only be understood by directly studying the metros of Mexico City. Multiple studies have characterized the metro microbiomes of various densely populated areas, such as New York City, Boston, Hong Kong, and Barcelona^3–8^. Mexico City is one of the most densely populated areas in the world (6,000 people/km^2^ in Mexico City; 9,800 people/km^2^ in the Greater Mexico City area). However, Mexico City’s metro microbiome has been scarcely explored. Only one previous study has characterized the colony-forming units (CFU; N = 175 isolates) in the air of the metro transport system identifying three fungal genera and concluding that most bacteria were Gram-positive bacilli^9^.

It has been estimated that humans can emit 10^6^ particles per hour^10,11^. As a rough estimation, the 4 × 10^6^ Mexico City metro users per day, with an average commuting time of 2.3 hours^9^, release a total of 9.2 × 10^12^ particles in the metro every day. Additionally, the metro architecture favors the presence of multiple aerosol particles from other sources so that the metro can be thought of as a vast shared pool for the horizontal exchange of microbes from human-derived particles, soil, and plant debris. In previous work, the built environment was shown to be biologically different when inhabited by people, compared to empty spaces, and even the “cloud” of microbiomes can be used to distinguish the microbial signatures of individuals four hours after they have departed^11,12^. In this study, we revealed the bacterial diversity profile of the Mexico City metro with next generation sequencing of the 16S rRNA gene. We took 47 samples from stations and trains, covering all 12 metro lines of Mexico City. This work is the first culture-independent investigation of a metro microbiome in Latin America.

## Results

**Figure 1:**
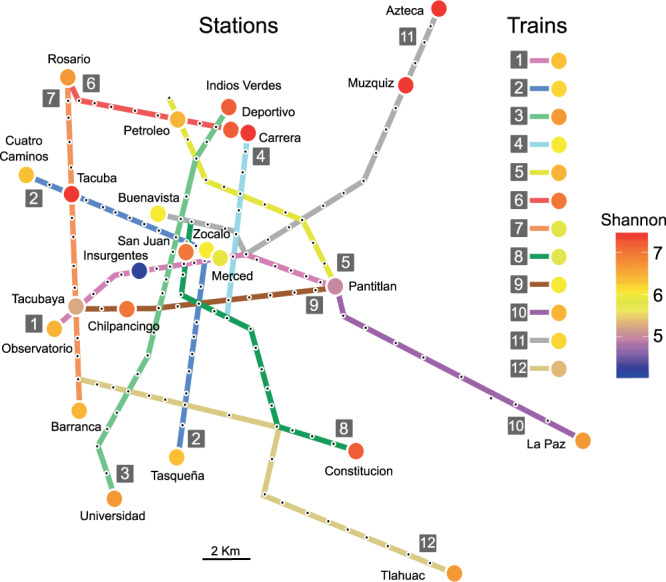
Sampling diversity and location. The metro network, with each black dot representing a station and colored circles showing the sampling locations. The circles are colored according to the Shannon diversity index. Train diversity is also shown in colored circles.

We studied the twelve Mexico City metro lines and analyzed 47 samples for this study (Fig. 1). The samples were taken from station turnstiles (N = 24) and train handrails (N = 23). We sequenced a total of 16.6 × 10^6^ V3-V4 16S rRNA gene reads (4.98 Gb in total). The reads were merged into 5,788,162 sequences (avg = 460 bp) with a mean of 123,152.38 ± 28,371.93 sequences/sample. This work represents the largest published record of Mexico City’s metro microbiological diversity. The sequences were clustered into operational taxonomic units (OTUs; 97% identity 16S rRNA gene), recovering 50,174 OTUs (Supplementary Table S1). The OTUs were then homology-matched and annotated with reference databases being summarized as 1,058 distinct bacteria genera. Additionally, we detected 22,673 amplicon sequence variants (ASVs) and 1,252 bacterial genera (Supplementary Fig. 1; Table S2).

**Figure 2:**
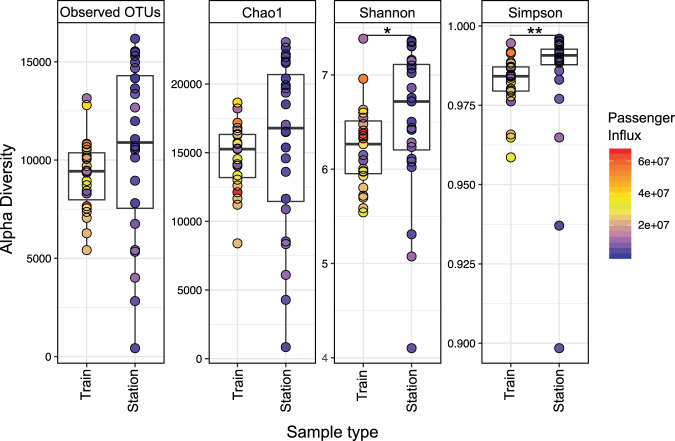
OTUs richness and diversity of Mexico City’s metro. The surface microbiome diversity on the station entrance and the metro train. A total of 50,174 OTUs were observed, across 47 samples (*p < 0.05, **p < 0.01, Kruskal-Wallis test).

Differences between observed and expected OTUs (Chao1) are less than 30% (Fig. 2). Moreover, we are confident of a comprehensive sampling of the metro’s diversity because differences between the observed genera and Chao1 diversity index are less than 10% (Fig. S3). The Simpson diversity index were slightly higher for the stations when compared to trains (Supplementary Table S3). The most diverse station was Carrera, with a Shannon diversity index of H′ = 7.36, followed by Azteca (H′ = 7.35), Muzquiz (H′ = 7.31), Tacuba (H′ = 7.30), and Indios Verdes (H′ = 7.16); the lowest diversity was found on metro Line 1 (Insurgentes station; H′ = 4.10) (Fig. 1). The train with the highest diversity was found on Line 6 (H′ = 7.38), followed by Line 3 (H′ = 6.96), and line 5 (H′ = 6.63). The lowest diversity train was found on Line 8 (H′ = 5.54). The average Shannon diversity (H′_(average)_ = 6.52) from the stations (turnstiles) was higher than the average train (handrails) diversity (H′_(average)_ = 6.24) (Fig. 2; Supplementary Table S3). We also calculated alpha diversity using amplicon sequence variants (ASVs), and observed lower diversity than the one calculated with OTUs (ASV H′_(average)_ = 4.93 ± 0.74; Supplementary Fig. S1). The lower H′ diversity for ASVs was expected, considering the ASV counts (22,673) compared to the OTUs found (50,174).

We looked for relationships between microbial diversity and a multitude of factors like metro line numbers, station connectivity (regular, transfer, and terminal stations), station surroundings (bus terminals, work-related buildings, schools, hospitals), architectural structure (above ground, below ground stations), train wheels (metallic and pneumatic), train design (individual wagons or connected), temperature, relative humidity, geographical zones, and passenger influx (Fig. S2). However, there were only statistically significant differences (*p* ≤ *0.01*) between the surfaces of stations and trains. Stations showed significant larger OTU diversity than trains and had higher diversity dispersions (Fig. 2).

**Figure 3:**
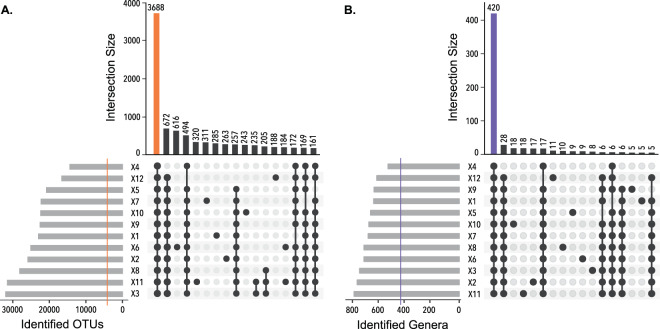
Shared taxa between metro lines. All the train and station samples are merged into its source metro line. The histogram shows the number of shared elements for each intersection sets. The plots are ordered from higher to lower cardinal numbers. (**A**) Shared OTUs: of the 50,174 OTUs, 3,688 were found in all lines. (**B**) Shared genera: of 1,058 genera, 420 were found in all lines. The 420 genera represented 99.10% of the entire dataset. The gray bars show the number of taxa identified for each subway line. Sets smaller than 150 elements for OTUs, and sets smaller than 5 elements for genera, were excluded from the diagrams.

Mexico City’s metro is highly diverse at the OTU level (50,174 OTUs). By merging the bacterial genera diversity by the metro line, we found that 420/1,058 genera were ubiquitous throughout the metro system (Fig. 3). Moreover, these 420 genera, representing 3,688 OTUs, constituted 99.10% of the whole dataset, expressed in sequence abundance. The other 638/1,058 genera contributed to just 0.9% of the overall richness. We are confident this work has comprehensively described the Mexico City metro bacterial genera. It was surprising to find the presence of 420 genera in all of our samples (Fig. 3).

**Figure 4:**
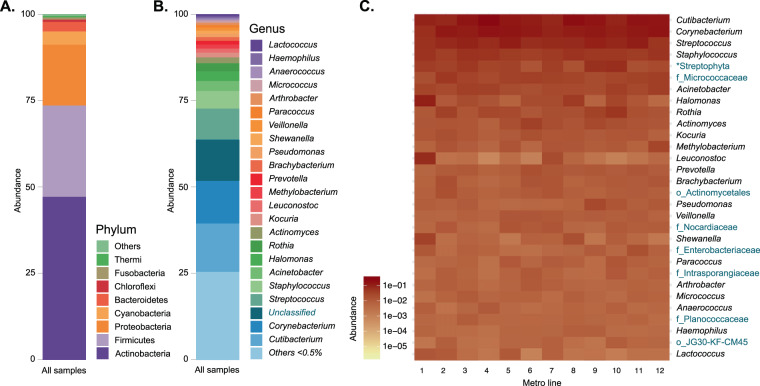
Phylogenetic profile of Mexico City’s metro surfaces. (**A**) Phylum abundance in all samples: Actinobacteria was the most abundant phylum followed by Firmicutes and Proteobacteria. (**B**) Genus abundance: *Cutibacterium* was the most abundant genus. (**C**) Heatmap of the 30 most abundant taxa, and their abundance in each sampled metro line. The unknown genera were classified into the higher known taxonomic rank, shown in different text colors. The asterisk indicates plants.

The main phyla detected were Actinobacteria, Firmicutes, Proteobacteria, Cyanobacteria, Bacteroidetes, Chloroflexi, Fusobacteria, and Thermi (Fig. 4A), no differences were found using ASVs or OTUs nor Silva or Green genes databases in phyla assignments. There were multiple genera with low abundance (1,034 genera <0.5%) in our study, and the most abundant known genera were *Cutibacterium* (15%), *Corynebacterium* (13%), *Streptococcus* (9%), and *Staphylococcus* (5%) (Fig. 4B). Interestingly, some of the most abundant genera were unclassified, but we were able to assign them to higher taxonomic hierarchies (29.43%). The ubiquity and prevalence of the core taxa are exemplified in a heatmap showing the 30 most abundant genera in the system, which are also dominant in each metro line (Fig. 4C); the complete 420 genera heatmap and table are also available (Fig. S4, To understand the interaction between people and metro surfaces Supplementary Table S4). The heatmap also shows the higher taxonomic level classifications for unknown genera. It points out some candidates for examination, such as members of the families Micrococcaceae, Nocardiaceae, Enterobacteriaceae, Intrasporangiaceae, and Planococcaceae, along with members from the order Actinomycetales and the class Thermomicrobia (Fig. 4C). The fifth most abundant phylotype was classified as chloroplast (Streptophyta). Archaea were also marginally detected with a relative abundance of 0.001%, and the most represented archaeal genus was *Methanobrevibacter* (0.0004%).

**Figure 5:**
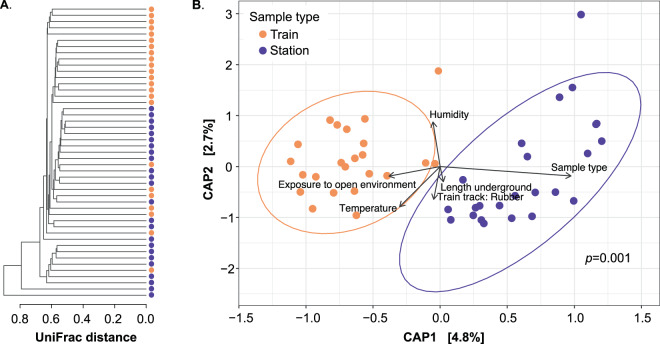
Metro bacterial communities grouped into the sample types collected in this study: for train and station. (**A**) Community distances dendrogram using unweighted UniFrac distances. (**B**) Constrained analysis of principal coordinates (CAP) ordinations based on unweighted UniFrac distances for turnstiles and handrails at the OTU level. Significantly segregated (*p* = 0.001, Adonis) ellipses denote the 95% confidence interval of the points distribution by the station and train samples sample type.

The metro as a whole has a consistent microbiome regardless of location. However, there are subtle differences at the OTU level that suggest the possibility of using taxa to discriminate between the local environments. The higher diversity and OTU richness of station turnstiles, compared to train handrails, suggested the existence of two microbial communities. To confirm the environment clustering, we performed *β*-diversity analysis by unweighted UniFrac dendrogram analysis. We found two main clades, suggesting train handrails and station turnstiles communities (Fig. 5A). To test the separation of the two community clusters we performed a constrained analysis of principal coordinates (CAP) ordinations based on unweighted (Fig. 5B**)** and weighted (Supplemetary Fig. S5) UniFrac distances at the OTU level. The analysis shows two discrete clusters that segregated trains from stations (unweighted and weighted, *p* ≤ 0.001, PERMANOVA). Sample dispersion remained similar between groups (unweighted, *p* = 0.182, weighted, *p* = 0.242, BETADISPER). However, a total of only 7.5% variance was explained in both CAP axes (Fig. 5B). The CAP-tested variables showed that exposure to open environments and the stations samples were associated with CAP1 dispersion, while humidity and temperature were the best options for explaining CAP2 dispersion (Fig. 5B).

After CAP analysis confirmed that microbial composition can be separeted into trains and station communities, we did a differential abundance analysis using DESeq2^13^ to evaluate significant enrichments (*p* < 1 × 10^−5^) and its fold changes between communities. The most of the enriched taxa belong to the Actinobacteria, 110/113 train OTUs, and 102/131 station taxa (Supplementary Table. S6).The significantly enriched OTUs in trains, compared to stations were *Corynebacterium* (N = 65), *Cutibacterium* (N = 38), and single OTUs belonging to *Actinomyces, Brevibacterium, Gardnerella, Rothia, Streptomyces*, and *Veillonella*. While stations enriched phylotypes were *Kocuria* (N = 14), *Blastococcus* (N = 6), *Geodermatophilus* (N = 5), 38 other known genera (N = 1), and unclassified to the genus level (N = 57 OTUs), but classified mainly to Actinobacteria (N = 45), Firmicutes (N = 4), Proteobacteria (N = 3), Chloroflexi (N = 2), Cyanobacteria (N = 2), and Gemmatimonadetes (N = 1) (Supplementary Table S6).

To understand the interaction between people and metro surfaces, we observed and documented the behavior of 3098 passengers (see Methods). We observed that 78% (570/730) of people touched the station turnstiles directly with their hands, while the rest touched it with their clothes or carry-on items. The observation of train handrail use was complicated, since some people touch many handrails, some hold tight to just one, and others do not touch anything. However, we observed that the rate of direct contact with one of the station turnstile bars is significantly higher (median, 122 people/hour) than with one of the train handrails (median 22.9 people/hour, *p* = 0.001). Observations were made during rush hour (Fig. S6).

**Figure 6:**
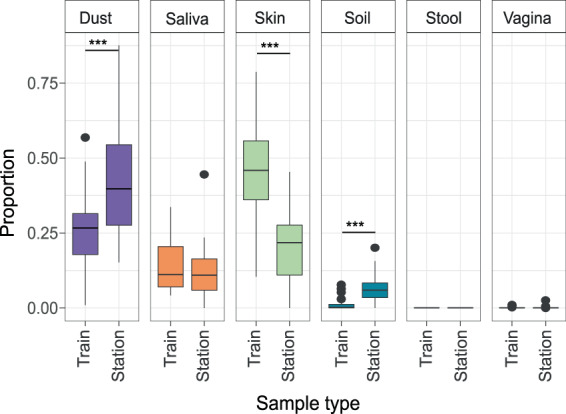
Source tracking comparison between stations and train microbiota. A source tracker algorithm was used to identify microbes to the genus level, using dust, soil, saliva, skin, feces, and vaginal samples as potential sources (***p < 0.001, Student’s t-test). Dots in the figure represent outliers from the boxplot quartiles.

We performed a source-tracking analysis to determine the possible environmental sources of the metro microbial communities. The tracked microbial environments were dust, skin, saliva, vaginal, stool, and soil. A mean of 34% of our data was matched by the source tracker algorithm^14^ (see Methods) to microbiota from dust, while skin (32%), saliva (13%), soil (4%), and vaginal matches were less prevalent (0.1%). No stool profiles were detected. The trains showed a higher level of skin microbiota than station (*p* = 1e-06, Student’s t-test), while stations had a higher level of dust and soil than trains (*p* < 0.001). Saliva and vaginal matches to the source-tracking database were not distributed differentially between trains and stations. We did not detect significant differences in environmental sources between samples exposed to the external environment and completely subterraneous samples (Fig. 6; Supplementary Fig. S7).

## Discussion

The richness of the microbiome of Mexico City’s metro was 50,174 OTUs (16S rRNA gene V3–V4), which is comparable to reports from other cities. The following comparisons were made by a raw contrast of the main findings for other subways. For example, in a study of the Hong Kong subway system, using similar methodological procedures (16S rRNA gene V4), there were reported to be a total of 55,703 OTUs^6^. However, Shannon’s diversity index (H′) is two orders of magnitude larger in Mexico City’s metro (H′_(average)_ = 6.38 ± 0.661) than the reported for Hong Kong subway (H′_(average)_ = 4.13 ± 0.307^15^) and the Barcelona subway airborne bacteria (H′ ~ 1.5)^8^. Additionally, the inverse Simpson index of diversity (D_(average)_ = 0.98 ± 0.004) is also higher than the Boston subway surface microbial diversity (D ~ 0.75, as estimated from a previous study^4^). The Shannon diversity is higher than that of some human microbiome systems^15^. Overall bacterial diversity found in Mexico City’s metro is similar to that of subways in other countries, with its source microbes derived mainly from dust, human skin, and oral taxa (Fig. 6)^3,5–7^. However, each city metro has different dominant taxa. *Pseudomonas stutzeri*, *Stenotrophomonas maltophila, Enterobacter cloacae*, and *Acinetobacter* are the most abundant species in New York City^5^. *Methylobacterium i*s the dominant genus in Barcelona’s subway^8^. Neither *Pseudomonas* nor *Methylobacterium*, prevalent in the NYC and Barcelona subways, are among the most abundant genera found in this study (Fig. 4)^5,8^.

In Mexico’s metro microbiome, the four most abundant genera were *Cutibacterium* (15%) (*C. acnes* 13%), *Corynebacterium* spp. (13%), *Streptococcus* spp. (9%), and *Staphylococcus* spp. (5%) (*S. epidermidis*, 4%). *C. acnes* (Basonym: *Propionibacterium acnes*^16^) on trains reached a similar relative abundance to that reported for passengers’ hands after a metro trip (~29%)^17^. Similar taxa were observed in Mexico City’s metro and Hong Kong subway with a dominance of skin associated microbiota like *Cutibacterium, Corynebacterium*, *Staphylococcus*, and *Streptococcus*^6^. Despite the similarities with Boston and Hong Kong’s bacterial diversity, Mexico City metro has larger 𝛼-diversity. It has been noted that microbial composition is driven by geography at the OTU level, when comparing the Hong Kong and United States OTU phylogenetic diversity of built environments^6^. Remarkably, three cultural contrasting subway systems share the same dominant bacterial genera, with similar ridership between Mexico City (world ninth) and Hong Kong (10th)^1^ and the lower ridership Boston subway (82th). The MetaSUB is a consortium working to develop sample collection, DNA/RNA isolation, and sequencing standard operating procedures to study global microbial diversity in mass transport systems^18^. This work began before the MetaSUB protocols were published. However, the sampling, DNA isolation, PCR programs, sequencing technologies, and data processing are in line with the MetaSUB requirements. Further meta-analysis work could test the geographical and host effects in the transport system associated bacteria.

Multiple factors affect the metro microbiome composition, such as the number of users, microenvironmental and climatic conditions, architecture, and even rush hours, and some studies have identified antibiotic resistance genes as morning or afternoon signatures^17^. In this work, no architectonic designs (subterranean, street level, or elevated trains, or rubber or ferrous wheels) affected microbial diversity or composition. The high frequency of surface interaction by users probably promotes the rapid exchange of microbes, preventing them from establishing and growing. Environmental variables (temperature and humidity) appear to be similar among sampled sites; additionally, the type of environment outside the train station (bus terminals, work-related buildings, schools, hospitals) did not modify the microbial diversity, suggesting the samples were independent of the station context.

The higher diversity we found on the stations compared to trains may be explicable in a number of ways. The station turnstiles are exposed to the outside, which may explain the greater diversity and also why soil bacteria are more highly represented in these samples. Another relevant dissimilarity is the number of people who touch the bars, 122 people/hour for turnstiles, and 23 people/hour for handrails (Supplementary Fig. S6). Differences in usage may also explain the differences in microbial diversity and composition. Train handrails are usually touched with the hands, while station turnstiles are often touched by clothes, which favors the arrival of dust-associated taxa. Finally, the difference in material surfaces may also shape diversity, *e.g*., turnstiles are aluminum, while handrails are made of stainless steel. The effects in surface usage and material have been previously associated with changes in microbial diversity and composition^4^. The Actinobacteria phylum is highly represented (18,727 OTUs; 37.31%) in Mexico City’s metro, and we were able to identify 160 known genera and 3,959 undescribed genus-level OTUs in this phylum. The leading genera were found to be *Cutibacterium* and *Corynebacterium*. Interestingly, *Cutibacterium* has been isolated from human skin and sometimes described as primary human pathogen^19,20^. We found a total of 2,341 OTUs classified as *Cutibacterium*, with 1,358 OTUs best matching *C. acnes*, and 105 OTUs best matching *C. granulosum*, as well as 878 unknown *Cutibacterium* species. *Cutibacterium* has been described in association with acne lesions and in surgical infections but has also been observed in healthy skin^16,21^. Further work of different species and strains of *Cutibacterium* is needed to understand its role in healthy skin and built environments.

*Corynebacterium* has been described in soil, freshwater, marine, and host-associated environments^22^. *Corynebacterium* had more OTUs (4,238), albeit in lower abundances than *Cutibacterium*. The vast abundance of *Cutibacterium* and *Corynebacterium* could be due to the prolific host microenvironments of the sebaceous glands, with their secreted fatty acids and a low pH (~5) and O_2_ concentration^23^. The biotechnological potential of detected Actinobacteria in the Mexico City’s metro is astounding. Actinobacteria are also known to be active members of micro-communities and act as symbionts to eukaryotes^21^. We detected 160 known genera of Actinobacteria, follow up projects could use this diversity to mine for new antibiotics and natural products, explore the potential for biomass decomposition from dust (which is mainly human skin debris in the metro), and explore their possible role in bioenergy production. It is also interesting that, together, Actinobacteria and Firmicutes represented almost 75% of the total described diversity (Fig. 4A). Firmicutes are represented by 154 known genera, with a high abundance of *Streptococcus, Staphylococcus, Leuconostoc, Lactobacillus, Veillonella, Anaerococcus*, and *Bacillus*.

The fifth most abundant biological group in Mexico City’s metro was classified as chloroplasts (1,129 OTUs identified as the land plant Streptophyta). We were also able to identify unicellular algae (Stramenopiles and Chlorophyta). A second search matching chloroplasts OTUs to RDP’s seqmatch^24^ allowed a detailed classification of the plants (Supplementary Table S5). Plant identification allows us to detect plants rooted in the Mexican cultural identity, like maize and its wild relative teosintes (*Zea*), common bean (*Phaseolus vulgaris*), some edible plants like cucumber (*Cucumis sativus*), papaya (*Carica papaya*), lettuce (*Lactuca sativa*), sunflower (*Helianthus annuus*), and pea (*Pisum sativum*). In addition, we found recreative plants like coffee (*Coffea arabica*) and tobacco (*Nicotiana tabacum*) in our dataset. Other species included the most notorious introduced plant of Mexico City’s roadsides, *Eucalyptus globulus*, as well as firs (*Abies*), abele (*Populus alba*), pine (*Pinus*), red cedar (*Juniperus virginiana*), ornamental plants like cycads (*Cycas taitungensis*), and all common plants growing in the city or its surroundings. Some of these plant genera have local centers of diversity or are regularly consumed by Mexico’s population. It was unexpected to find a plant signal in our study. We employed a harsh cell lysis method based on physical bead-beating and detergent disruption of membranes (MoBio’s PowerSoil) to extract DNA from the surfaces and the deposited bioaerosols. Possible plant sources found in this study could be derived from plant debris, pollen, and plant-derived products. Studying macro species with metagenomic environmental DNA has allowed us to track rare species of vertebrates^25^, invading plant species^26^, and lotic communities^27^. Further work on metagenomic DNA degradation in the metro will be useful to determine the spatiotemporal dynamics of microbe transmission and DNA resilience of other taxa, such as the plant species we detected in the metro.

The ubiquity of the 420 genera found in all the samples is probably indicating their success within the species pool of this built habitat. Mexico City’s metro is a place where feedback between millions of hosts and the shared environment is shaping the host and environmental microbial communities’ dispersal, transmission, and feedback. Mexico City’s bacterial species pool has the metro as its playground. The species pool is defined as the microbes residing independently of their hosts, capable of colonizing and interacting with its abiotic environment, other inhabitants, and hosts^28^. The microbial species pool had also been defined in the microbiome context as microbes residing outside the host at sometimes in their life cycle, with the possibility to colonize its hosts or the interaction with other microbes with colonizing capabilities^28^. Species pools can be a part of any given community, and their success is measured by the similarity of species within a community, their frequency, dispersal abilities, and dormancy capabilities^29^. So, if similar organisms dominate a community structure, they have succeeded surpassing abiotic and biotic filtering to be part of that community^29^. The functional and environmental selection for a habitat has been studied extensively by botanists. We can think about a forest and “forest species” let us say that pine species dominate the forest, we can think about the pine species pool success in a hypothetical forest. The human oral microbiome is reflecting the success of the Streptococci species pool. Streptococci are responsible for the health and disease of their hosts within the oral cavity^30,31^. More dramatically, the success, based on the species similarity and richness of the Lactobacillus pool in the human vaginal microbiome, is undeniable^32^. The species pool is involved in the dispersal of microbes between their hosts and the environment, and it is shaped by the host and environmental feedback (i.e., immune system responses, host population density, continuous dispersal) that can actively shape the microbial community^28^.

Further work could explore the diversity of Mexico City’s microbial pool and its impact on human health. The species pool could be used to introduce microbiota to humans during the first years of life, to train and mature the immune system a process that has been observed with newborn babies who have extensive variation in their skin microbiomes during the first years that subsequently stabilized^33^. It has been stated that humans living in developed countries are reducing their microbiota diversity, compared to non-urban and non-industrialized lifestyles^34^. So, studying the species pool transmission, resilience, and feedback with the millions of ridership in urban transportation systems will help to understand human-microbe interactions, especially in the urban context. Previous reports, have shown that dispersal and transmission of microbes of human individuals sharing their home share more microbes, even between genetic twins^35^. This is consistent in other animal microbiomes like fishes^36^. Host dissemination and acquisition of microbes could be beneficial to the establishment of symbiotic interactions at the community, microbiome-level where the interactions sustain the mutualistic bacteria and prevent potential pathogens and cheaters, at least in a theoretical model^37^. The Mexico City’s metro is a venue for microbiome transmission city-wide scale, with an annual ridership of 1.6 billion users, it will be a fertile ground for future testing of metacommunity theory into microbiomes of built environments.

## Materials and Methods

### Sampling

Station turnstiles from the entrance and vertical handrails inside the train were sampled with sterile cotton swabs between April 27^th^ to May 5th, 2016. Each swab was used to sample 3 different bars, covering approximately 300 cm^2^ (100 cm^2^ from each bar). Before sampling, swabs were moistened with transport media (Tris 20 mM, EDTA 10 mM pH 7.5). Samples were kept on ice during transport and stored at −80 °C until DNA extraction. Turnstiles from 24 stations were sampled. A total of 24 samples were collected from train handrails, two trains for each metro line. The library of a sample did not meet the quality criteria. Therefore only 23 train samples are reported. Sampling permits were granted by the metro “User Support Manager” (*Gerencia de Atención al Usuario, del Sistema de Transporte Colectivo*).

### Metadata

Temperature and relative humidity were recorded for the sampled site. Information about the train building architectural design (underground, elevated or street level), train wheel type (rubber tires or steel wheels), and station information, such as climate zone, type of station (way, transfer, or terminal station), train environmental exposure level (open or subterraneous space) was recorded. Additionally, the environmental context of the exterior of the metro stations (office buildings, bus station, market, park, or plaza) was also collected. Passenger influx statistical data were obtained from the metro office reports, “Sistema de Transporte Colectivo: Cifras de Operación” (https://www.metro.cdmx.gob.mx/operacion/cifras-de-operacion) (Supplementary Table S6).

### Passenger behavior observations

Frequency of contact with train handrail and station turnstile bars was determined by counting the number of users touching a specific bar within an hour (a total of 3002 people among 8 turnstiles and 96 people among 9 handrails). Additionally, a total of 52 turnstiles in six different stations were observed for 10 minutes. Also, a total of 32 handrail bars were observed during the whole train route (22 to 39 minutes) in 8 different lines. The total number of touches per turnstile bar was averaged and were extrapolated to touches per hour.

### Amplicon generation and next generation sequencing

Metagenomic DNA was obtained using the MoBio PowerSoil Kit (MoBio Laboratories, Solana Beach, CA), with the small modifications suggested by MoBio for low biomass samples. Briefly, swabs were vortexed in a 1.5 mL tube with 300 μL of transportation buffer (Tris 20 mM, EDTA 10 mM pH 7.5). Then, 125 μL of the sample, 30 μL of C1 solution, and 50 μL phenol: chloroform 1:1 were mixed in the bead tube. The following steps followed the manufacturer’s instructions. We used PCR primers for the V3–V4 region of the 16S rRNA gene^38^, following MiSeq™ Illumina® protocol with 5′ overhangs for sequencing library preparation (341 F and 805 R) with an expected amplicon size of 464 bp^38^. Each PCR was done in triplicate with a reaction mixture of 25 μL including 0.2 μL Phusion DNA polymerase™, 5 μL Buffer 5×, 2.5 μL dNTP (3 mM), 1 μL forward primer (5 pmol/μL), 1 μL reverse primer (5 pmol/μL), 2 μL DNA, and 13.3 μL water. The PCR reaction was initiated at 98 °C, 30 s, followed by 35 cycles of 92 °C for 10 s, 53 °C for 30 s, and 72 °C for 40 s, the reaction finished at 72 °C for 5 min. Sterile swabs were used for negative controls. PCR amplicons were purified using the High Pure PCR Product Purification Kit of ROCHE™ (Roche Diagnostics Gmb, Mannheim, Germany). Sequencing was performed using a MiSeq™ Illumina® (2 × 300 bp) platform at the Biotechnology Institute of the Universidad Nacional Autónoma de México (UNAM).

### Sequence processing

We used a previously reported protocol for 16S rRNA gene amplicons analysis^39^, which is detailed at GitHub (https://genomica-fciencias-unam.github.io/SOP/). Briefly, pair end reads were merged, using a minimum of 470 bp, a 15 bp of minimum overlapping, and with a quality threshold of 0.95 using PANDASEQ^40^. Sequence clustering into OTUs was performed at 97% identity with cd-hit-est^41^. Cd-hit-est is an included OTU picking software in the popular QIIME scripts^42^, which we have previously tested as a reasonable option when picking *de novo* OTUs^39^. Representative sequences and OTU tables were built with QIIME (v 1.9)^42^. The taxonomy assignment was completed with parallel BLAST+^43^ against the Greengenes database^44^. Best matching species are suggested with sequence identity >90% and e-values <1e-100, and then the best match confirmation against NCBI’s type strains 16S ribosomal RNA database. Plant-derived sequences were initially classified with the Greengenes database^43^, and then the sequences matching plants or mithocondria were extracted and then classified using the ribosomal database project^24^. Additionally, amplicon sequence variants (ASVs) analysis was done using DADA2 (v 1.10.1)^45^ to denoise, merge reads, remove chimeras, and assign taxonomy using Silva database (v 138)^46^ (https://genomica-fciencias-unam.github.io/metro/).

### Diversity metrics and statistical analysis

Diversity calculations and statistical inferences were performed with the R packages “phyloseq2” and “ggplot2”^47,48^ or using R default functions^49^ for both OTUs and ASVs. Beta diversity ordination analyses were evaluated with Canonical Analysis of Principal Coordinates (CAP) for weighted and unweighted UniFrac distances at the OTU level, exploring the correlation with the following variables: sample_type + temperature_C + Humidity + length_underground + train_track + exposed_open_environment. PERMANOVA were performed per variable using the ANOVA function with 999 permutations. Sample dispersion was also evaluated with *betadisper* R function^50^. The source tracker algorithm^14^ was used at the genus level, using 16S rRNA sequences (V4 region) from mattress dust, soil, saliva, skin, and fecal samples^51^ and vaginal samples^52^ as reference sources. Differential enrichment of taxa analyses were performed with R’s package “DESeq2”^13^.

## Supplementary information

Supplementary material.

## Data Availability

The data have been deposited in the DDBJ/EMBL/GenBank BioProject database under the accession ID PRJNA554099. Nucleotide sequences are available from DDBJ/EMBL/GenBank databases under the accession numbers SAMN12255545 - SAMN12255591.

## References

[1] International Association of Public Transport. *World Metro Figures 2018*, http://www.uitp.org/world-metro-figures-2018 (2018).

[2] Casado Izquierdo JM (2014). Patrones horarios de la movilidad cotidiana en la Zona Metropolitana del Valle de México. Scr. Nova. Rev. Electrónica Geogr. y Ciencias Soc..

[3] Walker AR, Grimes TL, Datta S, Datta S (2018). Unraveling bacterial fingerprints of city subways from microbiome 16S gene profiles. Biol. Direct.

[4] Hsu T (2016). Urban transit system microbial communities differ by surface type and interaction with humans and the environment. mSystems.

[5] Afshinnekoo E (2015). Geospatial resolution of human and bacterial diversity with city-scale metagenomics. Cell Syst..

[6] Leung MHY, Wilkins D, Li EKT, Kong FKF, Lee PKH (2014). Indoor-air microbiome in an urban subway network: Diversity and dynamics. Appl. Environ. Microbiol..

[7] Robertson CE (2013). Culture-independent analysis of aerosol microbiology in a metropolitan subway system. Appl. Environ. Microbiol..

[8] Triadó-Margarit X (2017). Bioaerosols in the Barcelona subway system. Indoor Air.

[9] Hernández-Castillo O (2014). Aerobiological study in the Mexico City subway system. Aerobiologia (Bologna)..

[10] Meadow JF (2015). Humans differ in their personal microbial cloud. PeerJ.

[11] Bhangar S (2016). Chamber bioaerosol study: human emissions of size-resolved fluorescent biological aerosol particles. Indoor Air.

[12] You R, Cui W, Chen C, Zhao B (2013). Measuring the short-term emission rates of particles in the ‘personal cloud’ with different clothes and activity intensities in a sealed chamber. Aerosol Air Qual. Res..

[13] Love MI, Huber W, Anders S (2014). Moderated estimation of fold change and dispersion for RNA-seq data with DESeq2. Genome Biol..

[14] Knights D (2011). Bayesian community-wide culture-independent microbial source tracking. Nat. Methods.

[15] Li K, Bihan M, Yooseph S, Methé BA (2012). Analyses of the microbial diversity across the human microbiome. PLoS One.

[16] Scholz CFP, Kilian M (2016). The natural history of cutaneous propionibacteria, and reclassification of selected species within the genus *Propionibacterium* to the proposed novel genera *Acidipropionibacterium* gen. nov., Cutibacterium gen. nov. and *Pseudopropionibacterium* gen. nov. Int. J. Syst. Evol. Microbiol..

[17] Kang K (2018). The environmental exposures and inner- and intercity traffic flows of the metro System may contribute to the skin microbiome and resistome. Cell Rep..

[18] The MetaSUB International Consortium. (2016). The metagenomics and metadesign of the subways and urban biomes. Microbiome.

[19] Renz N, Mudrovcic S, Perka C, Trampuz A (2018). Orthopedic implant-associated infections caused by Cutibacterium spp.–A remaining diagnostic challenge. PloS one.

[20] Dréno B (2018). *Cutibacterium acnes* (*Propionibacterium acnes*) and acne vulgaris: a brief look at the latest updates. Journal of the European Academy of Dermatology and Venereology.

[21] Piwowarek K, Lipińska E, Hać-Szymańczuk E, Kieliszek M, Ścibisz I (2018). *Propionibacterium* spp.—source of propionic acid, vitamin B12, and other metabolites important for the industry. Appl. Microbiol. Biotechnol..

[22] Lewin GR (2016). Evolution and ecology of Actinobacteria and their bioenergy applications. Annu. Rev. Microbiol..

[23] Grice EA, Segre JA (2011). The skin microbiome. Nat. Rev. Microbiol..

[24] Cole JR (2014). Ribosomal Database Project: Data and tools for high throughput rRNA analysis. Nucleic Acids Res..

[25] Franklin TW (2019). Using environmental DNA methods to improve winter surveys for rare carnivores: DNA from snow and improved noninvasive techniques. Biol. Conserv..

[26] Gantz CA, Renshaw MA, Erickson D, Lodge DM, Egan SP (2018). Environmental DNA detection of aquatic invasive plants in lab mesocosm and natural field conditions. Biol. Invasions.

[27] Seymour M (2018). Acidity promotes degradation of multi-species environmental DNA in lotic mesocosms. Commun. Biol..

[28] Miller ET, Svanbäck R, Bohannan BJM (2018). Microbiomes as metacommunities: Understanding host-associated microbes through metacommunity ecology. Trends Ecol. Evol..

[29] Zobel M, van der Maarel E, Dupré C (1998). Species pool: the concept, its determination and significance for community restoration. Appl. Veg. Sci..

[30] Belda-Ferre P (2011). The oral metagenome in health and disease. ISME J..

[31] Alcaraz LD (2012). Identifying a healthy oral microbiome through metagenomics. Clin. Microbiol. Infect..

[32] Gajer P (2012). Temporal dynamics of the human vaginal microbiota. Sci. Transl. Med..

[33] Palmer C, Bik EM, DiGiulio DB, Relman DA, Brown PO (2007). Development of the human infant intestinal microbiota. PLoS Biol..

[34] Clemente JC (2015). The microbiome of uncontacted Amerindians. Sci. Adv..

[35] Rothschild D (2018). Environment dominates over host genetics in shaping human gut microbiota. Nature.

[36] Burns AR (2017). Interhost dispersal alters microbiome assembly and can overwhelm host innate immunity in an experimental zebrafish model. Proc. Natl. Acad. Sci. USA.

[37] Pillai P, Gouhier TC, Vollmer SV (2014). The cryptic role of biodiversity in the emergence of host-microbial mutualisms. Ecol. Lett..

[38] Illumina. 16s metagenomic sequencing library preparation. https://support.illumina.com/downloads/16s_metagenomic_sequencing_library_preparation.html (2013).

[39] Alcaraz LD (2018). Marchantia liverworts as a proxy to plants’ basal microbiomes. Sci. Rep..

[40] Masella AP, Bartram AK, Truszkowski JM, Brown DG, Neufeld JD (2012). PANDAseq: paired-end assembler for illumina sequences. BMC Bioinformatics.

[41] Huang Y, Niu B, Gao Y, Fu L, Li W (2010). CD-HIT Suite: a web server for clustering and comparing biological sequences. Bioinformatics.

[42] Caporaso JG (2010). QIIME allows analysis of high-throughput community sequencing data. Nat. Methods.

[43] Camacho C (2009). BLAST+: Architecture and applications. BMC Bioinformatics.

[44] DeSantis TZ (2006). Greengenes, a chimera-checked 16S rRNA gene database and workbench compatible with ARB. Appl. Environ. Microbiol..

[45] Callahan BJ, Sankaran K, Fukuyama JA, McMurdie PJ, Holmes SP (2016). Bioconductor Workflow for Microbiome Data Analysis: from raw reads to community analyses. F1000Research.

[46] Yilmaz P (2013). The SILVA and “All-species Living Tree Project (LTP)” taxonomic frameworks. Nucleic Acids Research.

[47] McMurdie PJ, Holmes S (2013). phyloseq: An R package for reproducible interactive analysis and graphics of microbiome census data. PLoS One.

[48] Wickham, H. Ggplot2: elegant graphics for data analysis. (Springer (2016).

[49] R Core Team. R: A language and environment for statistical computing. (2013).

[50] Oksanen J (2007). The vegan package. Community ecology package..

[51] Marotz C (2017). DNA extraction for streamlined metagenomics of diverse environmental samples. Biotechniques.

[52] Dominguez-Bello MG (2016). Partial restoration of the microbiota of cesarean-born infants via vaginal microbial transfer. Nat. Med..

