# Supplementary material for: Station and train surface microbiomes of Mexico City’s metro (subway/underground)
Source: Sci Rep. 2020 May 29;10:8798. doi: 10.1038/s41598-020-65643-4 (PMC7260218; doi:10.1038/s41598-020-65643-4)
Supplement: Supplementary file 1 — Supplementary material. [file 41598_2020_65643_MOESM1_ESM.pdf]

Station and train surface microbiomes of Mexico City's metro (subway/underground)

Apolinar Misael Hernández[a], Daniela Vargas-Robles[a,b], Luis D. Alcaraz[c], Mariana Peimbert[a*]

[a] Departamento de Ciencias Naturales. Unidad Cuajimalpa. Universidad Autónoma Metropolitana. Av. Vasco de Quiroga 4871, Col. Santa Fe Cuajimalpa, 05348, Cd. Mx., México.

[b] Centro Amazónico de Investigación y Control de Enfermedades Tropicales Servicio Autónomo CAICET, Ministerio del Poder Popular para la Salud, Puerto Ayacucho, Estado Amazonas, Venezuela.

[c] Departamento de Biología Celular, Facultad de Ciencias, Universidad Nacional Autónoma de México, Ciudad Universitaria, UNAM, 04510, Cd. Mx., México.

*Correspondence author: marianapeimbert@gmail.com +52 55581 46500, 3806

Supplementary Material

Fig. S1. ASV diversity and phylogenetic profile of Mexico City's metro

Fig. S2. Association between temperature, humidity, or passenger influx with Shannon diversity in handrails and turnstiles.

Fig. S3. Genera richness and diversity of Mexico City's metro.

Fig. S4. Heatmap of the 420 shared genera and their abundance in each sampled metro line.

Fig. S5. Beta diversity based on weighted UniFrac distances for turnstiles and handrails at the OTU level.

Fig. S6. Handrails and turnstiles users touch rate at peak hour.

Fig. S7. Source tracking of stations and train microbiota.

Table S1. Sequencing effort, pair-end merged sequences, and OTUs summary.

Table S2. ASVs summary.

Table S3. Alpha diversity (OTUs and Genera).

Table S4. Core genera found in the Mexico City's metro.

Table S5. Plant species identified using the mitochondrial ribosomal genes.

Table S6. OTUs with differential abundances between trains and stations.

Table S7. Samples metadata.

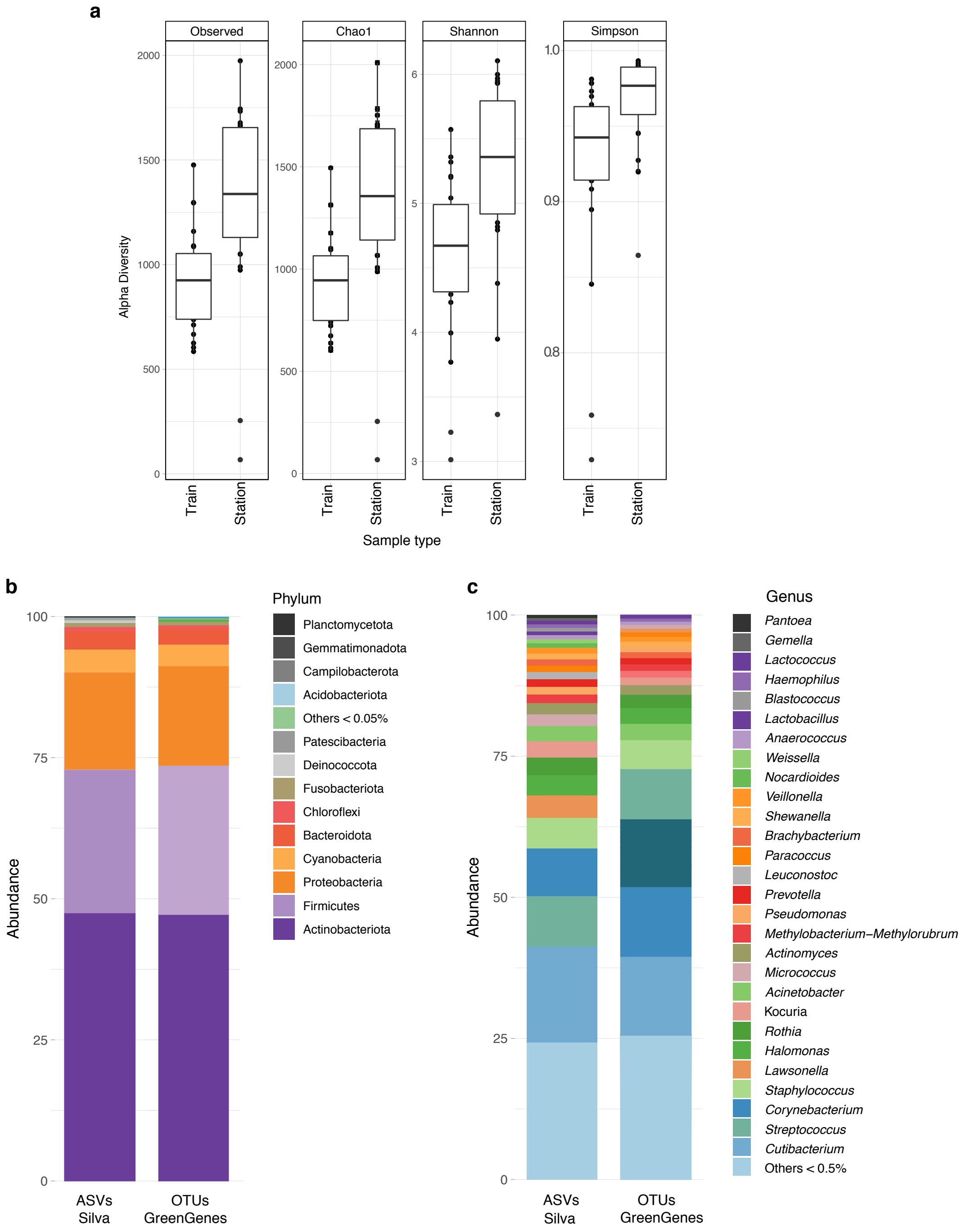

Fig. S1. **ASVs diversity and phylogenetic profile of Mexico City's metro. A.** Alpha diversity metrics for ASVs. A total of 22,673 ASVs were observed. ASV alpha diversity metrics were lower than those for OTUs. **B.** Phylum abundance in all samples. It was similar between ASVs and OTUs. Actinobacteria was the most abundant phylum followed by Firmicutes and Proteobacteria. **C.** Genus abundance; the most abundant genera were the same for ASV and OTUs.

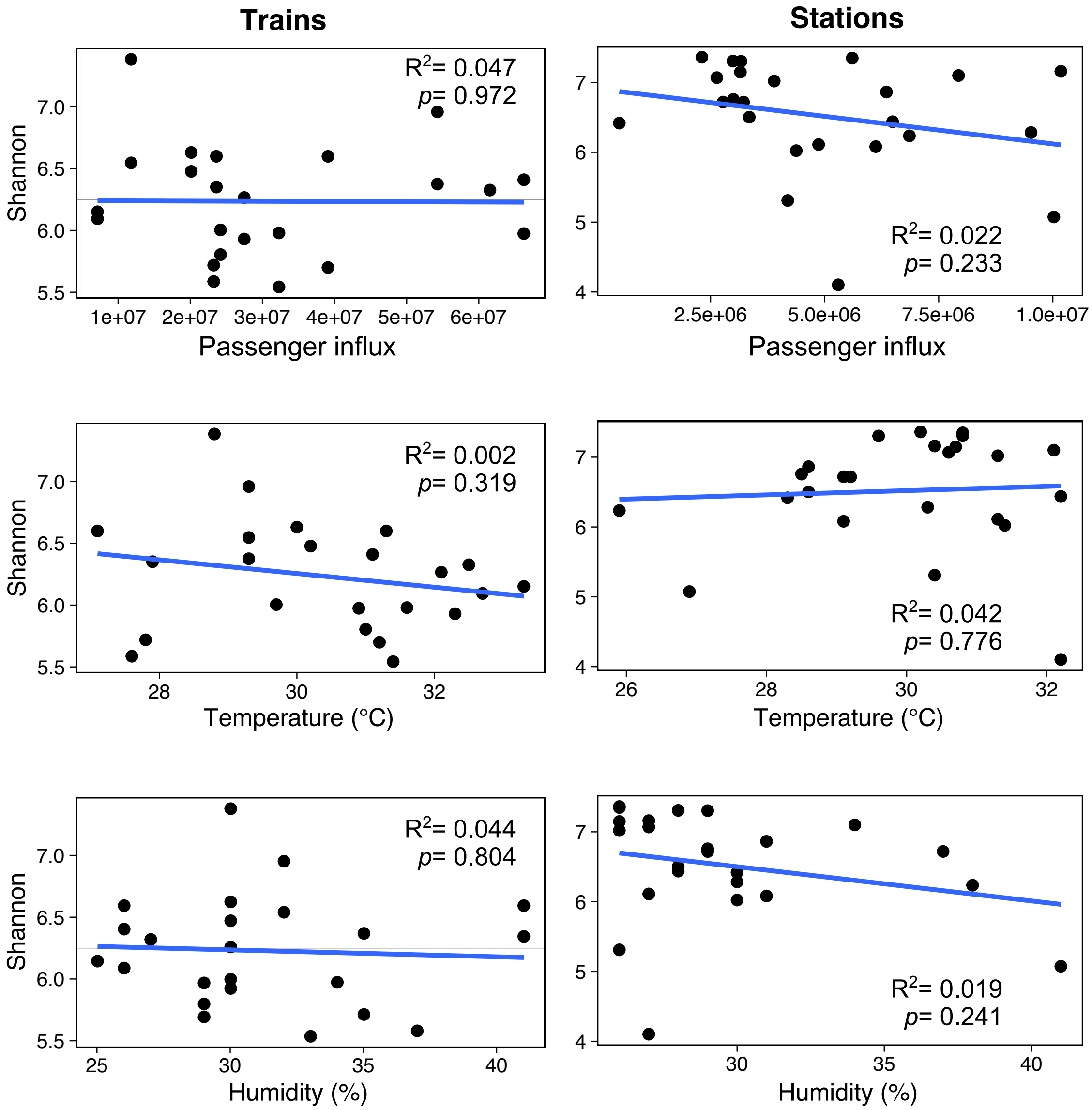

Fig. S2. Association between temperature, humidity, or passenger influx with Shannon diversity in handrails and turnstiles. A linear model was performed for each comparison. The mean temperature and humidity of the sampled points were 30.2 °C and 30.4%. There are no significant correlations between Shannon diversity and the measured metadata in both datasets of trains and stations ($p > 0.05$, Kruskal-Wallis).

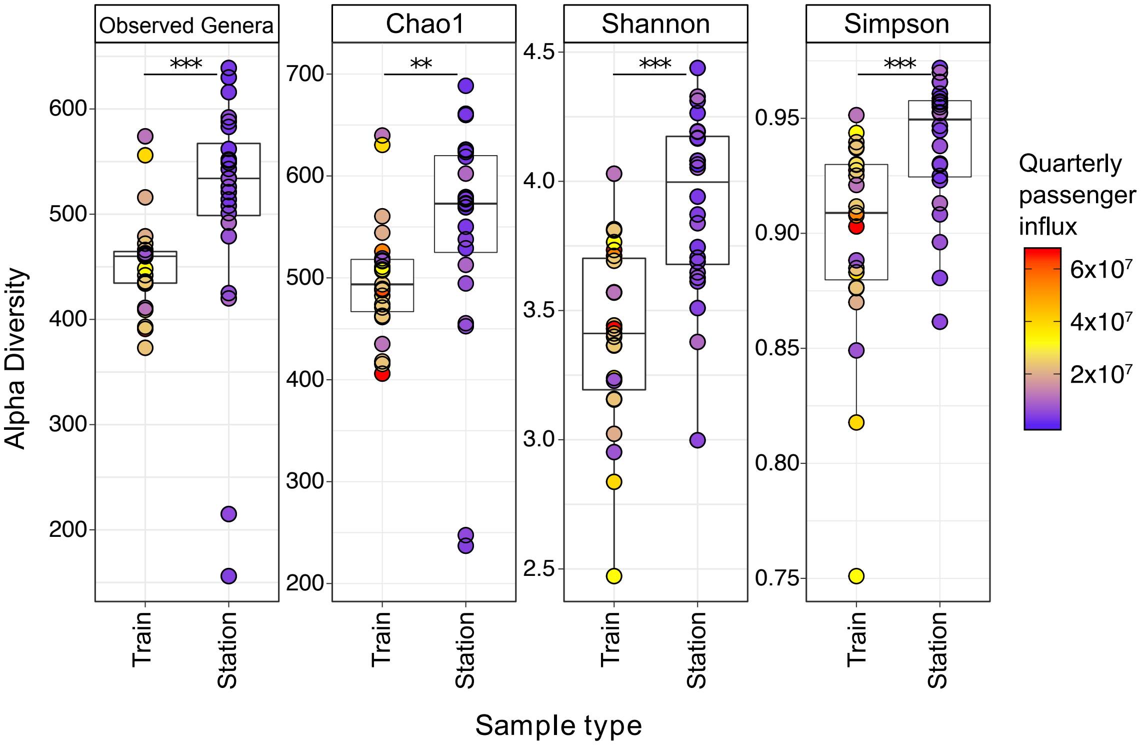

Fig. S3. **Genera richness and diversity of Mexico City's metro.** Observed genera, Chao1, Shannon, and Simpson diversity indexes showed that stations median microbial diversity is higher than trains (**$p < 0.01$, ***$p < 0.001$, Kruskal-Wallis).

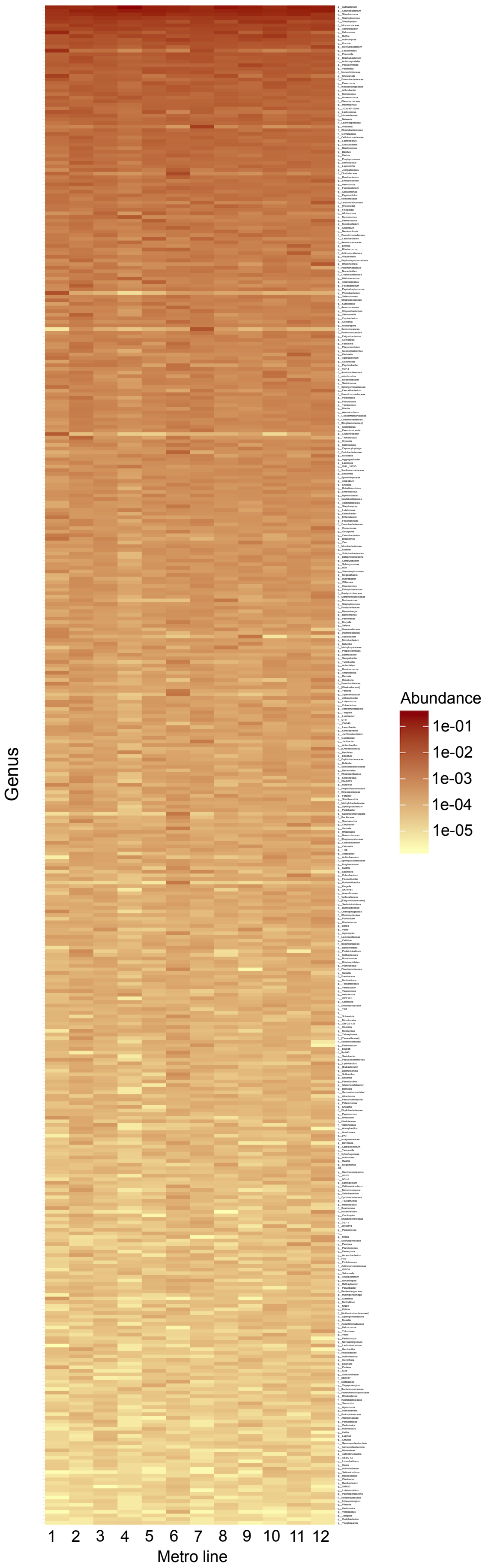

Fig. S4. Heatmap of 420 core genera and their abundances in each metro line. The unknown genera were classified to the higher known taxononomic rank.

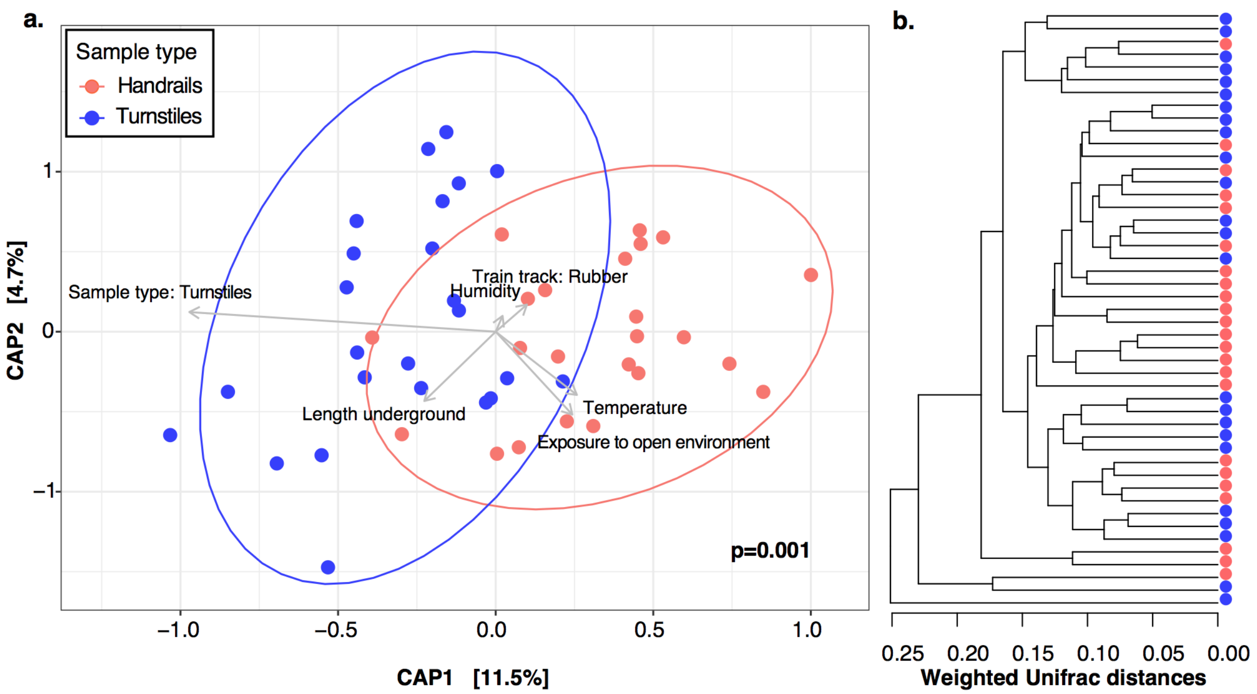

Fig. S5. Beta diversity based on weighted UniFrac distances for turnstiles and handrails at the OTU level. a. Canonical Analysis of Principal Coordinates (CAP) "sample type" significantly segregated (p = 0.001, Adonis), ellipses denote the 95% confidence interval of the points distribution by sample type. **b.** Dendrogram tends to group samples by "sample type."

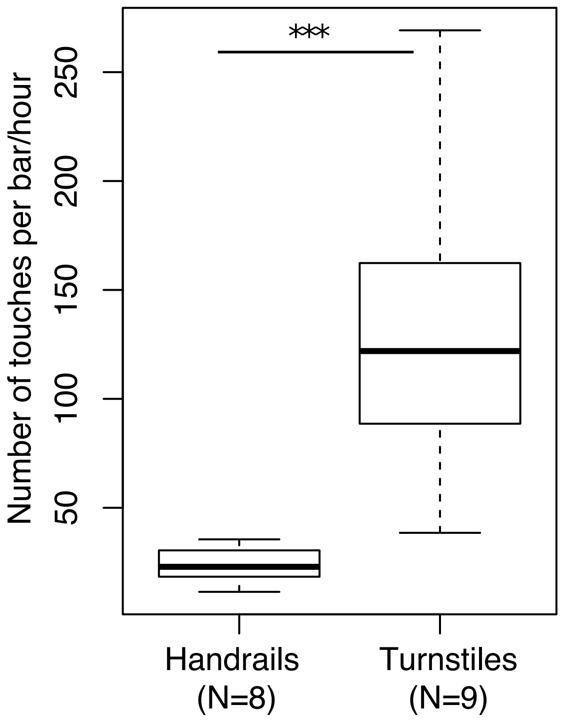

Fig S6. Handrails and turnstiles touch rate at peak hour. The subway users touched turnstiles at a higher rate than handrails (***p = 0.001, Kruskal-Wallis test).

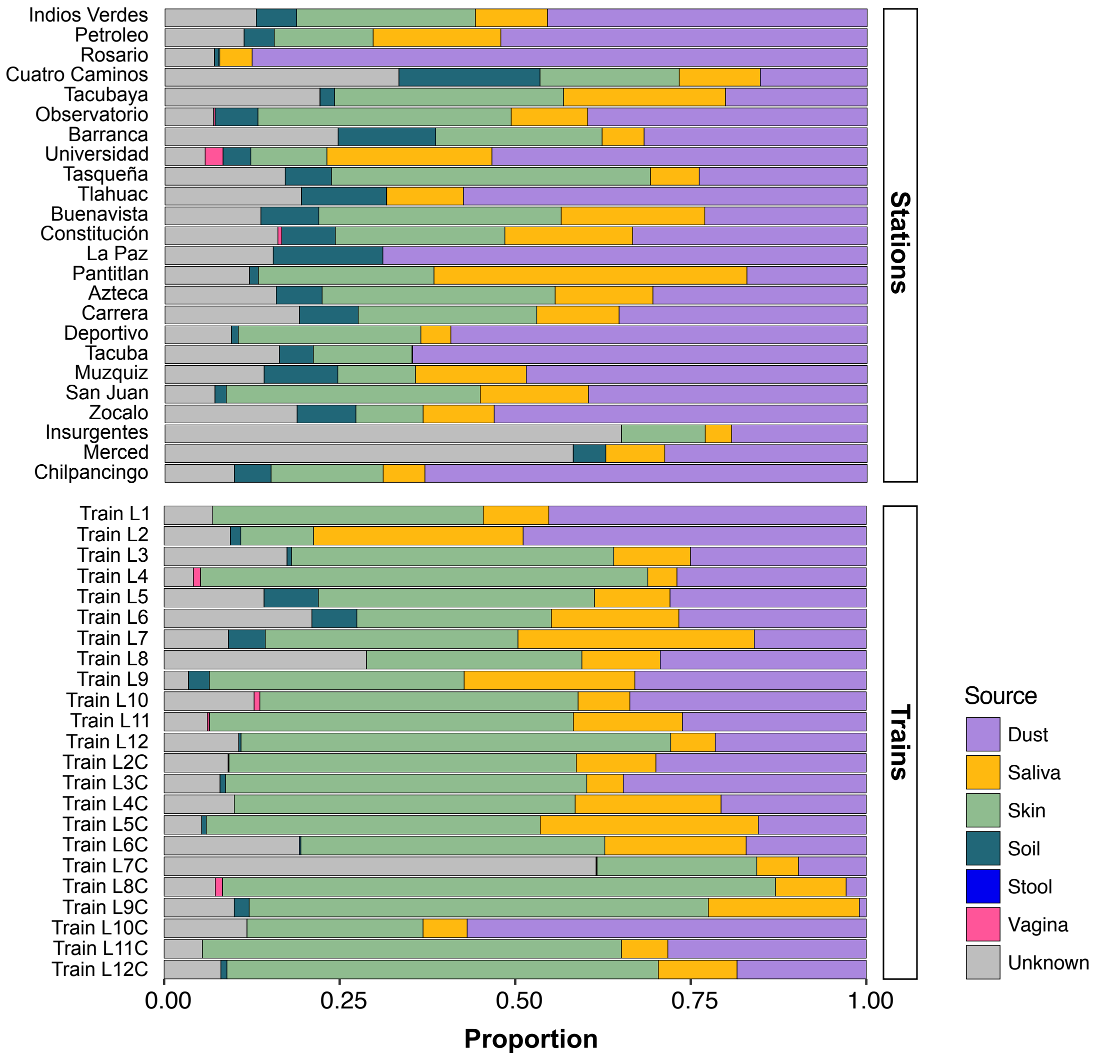

Fig. S7. Source tracking of stations and train microbiota. Source Tracker algorithm was performed at the genus level using dust, soil, saliva, skin, vagina, and feces samples as a source. A substantial proportion of microbiota appears to be from skin and dust.

Table S1. Sequencing effort, pair-end merged sequences, and OTUs summary.

N samples	47
Reads	16,631,760
Merged sequences	5,788,162
Average sequences/sample	123,152 ± 28,372
OTUs	50,174
Genera	1,058

Table S2. ASVs summary.

N samples	47
Sequences input	8,315,880
Denoised	7,159,356
Merged sequences	5,660,805
Nonchimera sequences	4,375,314
ASVs	22,673
Genera	1,252

Table S3. Alpha diversity (OTUs and Genera).

Metric	Level	Site	Min.	Median	Mean	Max.	*Median (p value*)*	*Dispersion (p value**)*
Observed	OTUs	Handrail	5417.00	9433.00	9254.22	13152.00	*0.081*	*0.001‡*
		Turnstile	428.00	10895.50	10525.83	16179.00		
	Genus	Handrail	373.00	460.00	454.91	574.00	*0.001‡*	*0.094*
		Turnstile	156.00	534.00	509.58	639.00		
Chao1	OTUs	Handrail	8395.82	15260.65	14629.63	18649.01	*0.148*	*0.001‡*
		Turnstile	840.25	16785.23	15631.35	23052.14		
	Genus	Handrail	406.00	493.62	498.99	639.70	*0.003‡*	*0.337*
		Turnstile	237.05	572.83	547.51	688.52		
Shannon	OTUs	Handrail	5.54	6.27	6.24	7.38	*0.024‡*	*0.106*
		Turnstile	4.10	6.72	6.52	7.36		
	Genus	Handrail	2.47	3.41	3.40	4.03	*4.00E-05‡*	*9.02E-01‡*
		Turnstile	3.00	4.00	3.91	4.44		
Simpson	OTUs	Handrail	0.96	0.98	0.98	0.99	*0.004‡*	*0.929*
		Turnstile	0.90	0.99	0.98	1.00		
	Genus	Handrail	0.75	0.91	0.90	0.95	*3.50E-04‡*	*1.34E-01‡*
		Turnstile	0.86	0.95	0.94	0.97		

*Kruskal-Wallis test

**Fligner-Killeen Test of Homogeneity of Variances

‡ Sadistically significant

Table S4. Core genera found in the Mexico City's metro. Numbers represent OTU count for each identified genus. Taxonomy assignations to the best taxonomy rank are described.

Genus_id	Line 1	Line 12	Line 2	Line 3	Line 4	Line 5	Line 6	Line 7	Line 8	Line 9	Line 10	Line 11	Line 12	Phylum	Class	Order	Family	Genus
1	48409	49419	51058	77484	52779	43687	39479	37965	103245	55524	40892	96839	49419	p__Actinobacteria	c__Actinobacteria	o__Actinomycetales	f__Propionibacteriacea	g__Propionibacterium
2	33182	51507	78715	63822	29519	46375	40081	41013	58743	45964	47656	78808	51507	p__Actinobacteria	c__Actinobacteria	o__Actinomycetales	f__Corynebacteriaceae	g__Corynebacterium
3	30101	17795	49139	53718	14132	37777	23178	33488	39314	38278	39792	66832	17795	p__Firmicutes	c__Bacilli	o__Lactobacillales	f__Streptococcaceae	g__Streptococcus
4	27417	18501	23783	34315	10751	14567	12331	19206	28945	17311	21395	26821	18501	p__Firmicutes	c__Bacilli	o__Bacillales	f__Staphylococcaceae	g__Staphylococcus
5	20239	7605	19653	25407	5390	10315	4669	16494	8235	23041	31563	10327	7605	p__Cyanobacteria	c__Chloroplast	o__Streptophyta	f__	g__
6	9030	12338	28461	16754	4368	10533	12411	13595	15596	8114	11972	19321	12338	p__Actinobacteria	c__Actinobacteria	o__Actinomycetales	f__Micrococcaceae	g__
7	13599	13329	14210	20429	6647	7324	5752	10264	11979	7714	16651	16002	13329	p__Proteobacteria	c__Gammaproteobacte	o__Pseudomonadales	f__Moraxellaceae	g__Acinetobacter
8	52575	1471	6836	12063	2632	2274	6925	9418	23433	2416	12100	7721	1471	p__Proteobacteria	c__Gammaproteobacte	o__Oceanospirillales	f__Halomonadaceae	g__Halomonas
9	5662	4675	20979	7274	4963	7516	3904	7810	9100	11504	22885	10069	4675	p__Actinobacteria	c__Actinobacteria	o__Actinomycetales	f__Micrococcaceae	g__Rothia
10	5965	3969	8402	6907	1374	5828	11733	8476	7368	8862	7534	9077	3969	p__Actinobacteria	c__Actinobacteria	o__Actinomycetales	f__Actinomycetaceae	g__Actinomyces
11	6362	2453	10570	6901	1640	3472	5476	5561	6986	3847	5600	8071	2453	p__Actinobacteria	c__Actinobacteria	o__Actinomycetales	f__Micrococcaceae	g__Kocuria
12	37080	1578	1635	1779	132	1169	329	9668	2808	879	476	1374	1578	p__Firmicutes	c__Bacilli	o__Lactobacillales	f__Leuconostocaceae	g__Leuconostoc
13	3419	8860	8926	4981	1059	4837	1709	5575	6202	3375	4925	4127	8860	p__Proteobacteria	c__Alphaproteobacteri	o__Rhizobiales	f__Methylobacteriacea	g__Methylobacterium
14	3063	3096	6060	6066	1322	2911	5399	5286	5497	4161	4082	7429	3096	p__Bacteroidetes	c__Bacteroidia	o__Bacteroidales	f__Prevotellaceae	g__Prevotella
15	3438	4026	10802	3800	915	2252	4586	5771	3306	3167	4612	5952	4026	p__Actinobacteria	c__Actinobacteria	o__Actinomycetales	f__Dermabacteraceae	g__Brachybacterium
16	3720	2739	10061	4838	1224	2610	3377	5722	4421	2533	4188	6019	2739	p__Actinobacteria	c__Actinobacteria	o__Actinomycetales	f__	g__
17	4446	3598	4441	5286	1013	1467	1720	2251	3643	8872	6104	4892	3598	p__Proteobacteria	c__Gammaproteobacte	o__Pseudomonadales	f__Pseudomonadacea	g__Pseudomonas
18	15924	651	2103	3593	933	650	2313	2962	9048	779	3913	2812	651	p__Proteobacteria	c__Gammaproteobacte	o__Alteromonadales	f__Shewanellaceae	g__Shewanella
19	2432	3162	6973	2863	768	4468	3403	5601	2869	1772	4657	5475	3162	p__Actinobacteria	c__Actinobacteria	o__Actinomycetales	f__Nocardioidaceae	g__
20	3035	2232	3402	4106	1244	3947	3907	4770	4201	3338	3530	5926	2232	p__Firmicutes	c__Clostridia	o__Clostridiales	f__Veillonellaceae	g__Veillonella
21	5629	2447	2398	4250	1235	1172	1021	2074	3115	3525	2283	10195	2447	p__Proteobacteria	c__Gammaproteobacte	o__Enterobacteriales	f__Enterobacteriaceae	g__
22	2180	2111	3536	3788	526	3708	1911	3927	3877	1417	5786	4545	2111	p__Proteobacteria	c__Alphaproteobacteri	o__Rhodobacterales	f__Rhodobacteraceae	g__Paracoccus
23	2801	1815	5318	3666	726	2070	3414	3343	3050	1711	2495	4131	1815	p__Actinobacteria	c__Actinobacteria	o__Actinomycetales	f__Micrococcaceae	g__Arthrobacter
24	1677	2552	5527	3387	559	2198	3556	2919	2194	1438	5039	3489	2552	p__Actinobacteria	c__Actinobacteria	o__Actinomycetales	f__Intrasporangiaceae	g__
25	1342	2158	3942	5496	1032	2257	2245	1634	4156	2122	1882	4487	2158	p__Actinobacteria	c__Actinobacteria	o__Actinomycetales	f__Micrococcaceae	g__Micrococcus
26	2463	1287	2631	5151	960	1690	1733	2097	3484	2179	2060	3931	1287	p__Firmicutes	c__Bacilli	o__Bacillales	f__Planococcaceae	g__
27	4147	1708	1536	3245	2006	1549	1161	1876	3354	1673	3429	2560	1708	p__Firmicutes	c__Clostridia	o__Clostridiales	f__[Tissierellaceae]	g__Anaerococcus
28	3116	1514	2545	2146	551	1871	1766	2556	4263	2927	1877	2350	1514	p__Proteobacteria	c__Gammaproteobacte	o__Pasteurellales	f__Pasteurellaceae	g__Haemophilus
29	5855	930	6298	1903	395	3620	305	3318	1195	933	594	1764	930	p__Firmicutes	c__Bacilli	o__Lactobacillales	f__Streptococcaceae	g__Lactococcus
30	1064	2023	4926	1758	474	1928	1818	3225	1686	832	3924	3234	2023	p__Chloroflexi	c__Thermomicrobia	o__JG30-KF-CM45	f__	g__
31	2798	1149	4657	4346	483	699	1694	1373	1617	3258	1293	3418	1149	p__Proteobacteria	c__Gammaproteobacte	o__Pseudomonadales	f__Moraxellaceae	g__
32	1753	2250	2751	2213	545	1357	2243	1724	2757	2680	1349	2918	2250	p__Proteobacteria	c__Betaproteobacteria	o__Neisseriales	f__Neisseriaceae	g__Neisseria
33	1146	1266	2678	3535	699	1258	1611	2253	2249	1883	1924	4017	1266	p__Firmicutes	c__Clostridia	o__Clostridiales	f__Lachnospiraceae	g__
34	1664	1450	5068	1854	282	1223	1401	2931	2208	996	2060	2612	1450	p__Actinobacteria	c__Actinobacteria	o__Actinomycetales	f__Cellulomonadaceae	g__
35	968	1297	1985	3017	520	2304	1016	2284	2605	843	3003	3177	1297	p__Proteobacteria	c__Alphaproteobacteri	o__Rhodobacterales	f__Rhodobacteraceae	g__
36	628	511	563	1252	357	536	108	16442	376	746	205	548	511	p__Firmicutes	c__Bacilli	o__Lactobacillales	f__Leuconostocaceae	g__Weissella
37	3046	1135	2169	1958	586	1730	1582	1701	2280	2260	1931	1839	1135	p__Firmicutes	c__Bacilli	o__Gemellales	f__Gemellaceae	g__
38	1505	1351	3250	2098	453	2281	1318	1866	2622	1218	938	2353	1351	p__Firmicutes	c__Bacilli	o__Lactobacillales	f__Lactobacillaceae	g__Lactobacillus
39	1299	830	2226	2180	562	1960	1424	1759	3023	2072	1500	1927	830	p__Firmicutes	c__Bacilli	o__Lactobacillales	f__Carnobacteriaceae	g__Granulicatella
40	967	1103	3880	1229	195	1450	1251	2260	1482	1113	2368	2581	1103	p__Actinobacteria	c__Actinobacteria	o__Actinomycetales	f__Geodermatophilace	g__Blastococcus
41	500	1118	4778	1978	187	1259	997	1286	1483	982	996	3072	1118	p__[Thermi]	c__Deinococci	o__Deinococcales	f__Deinococcaceae	g__Deinococcus
42	1079	1817	2952	2048	663	960	1565	1242	1200	1033	1643	2145	1817	p__Firmicutes	c__Bacilli	o__Bacillales	f__Bacillaceae	g__Bacillus
43	626	967	3193	1239	337	1252	2288	2023	1326	603	1742	1925	967	p__Actinobacteria	c__Actinobacteria	o__Actinomycetales	f__Dietziaceae	g__Dietzia
44	1366	1135	2194	1527	563	1352	814	1686	1694	1274	1682	1892	1135	p__Bacteroidetes	c__Bacteroidia	o__Bacteroidales	f__Porphyromonadace	g__Porphyromonas
45	2048	628	4258	1734	216	511	918	1361	1438	1083	854	1437	628	p__Firmicutes	c__Bacilli	o__Lactobacillales	f__Aerococcaceae	g__Aerococcus
46	322	773	2775	2912	474	467	2369	563	1715	822	1982	1299	773	p__Firmicutes	c__Bacilli	o__Bacillales	f__Staphylococcaceae	g__Jeotgalicoccus
47	1028	962	1411	1229	374	977	1417	1405	1181	1964	1614	2534	962	p__Fusobacteria	c__Fusobacteriia	o__Fusobacteriales	f__Leptotrichiaceae	g__Leptotrichia
48	701	1167	2456	1552	242	695	2097	1270	1101	857	1417	2538	1167	p__Actinobacteria	c__Actinobacteria	o__Actinomycetales	f__Brevibacteriaceae	g__Brevibacterium
49	1060	2356	1728	2000	402	992	446	828	1794	822	1103	2144	2356	p__Proteobacteria	c__Gammaproteobacte	o__Pseudomonadales	f__Moraxellaceae	g__Enhydrobacter
50	1351	963	943	1208	241	1026	1824	1747	1550	998	1215	1520	963	p__Fusobacteria	c__Fusobacteriia	o__Fusobacteriales	f__Fusobacteriaceae	g__Fusobacterium
51	838	817	2662	848	195	1521	1030	1514	816	693	1194	2164	817	p__Actinobacteria	c__Actinobacteria	o__Actinomycetales	f__Cellulomonadaceae	g__Cellulomonas

52	2481	610	994	307	77	303	7262	340	419	191	778	446	610	p__Firmicutes	c__Clostridia	o__Clostridiales	f__Clostridiaceae	g__
53	1484	741	1137	1486	716	729	514	1076	1702	893	1554	1313	741	p__Firmicutes	c__Clostridia	o__Clostridiales	f__[Tissierellaceae]	g__Peptoniphilus
54	1435	1112	734	1762	290	622	1154	730	1829	1182	861	1137	1112	p__Proteobacteria	c__Betaproteobacteria	o__Neisseriales	f__Neisseriaceae	g__
55	1044	507	1163	1244	251	645	1204	758	2985	1124	627	1215	507	p__Bacteroidetes	c__Bacteroidia	o__Bacteroidales	f__[Paraprevotellaceae	g__[Prevotella]
56	1564	3231	1176	964	150	382	239	2322	490	403	309	980	3231	p__Firmicutes	c__Bacilli	o__Lactobacillales	f__Leuconostocaceae	g__
57	234	245	548	3008	62	2070	658	1869	1159	309	1196	430	245	p__Firmicutes	c__Bacilli	o__Lactobacillales	f__Aerococcaceae	g__Alloiococcus
58	1113	904	763	1522	582	839	441	842	1306	850	1010	1264	904	p__Firmicutes	c__Clostridia	o__Clostridiales	f__[Tissierellaceae]	g__Finegoldia
59	933	903	1905	654	307	797	685	1286	882	556	927	1067	903	p__Actinobacteria	c__Actinobacteria	o__Actinomycetales	f__Mycobacteriaceae	g__Mycobacterium
60	1417	413	675	1258	232	327	210	585	765	476	488	4009	413	p__Proteobacteria	c__Gammaproteobacte	o__Enterobacteriales	f__Enterobacteriaceae	g__Erwinia
61	509	2053	1241	731	159	2463	518	591	505	470	534	947	2053	p__Actinobacteria	c__Actinobacteria	o__Actinomycetales	f__Dermacoccaceae	g__Dermacoccus
62	477	519	1202	1120	233	453	554	889	1981	435	1661	1104	519	p__Actinobacteria	c__Actinobacteria	o__Actinomycetales	f__Micrococcaceae	g__Nesterenkonia
63	457	180	1152	596	83	353	448	792	530	806	534	4520	180	p__Actinobacteria	c__Actinobacteria	o__Actinomycetales	f__Actinomycetaceae	g__
64	1036	395	2036	1758	1817	237	327	354	821	385	552	695	395	p__Firmicutes	c__Bacilli	o__Bacillales	f__Staphylococcaceae	g__Macrococcus
65	608	522	1611	526	181	978	2064	1036	616	300	599	1339	522	p__Firmicutes	c__Clostridia	o__Clostridiales	f__Clostridiaceae	g__Clostridium
66	863	524	737	1867	180	259	575	358	930	1505	1273	1166	524	p__Proteobacteria	c__Gammaproteobacte	o__Pseudomonadales	f__Pseudomonadacea	g__
67	831	666	756	1078	720	279	346	612	1693	409	1095	1021	666	p__Proteobacteria	c__Gammaproteobacte	o__Aeromonadales	f__Aeromonadaceae	g__
68	1522	289	1284	1129	207	424	417	1066	796	454	850	979	289	p__Bacteroidetes	c__Flavobacteriia	o__Flavobacteriales	f__[Weeksellaceae]	g__Wautersiella
69	855	397	474	661	83	2212	255	2195	738	583	339	521	397	p__Firmicutes	c__Bacilli	o__Lactobacillales	f__	g__
70	281	661	2389	368	173	501	983	775	564	369	1110	975	661	p__Firmicutes	c__Clostridia	o__Clostridiales	f__Peptostreptococcac	g__
71	372	535	4458	652	91	229	403	446	457	183	387	691	535	p__Bacteroidetes	c__Flavobacteriia	o__Flavobacteriales	f__Flavobacteriaceae	g__Flavobacterium
72	1360	68	657	1222	317	214	575	943	1511	318	925	764	68	p__Proteobacteria	c__Gammaproteobacte	o__Oceanospirillales	f__Halomonadaceae	g__
73	622	571	1216	479	109	1386	1649	601	480	429	307	945	571	p__Actinobacteria	c__Actinobacteria	o__Actinomycetales	f__Nocardiaceae	g__Rhodococcus
74	393	766	1807	440	118	616	544	1101	551	289	1073	899	766	p__Actinobacteria	c__Actinobacteria	o__Actinomycetales	f__Nocardioidaceae	g__Nocardioides
75	640	775	827	1147	75	537	549	579	700	832	642	1015	775	p__Proteobacteria	c__Betaproteobacteria	o__Burkholderiales	f__Oxalobacteraceae	g__
76	7555	34	89	44	2	17	91	147	85	62	34	65	34	p__Proteobacteria	c__Gammaproteobacte	o__Vibrionales	f__Vibrionaceae	g__Photobacterium
77	421	3458	382	1047	65	200	191	262	571	109	719	538	3458	p__Proteobacteria	c__Gammaproteobacte	o__Alteromonadales	f__[Chromatiaceae]	g__Rheinheimera
78	204	260	3702	426	58	573	431	598	174	242	441	602	260	p__Proteobacteria	c__Alphaproteobacteri	o__Rhodospirillales	f__Rhodospirillaceae	g__Skermanella
79	301	191	1268	749	144	421	714	661	632	713	1121	745	191	p__Firmicutes	c__Clostridia	o__Clostridiales	f__Peptostreptococcac	g__Peptostreptococcus
80	715	242	1721	376	75	2017	273	591	304	550	215	558	242	p__Actinobacteria	c__Actinobacteria	o__Actinomycetales	f__Intrasporangiaceae	g__Arsenicicoccus
81	1812	239	530	1022	101	393	470	526	821	572	247	811	239	p__Firmicutes	c__Bacilli	o__Lactobacillales	f__Streptococcaceae	g__
82	133	611	1556	805	226	992	271	598	589	303	290	846	611	p__Actinobacteria	c__Actinobacteria	o__Actinomycetales	f__Intrasporangiaceae	g__Kytococcus
83	876	508	565	588	53	672	339	1259	651	437	509	609	508	p__Bacteroidetes	c__Flavobacteriia	o__Flavobacteriales	f__[Weeksellaceae]	g__Chryseobacterium
84	359	281	648	560	124	515	952	525	514	817	821	924	281	p__Firmicutes	c__Clostridia	o__Clostridiales	f__Veillonellaceae	g__Selenomonas
85	982	242	257	438	64	135	112	293	312	245	144	3663	242	p__Proteobacteria	c__Gammaproteobacte	o__Enterobacteriales	f__Enterobacteriaceae	g__Klebsiella
86	207	328	882	1108	1135	261	320	534	495	264	534	802	328	p__Actinobacteria	c__Actinobacteria	o__Bifidobacteriales	f__Bifidobacteriaceae	g__Bifidobacterium
87	789	562	1382	528	265	423	336	700	358	270	473	773	562	p__Actinobacteria	c__Actinobacteria	o__Actinomycetales	f__Microbacteriaceae	g__Cryobacterium
88	463	685	539	523	85	317	1460	415	457	596	533	716	685	p__Firmicutes	c__Bacilli	o__Lactobacillales	f__Aerococcaceae	g__
89	225	486	862	283	117	219	1248	640	452	166	285	1779	486	p__Actinobacteria	c__Actinobacteria	o__Actinomycetales	f__Gordoniaceae	g__Gordonia
90	555	352	1033	714	225	405	428	568	655	370	432	883	352	p__Actinobacteria	c__Actinobacteria	o__Actinomycetales	f__Micrococcaceae	g__Microbispora
91	3	26	376	67	7	29	44	5492	143	22	117	57	26	p__Cyanobacteria	c__Oscillatoriophycidea	o__Chroococcales	f__Xenococcaceae	g__
92	232	417	1697	408	135	268	759	496	475	324	371	732	417	p__Firmicutes	c__Bacilli	o__Bacillales	f__Planococcaceae	g__Planomicrobium
93	372	1086	569	709	90	221	299	496	743	231	891	562	1086	p__Firmicutes	c__Bacilli	o__Bacillales	f__[Exiguobacteraceae	g__Exiguobacterium
94	209	645	922	603	203	183	413	1080	279	212	779	739	645	p__Firmicutes	c__Clostridia	o__Clostridiales	f__Ruminococcaceae	g__
95	483	368	1327	277	23	451	478	554	397	286	562	1037	368	p__Actinobacteria	c__Actinobacteria	o__Actinomycetales	f__Geodermatophilace	g__Geodermatophilus
96	500	284	549	896	285	424	445	393	747	510	416	684	284	p__Firmicutes	c__Bacilli	o__Gemellales	f__	g__
97	528	154	1303	314	33	320	275	431	395	230	1510	519	154	p__Proteobacteria	c__Alphaproteobacteri	o__Rhodospirillales	f__Acetobacteraceae	g__
98	473	196	994	261	51	440	353	480	332	320	268	1820	196	p__Actinobacteria	c__Actinobacteria	o__Actinomycetales	f__Geodermatophilace	g__Modestobacter
99	109	431	877	1015	237	261	156	595	882	300	223	860	431	p__Actinobacteria	c__Actinobacteria	o__Bifidobacteriales	f__Bifidobacteriaceae	g__Gardnerella
100	652	149	1304	1179	213	72	964	101	121	228	115	836	149	p__Proteobacteria	c__Gammaproteobacte	o__Pseudomonadales	f__Moraxellaceae	g__Psychrobacter
101	261	184	1481	522	365	102	1200	339	395	214	460	367	184	p__Firmicutes	c__Bacilli	o__Lactobacillales	f__Aerococcaceae	g__Facklamia
102	1582	286	607	498	283	270	163	273	542	192	579	604	286	p__Proteobacteria	c__Alphaproteobacteri	o__Rhizobiales	f__Rhizobiaceae	g__Agrobacterium
103	1367	328	484	491	57	205	190	323	604	244	679	801	328	p__Proteobacteria	c__Alphaproteobacteri	o__Sphingomonadales	f__Sphingomonadacea	g__
104	519	253	1637	254	32	265	365	801	287	233	437	552	253	p__Actinobacteria	c__Actinobacteria	o__Actinomycetales	f__Dermatophilaceae	g__Piscicoccus
105	413	338	732	413	60	449	396	419	419	247	1008	682	338	p__Actinobacteria	c__Actinobacteria	o__Actinomycetales	f__Intrasporangiaceae	g__Serinicoccus
106	736	170	815	202	214	377	101	775	276	291	1255	239	170	p__Proteobacteria	c__Alphaproteobacteri	o__Rickettsiales	f__mitochondria	g__
107	190	379	476	434	118	361	859	797	465	434	412	413	379	p__TM7	c__TM7-3	o__	f__	g__
108	122	1182	757	610	141	176	55	893	245	109	365	639	1182	p__Firmicutes	c__Clostridia	o__Clostridiales	f__Ruminococcaceae	g__Faecalibacterium
109	225	427	773	546	91	408	452	481	382	253	536	637	427	p__Actinobacteria	c__Actinobacteria	o__Actinomycetales	f__Intrasporangiaceae	g__Phycicoccus
110	217	475	1013	618	177	233	138	742	382	148	450	563	475	p__Firmicutes	c__Clostridia	o__Clostridiales	f__Lachnospiraceae	g__Blautia
111	150	48	3057	209	84	66	637	147	219	118	172	217	48	p__Firmicutes	c__Bacilli	o__Lactobacillales	f__Carnobacteriaceae	g__Trichococcus

112	202	579	869	626	62	260	567	455	223	231	551	477	579	p__Actinobacteria	c__Actinobacteria	o__Actinomycetales	f__Intrasporangiaceae	g__Terracoccus
113	347	212	400	454	141	440	702	396	618	430	217	714	212	p__Actinobacteria	c__Actinobacteria	o__Actinomycetales	f__Pseudonocardiacea	g__
114	317	436	948	286	61	277	369	612	287	275	531	660	436	p__Actinobacteria	c__Actinobacteria	o__Actinomycetales	f__Geodermatophilace	g__
115	324	368	991	413	121	382	473	516	287	231	278	662	368	p__Actinobacteria	c__Actinobacteria	o__Actinomycetales	f__Nocardioidaceae	g__Aeromicrobium
116	200	528	755	622	136	248	292	315	681	276	270	712	528	p__Proteobacteria	c__Betaproteobacteria	o__Burkholderiales	f__Comamonadaceae	g__
117	4415	182	31	111	5	7	31	36	43	65	6	54	182	p__Proteobacteria	c__Alphaproteobacteri	o__Rhodospirillales	f__Acetobacteraceae	g__Gluconobacter
118	143	430	868	401	127	302	479	307	514	330	479	397	430	p__Firmicutes	c__Clostridia	o__Clostridiales	f__[Mogibacteriaceae]	g__
119	256	214	667	664	108	177	605	499	352	241	447	527	214	p__Firmicutes	c__Clostridia	o__Clostridiales	f__	g__
120	178	252	795	288	74	321	303	1110	273	172	283	569	252	p__Actinobacteria	c__Actinobacteria	o__Actinomycetales	f__Pseudonocardiacea	g__Pseudonocardia
121	373	342	815	324	86	351	197	597	300	351	316	416	342	p__Actinobacteria	c__Actinobacteria	o__Actinomycetales	f__Microbacteriaceae	g__Cryocola
122	74	197	627	646	45	252	764	368	676	92	341	363	197	p__Firmicutes	c__Bacilli	o__Bacillales	f__Staphylococcaceae	g__Salinicoccus
123	284	603	274	329	67	142	255	496	281	639	276	560	603	p__Bacteroidetes	c__Flavobacteriia	o__Flavobacteriales	f__Flavobacteriaceae	g__Capnocytophaga
124	481	209	344	252	89	434	231	499	307	417	258	365	209	p__Proteobacteria	c__Gammaproteobacte	o__Pasteurellales	f__Pasteurellaceae	g__Aggregatibacter
125	257	503	697	207	67	207	214	377	349	402	190	415	503	p__Proteobacteria	c__Betaproteobacteria	o__Burkholderiales	f__Burkholderiaceae	g__Lautropia
126	547	309	276	290	49	166	156	204	394	323	250	754	309	p__Proteobacteria	c__Gammaproteobacte	o__Xanthomonadales	f__Xanthomonadacea	g__
127	354	250	717	386	40	176	164	288	401	269	182	384	250	p__Firmicutes	c__Bacilli	o__Lactobacillales	f__Carnobacteriaceae	g__Desemzia
128	96	771	287	265	249	256	324	511	112	171	360	131	771	p__Proteobacteria	c__Gammaproteobacte	o__Pseudomonadales	f__Moraxellaceae	g__Moraxella
129	58	147	183	160	80	125	1867	55	291	149	227	186	147	p__Actinobacteria	c__Coriobacteriia	o__Coriobacteriales	f__Coriobacteriaceae	g__
130	99	325	276	494	351	169	99	211	253	167	548	371	325	p__Firmicutes	c__Clostridia	o__Clostridiales	f__[Tissierellaceae]	g__WAL_1855D
131	115	211	472	244	36	404	202	295	216	205	503	414	211	p__Actinobacteria	c__Actinobacteria	o__Actinomycetales	f__Sporichthyaceae	g__
132	113	105	268	530	131	169	178	445	418	238	277	388	105	p__Actinobacteria	c__Coriobacteriia	o__Coriobacteriales	f__Coriobacteriaceae	g__Atopobium
133	269	146	362	458	99	81	192	198	296	201	550	377	146	p__Firmicutes	c__Bacilli	o__Lactobacillales	f__Enterococcaceae	g__Enterococcus
134	166	212	356	464	52	144	431	272	224	144	466	296	212	p__Actinobacteria	c__Actinobacteria	o__Actinomycetales	f__Intrasporangiaceae	g__Knoellia
135	160	283	311	309	17	329	213	419	201	242	172	536	283	p__Proteobacteria	c__Alphaproteobacteri	o__Rhodobacterales	f__Rhodobacteraceae	g__Rubellimicrobium
136	270	74	597	176	40	419	138	489	261	212	204	280	74	p__Bacteroidetes	c__Cytophagia	o__Cytophagales	f__Cytophagaceae	g__Hymenobacter
137	225	230	367	628	72	163	157	230	236	187	315	349	230	p__Proteobacteria	c__Alphaproteobacteri	o__Caulobacterales	f__Caulobacteraceae	g__
138	1046	190	226	188	104	80	69	149	215	178	133	458	190	p__Proteobacteria	c__Gammaproteobacte	o__Enterobacteriales	f__Enterobacteriaceae	g__Enterobacter
139	802	131	718	277	29	125	49	77	193	232	61	335	131	p__Firmicutes	c__Bacilli	o__Lactobacillales	f__Carnobacteriaceae	g__Carnobacterium
140	174	107	637	241	24	258	205	524	235	179	133	308	107	p__Actinobacteria	c__Actinobacteria	o__Actinomycetales	f__Nocardioidaceae	g__Friedmanniella
141	73	381	432	200	61	273	201	303	223	85	364	398	381	p__Actinobacteria	c__Acidimicrobiia	o__Acidimicrobiales	f__	g__
142	430	196	227	236	24	254	184	203	259	144	479	347	196	p__Proteobacteria	c__Alphaproteobacteri	o__Sphingomonadales	f__Sphingomonadacea	g__Kaistobacter
143	237	155	655	238	51	119	185	402	116	153	403	242	155	p__Firmicutes	c__Bacilli	o__Lactobacillales	f__Carnobacteriaceae	g__
144	76	214	352	215	52	179	643	233	209	123	206	404	214	p__Actinobacteria	c__Actinobacteria	o__Actinomycetales	f__Streptomycetaceae	g__Streptomyces
145	1321	145	209	518	48	54	81	34	143	117	64	145	145	p__Firmicutes	c__Bacilli	o__Bacillales	f__Listeriaceae	g__Brochothrix
146	259	126	210	274	244	137	225	220	386	77	347	298	126	p__Proteobacteria	c__Gammaproteobacte	o__Xanthomonadales	f__Xanthomonadacea	g__Luteimonas
147	119	293	351	221	65	149	409	148	277	120	257	326	293	p__Actinobacteria	c__Actinobacteria	o__Actinomycetales	f__Bogoriellaceae	g__Georgenia
148	387	770	138	184	89	60	121	138	237	138	162	195	770	p__Proteobacteria	c__Betaproteobacteria	o__Burkholderiales	f__Comamonadaceae	g__Comamonas
149	228	93	273	345	119	138	260	211	246	158	144	382	93	p__Actinobacteria	c__Actinobacteria	o__Actinomycetales	f__Microbacteriaceae	g__
150	145	375	178	357	16	117	150	171	171	510	157	198	375	p__Proteobacteria	c__Alphaproteobacteri	o__Rickettsiales	f__mitochondria	g__Zea
151	198	174	296	230	13	133	150	125	204	323	161	460	174	p__Proteobacteria	c__Betaproteobacteria	o__	f__	g__
152	78	300	357	183	12	196	139	266	149	132	327	294	300	p__Actinobacteria	c__Thermoleophilia	o__Solirubrobacterales	f__	g__
153	214	129	172	333	21	145	106	150	365	107	334	346	129	p__Proteobacteria	c__Alphaproteobacteri	o__Sphingomonadales	f__Sphingomonadacea	g__Sphingomonas
154	118	186	140	348	73	214	138	353	245	117	272	167	186	p__Firmicutes	c__Clostridia	o__Clostridiales	f__Veillonellaceae	g__Dialister
155	150	114	399	325	44	70	103	373	203	51	226	276	114	p__Firmicutes	c__Clostridia	o__Clostridiales	f__Lachnospiraceae	g__Coprococcus
156	184	62	313	216	38	147	315	140	244	213	101	342	62	p__Actinobacteria	c__Actinobacteria	o__Actinomycetales	f__Actinomycetaceae	g__N09
157	93	49	416	179	29	172	148	266	267	155	173	366	49	p__Actinobacteria	c__Actinobacteria	o__Actinomycetales	f__Williamsiaceae	g__Williamsia
158	499	169	87	238	57	71	104	136	208	220	130	393	169	p__Proteobacteria	c__Gammaproteobacte	o__Xanthomonadales	f__Xanthomonadacea	g__Stenotrophomonas
159	138	158	429	112	9	14	14	247	938	42	63	138	158	p__Proteobacteria	c__Gammaproteobacte	o__Oceanospirillales	f__Oceanospirillaceae	g__Marinomonas
160	93	92	156	250	31	117	258	349	190	274	174	317	92	p__Proteobacteria	c__Epsilonproteobacte	o__Campylobacterales	f__Campylobacteracea	g__Campylobacter
161	72	126	300	338	39	152	114	186	229	84	294	333	126	p__Proteobacteria	c__Alphaproteobacteri	o__Caulobacterales	f__Caulobacteraceae	g__Phenylobacterium
162	41	91	449	93	20	196	120	300	124	280	310	209	91	p__Actinobacteria	c__Rubrobacteria	o__Rubrobacterales	f__Rubrobacteraceae	g__Rubrobacter
163	50	120	164	244	87	77	305	257	221	144	161	357	120	p__Firmicutes	c__Clostridia	o__Clostridiales	f__Veillonellaceae	g__Megasphaera
164	211	350	295	185	36	128	166	152	119	93	166	190	350	p__Proteobacteria	c__Alphaproteobacteri	o__Rhizobiales	f__Bradyrhizobiaceae	g__
165	220	163	282	369	36	108	110	187	142	83	116	269	163	p__Firmicutes	c__Bacilli	o__Bacillales	f__Planococcaceae	g__Staphylococcus
166	429	8	88	217	45	26	109	152	423	60	227	253	8	p__Proteobacteria	c__Gammaproteobacte	o__Alteromonadales	f__Shewanellaceae	g__
167	50	492	252	155	18	138	88	238	95	45	221	213	492	p__Actinobacteria	c__Actinobacteria	o__Actinomycetales	f__Micromonosporacea	g__
168	68	170	318	164	35	116	174	292	171	42	236	209	170	p__Actinobacteria	c__Actinobacteria	o__Actinomycetales	f__Beutenbergiaceae	g__Beutenbergia
169	164	104	122	201	51	106	141	179	376	180	148	204	104	p__Proteobacteria	c__Gammaproteobacte	o__Pasteurellales	f__Pasteurellaceae	g__
170	85	201	222	133	21	156	171	247	102	91	215	326	201	p__Proteobacteria	c__Alphaproteobacteri	o__Rhizobiales	f__Bradyrhizobiaceae	g__Balneimonas
171	271	150	346	140	60	104	166	132	256	96	110	128	150	p__Firmicutes	c__Clostridia	o__Clostridiales	f__[Tissierellaceae]	g__Parvimonas
172	165	79	298	168	27	179	187	234	97	115	71	331	79	p__Actinobacteria	c__Actinobacteria	o__Actinomycetales	f__Beutenbergiaceae	g__Salana
173	261	185	290	165	41	73	146	193	164	104	93	191	185	p__Actinobacteria	c__Actinobacteria	o__Actinomycetales	f__Microbacteriaceae	g__Microbacterium
174	94	88	469	206	21	92	120	221	161	113	129	186	88	p__Actinobacteria	c__Actinobacteria	o__Actinomycetales	f__Nocardioidaceae	g__Propionicimonas
175	497	300	285	137	59	96	22	137	73	42	110	138	300	p__Firmicutes	c__Clostridia	o__Clostridiales	f__Lachnospiraceae	g__[Ruminococcus]
176	167	44	594	110	113	76	52	71	96	50	75	443	44	p__Actinobacteria	c__Actinobacteria	o__Actinomycetales	f__Dermabacteraceae	g__Dermabacter

177	20	34	120	253	38	246	26	162	743	57	68	115	34	p__Proteobacteria	c__Alphaproteobacteri	o__Rhizobiales	f__Methylocystaceae	g__
178	134	111	165	434	26	91	91	115	142	342	85	117	111	p__Proteobacteria	c__Alphaproteobacteri	o__Rickettsiales	f__mitochondria	g__Nelumbo
179	688	76	166	139	10	83	34	100	132	60	161	161	76	p__Proteobacteria	c__Alphaproteobacteri	o__Rhodobacterales	f__Rhodobacteraceae	g__Amaricoccus
180	514	8	109	60	28	24	9	32	72	878	3	58	8	p__Proteobacteria	c__Alphaproteobacteri	o__Rhodospirillales	f__Acetobacteraceae	g__Acetobacter
181	60	75	141	130	23	75	441	211	151	190	162	122	75	p__Firmicutes	c__Clostridia	o__Clostridiales	f__Lachnospiraceae	g__Moryella
182	154	136	302	72	41	108	147	151	120	71	180	288	136	p__Actinobacteria	c__Actinobacteria	o__Actinomycetales	f__Sanguibacteraceae	g__Sanguibacter
183	49	169	319	187	42	31	117	203	109	84	192	242	169	p__Firmicutes	c__Clostridia	o__Clostridiales	f__Ruminococcaceae	g__Ruminococcus
184	109	159	214	119	23	128	186	148	104	60	261	201	159	p__Actinobacteria	c__Actinobacteria	o__Actinomycetales	f__Cellulomonadaceae	g__Actinotalea
185	308	28	470	88	33	129	88	95	125	100	34	179	28	p__Actinobacteria	c__Actinobacteria	o__Actinomycetales	f__Propionibacteriacea	g__Luteococcus
186	403	61	90	55	7	28	473	49	34	43	255	177	61	p__Firmicutes	c__Bacilli	o__Turicibacterales	f__Turicibacteraceae	g__Turicibacter
187	140	123	221	191	29	122	99	138	92	95	190	223	123	p__Proteobacteria	c__Alphaproteobacteri	o__Rhizobiales	f__Hyphomicrobiaceae	g__Devosia
188	37	380	343	148	17	82	13	255	192	27	16	144	380	p__Firmicutes	c__Clostridia	o__Clostridiales	f__Lachnospiraceae	g__Roseburia
189	576	130	125	51	15	72	75	148	84	54	195	129	130	p__Firmicutes	c__Bacilli	o__Bacillales	f__Paenibacillaceae	g__
190	47	236	312	97	7	117	72	137	95	48	222	243	236	p__Bacteroidetes	c__Cytophagia	o__Cytophagales	f__Cytophagaceae	g__Adhaeribacter
191	27	94	388	83	16	95	64	156	104	93	294	170	94	p__Actinobacteria	c__Actinobacteria	o__Actinomycetales	f__Pseudonocardiacea	g__Actinomycetospora
192	133	233	162	161	38	48	72	120	180	140	161	134	233	p__Bacteroidetes	c__Flavobacteriia	o__Flavobacteriales	f__[Weeksellaceae]	g__
193	66	38	166	155	22	145	53	311	72	109	172	227	38	p__[Thermi]	c__Deinococci	o__Deinococcales	f__Trueperaceae	g__Truepera
194	353	69	145	103	13	123	175	89	78	231	49	92	69	p__Firmicutes	c__Clostridia	o__Clostridiales	f__Lachnospiraceae	g__Oribacterium
195	33	85	155	150	9	135	71	269	170	58	183	182	85	p__Actinobacteria	c__Acidimicrobiia	o__Acidimicrobiales	f__C111	g__
196	167	37	328	117	13	66	168	140	157	71	72	163	37	p__Actinobacteria	c__Actinobacteria	o__Actinomycetales	f__Dermatophilaceae	g__Kineosphaera
197	136	207	95	107	18	60	64	194	92	58	254	166	207	p__Proteobacteria	c__Gammaproteobacte	o__Xanthomonadales	f__Xanthomonadacea	g__Lysobacter
198	60	105	232	117	36	93	12	118	93	331	53	197	105	p__TM7	c__TM7-3	o__CW040	f__	g__
199	65	33	155	112	36	57	614	69	94	34	43	127	33	p__Actinobacteria	c__Actinobacteria	o__Actinomycetales	f__Yaniellaceae	g__Yaniella
200	145	125	120	204	22	47	83	87	107	187	104	175	125	p__Proteobacteria	c__Betaproteobacteria	o__Burkholderiales	f__Oxalobacteraceae	g__Janthinobacterium
201	172	62	314	94	95	45	148	31	116	115	62	147	62	p__Actinobacteria	c__Actinobacteria	o__Actinomycetales	f__Microbacteriaceae	g__Leucobacter
202	24	137	65	49	5	18	800	45	35	10	33	132	137	p__Actinobacteria	c__Actinobacteria	o__Actinomycetales	f__Promicromonospora	g__Xylanimicrobium
203	73	47	325	93	9	129	128	146	155	70	116	51	47	p__Proteobacteria	c__Gammaproteobacte	o__Pasteurellales	f__Pasteurellaceae	g__Actinobacillus
204	46	106	429	99	4	63	84	184	29	28	148	112	106	p__Acidobacteria	c__[Chloracidobacteria	o__RB41	f__Ellin6075	g__
205	19	83	239	140	8	76	94	86	191	63	158	169	83	p__Proteobacteria	c__Alphaproteobacteri	o__Rhodospirillales	f__Rhodospirillaceae	g__
206	40	76	367	83	8	82	114	189	84	60	27	192	76	p__Actinobacteria	c__Actinobacteria	o__Actinomycetales	f__Kineosporiaceae	g__Kineococcus
207	38	233	303	135	6	91	68	82	28	48	134	152	233	p__Chloroflexi	c__Ellin6529	o__	f__	g__
208	55	238	124	64	23	145	46	128	99	79	129	177	238	p__Actinobacteria	c__Thermoleophilia	o__Gaiellales	f__Gaiellaceae	g__
209	24	43	57	98	37	36	51	762	37	26	77	55	43	p__Actinobacteria	c__Actinobacteria	o__Actinomycetales	f__Intrasporangiaceae	g__Janibacter
210	88	55	58	130	22	24	58	61	127	64	58	552	55	p__Proteobacteria	c__Gammaproteobacte	o__Enterobacteriales	f__Enterobacteriaceae	g__Citrobacter
211	76	70	172	173	54	54	22	119	83	25	279	166	70	p__Bacteroidetes	c__Bacteroidia	o__Bacteroidales	f__Bacteroidaceae	g__Bacteroides
212	39	60	215	124	17	62	138	36	319	54	95	112	60	p__Firmicutes	c__Clostridia	o__Clostridiales	f__Peptostreptococcac	g__Filifactor
213	15	100	432	94	11	119	65	162	61	73	56	83	100	p__Actinobacteria	c__Actinobacteria	o__Actinomycetales	f__Kineosporiaceae	g__
214	144	88	141	157	6	77	70	117	173	55	70	154	88	p__Bacteroidetes	c__Sphingobacteriia	o__Sphingobacteriales	f__Sphingobacteriacea	g__Pedobacter
215	157	57	67	170	21	238	70	67	76	52	139	135	57	p__Proteobacteria	c__Alphaproteobacteri	o__Sphingomonadales	f__Erythrobacteraceae	g__
216	74	123	332	133	10	95	67	69	79	82	88	95	123	p__Proteobacteria	c__Alphaproteobacteri	o__Rhizobiales	f__Methylobacteriacea	g__
217	39	109	219	72	33	55	80	100	111	125	214	81	109	p__Firmicutes	c__Erysipelotrichi	o__Erysipelotrichales	f__Erysipelotrichaceae	g__Bulleidia
218	24	174	509	50	18	36	108	50	59	29	89	91	174	p__Firmicutes	c__Bacilli	o__Bacillales	f__Planococcaceae	g__Sporosarcina
219	114	104	200	127	4	43	163	92	88	54	88	156	104	p__Bacteroidetes	c__Sphingobacteriia	o__Sphingobacteriales	f__Sphingobacteriacea	g__Sphingobacterium
220	78	472	61	153	10	26	35	45	96	28	140	77	472	p__Proteobacteria	c__Gammaproteobacte	o__Alteromonadales	f__[Chromatiaceae]	g__
221	14	5	243	64	35	23	177	96	101	123	223	102	5	p__Firmicutes	c__Clostridia	o__Clostridiales	f__Lachnospiraceae	g__Shuttleworthia
222	71	95	133	70	18	128	135	94	101	70	122	168	95	p__Actinobacteria	c__Actinobacteria	o__Actinomycetales	f__Propionibacteriacea	g__
223	92	51	47	55	7	13	39	19	72	49	31	705	51	p__Proteobacteria	c__Alphaproteobacteri	o__Rhizobiales	f__Brucellaceae	g__Ochrobactrum
224	41	188	75	90	18	110	114	130	49	81	153	129	188	p__Actinobacteria	c__Thermoleophilia	o__Solirubrobacterales	f__Solirubrobacteracea	g__
225	40	157	149	112	16	37	213	20	107	44	123	128	157	p__Firmicutes	c__Bacilli	o__Bacillales	f__Bacillaceae	g__
226	451	18	107	48	119	27	54	109	24	96	23	65	18	p__Bacteroidetes	c__Flavobacteriia	o__Flavobacteriales	f__Flavobacteriaceae	g__Myroides
227	91	86	85	120	161	32	68	51	99	41	137	169	86	p__Firmicutes	c__Bacilli	o__Bacillales	f__	g__
228	37	36	127	221	27	37	130	66	51	94	185	83	36	p__Proteobacteria	c__Alphaproteobacteri	o__Caulobacterales	f__Caulobacteraceae	g__Brevundimonas
229	32	198	198	112	3	21	45	76	71	46	149	131	198	p__Proteobacteria	c__Epsilonproteobacte	o__Campylobacterales	f__Campylobacteracea	g__Arcobacter
230	32	137	197	74	21	74	112	88	43	36	100	167	137	p__Actinobacteria	c__Actinobacteria	o__Actinomycetales	f__Streptomycetaceae	g__
231	61	82	101	145	58	109	42	56	81	49	87	208	82	p__Proteobacteria	c__Alphaproteobacteri	o__Rhizobiales	f__	g__
232	236	207	92	68	9	61	46	50	149	34	53	69	207	p__Bacteroidetes	c__Flavobacteriia	o__Flavobacteriales	f__[Weeksellaceae]	g__Cloacibacterium
233	65	30	129	80	22	78	99	84	115	171	67	92	30	p__Firmicutes	c__Clostridia	o__Clostridiales	f__Lachnospiraceae	g__Catonella
234	28	76	144	114	12	94	121	88	73	37	144	77	76	p__Bacteroidetes	c__Sphingobacteriia	o__Sphingobacteriales	f__Sphingobacteriacea	g__
235	54	109	153	56	6	50	80	92	98	59	86	165	109	p__Bacteroidetes	c__[Saprospirae]	o__[Saprospirales]	f__Chitinophagaceae	g__Flavisolibacter
236	2	34	171	111	92	51	12	39	14	20	313	143	34	p__Actinobacteria	c__Actinobacteria	o__Actinomycetales	f__Actinomycetaceae	g__Actinobaculum
237	62	45	136	90	28	41	127	60	97	105	119	91	45	p__Firmicutes	c__Clostridia	o__Clostridiales	f__[Mogibacteriaceae]	g__Mogibacterium
238	46	66	51	77	54	104	42	167	107	42	84	159	66	p__Firmicutes	c__Clostridia	o__Clostridiales	f__[Tissierellaceae]	g__1-68
239	29	82	215	64	1	81	72	123	10	67	62	179	82	p__Chloroflexi	c__Chloroflexi	o__AKIW781	f__	g__
240	5	22	34	7	6	5	702	9	79	19	45	30	22	p__Actinobacteria	c__Actinobacteria	o__Actinomycetales	f__Pseudonocardiacea	g__Saccharomonospor
241	14	83	232	125	8	39	39	66	75	34	190	58	83	p__Proteobacteria	c__Alphaproteobacteri	o__Rhizobiales	f__Aurantimonadacea	g__Aurantimonas

242	51	7	25	72	22	56	515	34	58	39	30	42	7	p__Firmicutes	c__Bacilli	o__Gemellales	f__Gemellaceae	g__Gemella
243	65	54	42	104	20	40	94	65	135	165	40	113	54	p__Proteobacteria	c__Betaproteobacteria	o__Neisseriales	f__Neisseriaceae	g__Kingella
244	9	4	75	25	22	144	95	349	112	11	49	41	4	p__Actinobacteria	c__Actinobacteria	o__Bifidobacteriales	f__Bifidobacteriaceae	g__Scardovia
245	237	24	49	98	54	8	226	26	35	29	45	102	24	p__Firmicutes	c__Bacilli	o__Bacillales	f__Planococcaceae	g__Kurthia
246	53	67	97	154	21	31	73	95	51	59	78	124	67	p__Firmicutes	c__Clostridia	o__Clostridiales	f__Veillonellaceae	g__
247	396	79	79	55	2	17	38	25	79	21	48	60	79	p__Bacteroidetes	c__[Saprospirae]	o__[Saprospirales]	f__Chitinophagaceae	g__
248	57	118	54	127	13	26	67	39	116	41	132	76	118	p__Firmicutes	c__Bacilli	o__Bacillales	f__[Exiguobacteraceae	g__
249	46	29	98	122	12	84	64	63	71	83	106	81	29	p__Proteobacteria	c__Betaproteobacteria	o__Burkholderiales	f__	g__
250	61	91	55	110	23	49	49	25	117	41	63	170	91	p__Proteobacteria	c__Betaproteobacteria	o__Rhodocyclales	f__Rhodocyclaceae	g__
251	76	80	96	42	21	61	99	50	90	28	70	140	80	p__Actinobacteria	c__Actinobacteria	o__Actinomycetales	f__Cellulomonadaceae	g__Sediminihabitans
252	12	173	77	51	8	15	36	44	64	33	206	113	173	p__Bacteroidetes	c__Cytophagia	o__Cytophagales	f__Cytophagaceae	g__Pontibacter
253	94	142	119	74	20	21	28	51	59	18	107	94	142	p__Proteobacteria	c__Alphaproteobacteri	o__Rhodobacterales	f__Rhodobacteraceae	g__Rhodobacter
254	50	30	36	288	5	7	25	34	56	220	15	40	30	p__Proteobacteria	c__Gammaproteobacte	o__Vibrionales	f__Vibrionaceae	g__Vibrio
255	23	67	458	12	18	8	70	64	5	15	51	3	67	p__Firmicutes	c__Clostridia	o__Clostridiales	f__Clostridiaceae	g__Proteiniclasticum
256	40	49	295	28	21	22	46	17	6	33	90	145	49	p__Acidobacteria	c__Solibacteres	o__Solibacterales	f__	g__
257	134	49	161	57	6	51	23	65	84	78	30	47	49	p__Firmicutes	c__Bacilli	o__Lactobacillales	f__Lactobacillaceae	g__
258	66	86	89	98	25	43	38	116	39	41	38	102	86	p__Firmicutes	c__Clostridia	o__Clostridiales	f__Lachnospiraceae	g__Dorea
259	77	40	95	82	18	54	41	87	45	15	85	139	40	p__Proteobacteria	c__Gammaproteobacte	o__Alteromonadales	f__Alteromonadaceae	g__Cellvibrio
260	68	14	127	57	10	89	30	112	48	61	35	111	14	p__Proteobacteria	c__Alphaproteobacteri	o__Rhizobiales	f__Beijerinckiaceae	g__
261	127	9	62	84	8	40	31	68	71	44	34	180	9	p__Proteobacteria	c__Gammaproteobacte	o__Enterobacteriales	f__Enterobacteriaceae	g__Serratia
262	15	24	11	7	6	2	635	7	12	4	13	22	24	p__Firmicutes	c__Bacilli	o__Bacillales	f__Planococcaceae	g__Rummeliibacillus
263	48	40	80	42	13	36	219	24	18	39	32	135	40	p__Actinobacteria	c__Actinobacteria	o__Actinomycetales	f__Microbacteriaceae	g__Agromyces
264	33	52	123	115	21	37	78	38	72	22	54	79	52	p__Firmicutes	c__Bacilli	o__Bacillales	f__Planococcaceae	g__Planococcus
265	26	162	54	119	6	15	58	40	104	17	36	83	162	p__Proteobacteria	c__Alphaproteobacteri	o__Rhodospirillales	f__	g__
266	45	51	131	63	5	30	55	52	132	19	58	76	51	p__Actinobacteria	c__Actinobacteria	o__Actinomycetales	f__Propionibacteriacea	g__Tessaracoccus
267	36	45	97	138	28	88	43	69	50	20	22	78	45	p__Proteobacteria	c__Alphaproteobacteri	o__Rhodospirillales	f__Acetobacteraceae	g__Roseomonas
268	116	35	197	78	39	18	26	10	35	12	24	109	35	p__Firmicutes	c__Bacilli	o__Lactobacillales	f__Enterococcaceae	g__Vagococcus
269	5	31	52	43	11	49	104	157	55	32	109	50	31	p__Bacteroidetes	c__Bacteroidia	o__Bacteroidales	f__	g__
270	26	27	144	53	1	33	29	122	62	26	71	103	27	p__Planctomycetes	c__Phycisphaerae	o__WD2101	f__	g__
271	36	25	60	53	5	59	59	69	32	72	93	127	25	p__Actinobacteria	c__Actinobacteria	o__Actinomycetales	f__Frankiaceae	g__
272	27	37	126	80	26	77	52	39	52	40	16	105	37	p__Actinobacteria	c__Actinobacteria	o__Actinomycetales	f__Intrasporangiaceae	g__Marihabitans
273	37	56	77	83	17	31	66	88	59	42	12	106	56	p__Actinobacteria	c__Actinobacteria	o__Actinomycetales	f__Actinomycetaceae	g__Varibaculum
274	148	13	238	28	2	17	20	40	45	28	23	71	13	p__Proteobacteria	c__Gammaproteobacte	o__Vibrionales	f__Pseudoalteromonac	g__Pseudoalteromonas
275	9	15	300	37	4	12	106	36	17	20	64	37	15	p__Firmicutes	c__Clostridia	o__Clostridiales	f__[Tissierellaceae]	g__
276	71	22	71	55	9	27	41	34	59	42	155	69	22	p__Firmicutes	c__Bacilli	o__Lactobacillales	f__Enterococcaceae	g__
277	3	9	260	66	32	65	35	22	30	5	54	72	9	p__Actinobacteria	c__Actinobacteria	o__Actinomycetales	f__Actinomycetaceae	g__Mobiluncus
278	38	2	98	51	2	53	49	64	75	49	64	86	2	p__Actinobacteria	c__Actinobacteria	o__Actinomycetales	f__Nakamurellaceae	g__
279	26	64	44	95	34	23	19	50	62	32	82	91	64	p__Actinobacteria	c__Coriobacteriia	o__Coriobacteriales	f__Coriobacteriaceae	g__Collinsella
280	34	29	89	118	16	22	81	27	53	32	50	68	29	p__Actinobacteria	c__Actinobacteria	o__Actinomycetales	f__Intrasporangiaceae	g__Tetrasphaera
281	40	51	102	54	16	65	42	61	71	41	13	55	51	p__Actinobacteria	c__Actinobacteria	o__Actinomycetales	f__Propionibacteriacea	g__Microlunatus
282	43	1	71	38	9	37	27	182	34	26	77	62	1	p__Actinobacteria	c__Actinobacteria	o__Actinomycetales	f__Nocardioidaceae	g__Pimelobacter
283	32	21	59	87	1	33	90	32	51	57	26	115	21	p__Actinobacteria	c__Actinobacteria	o__Actinomycetales	f__Beutenbergiaceae	g__Serinibacter
284	42	47	160	44	14	35	55	47	22	29	37	71	47	p__TM7	c__TM7-3	o__EW055	f__	g__
285	28	23	32	43	34	12	252	23	34	1	81	39	23	p__Bacteroidetes	c__Flavobacteriia	o__Flavobacteriales	f__Flavobacteriaceae	g__
286	21	68	88	61	14	15	22	80	102	16	33	80	68	p__Firmicutes	c__Erysipelotrichi	o__Erysipelotrichales	f__Erysipelotrichaceae	g__[Eubacterium]
287	40	12	29	80	19	61	106	30	72	26	41	76	12	p__Firmicutes	c__Clostridia	o__	f__	g__
288	27	165	55	45	7	47	28	24	31	17	70	69	165	p__Chloroflexi	c__Gitt-GS-136	o__	f__	g__
289	41	95	110	40	28	44	25	57	11	31	79	17	95	p__SR1	c__	o__	f__	g__
290	33	27	36	49	31	93	53	10	46	71	39	88	27	p__Synergistetes	c__Synergistia	o__Synergistales	f__Dethiosulfovibrionac	g__TG5
291	7	57	112	52	6	33	71	65	34	24	53	59	57	p__Actinobacteria	c__Actinobacteria	o__Actinomycetales	f__Dermatophilaceae	g__Dermatophilus
292	44	9	124	52	5	67	15	20	50	31	86	69	9	p__Firmicutes	c__Bacilli	o__Bacillales	f__Paenibacillaceae	g__Paenibacillus
293	20	42	42	93	89	15	22	16	111	18	47	56	42	p__Proteobacteria	c__Gammaproteobacte	o__Aeromonadales	f__Aeromonadaceae	g__Aeromonas
294	41	44	8	74	10	27	180	48	43	35	17	28	44	p__Firmicutes	c__Clostridia	o__Clostridiales	f__Veillonellaceae	g__Schwartzia
295	41	13	75	41	1	45	35	83	60	16	47	93	13	p__Proteobacteria	c__Alphaproteobacteri	o__Rhodospirillales	f__Acetobacteraceae	g__Belnapia
296	11	1	153	71	1	6	9	28	41	40	56	129	1	p__Firmicutes	c__Bacilli	o__Bacillales	f__Bacillaceae	g__Anoxybacillus
297	30	31	91	203	1	9	7	12	105	11	4	40	31	p__Proteobacteria	c__Gammaproteobacte	o__Vibrionales	f__Vibrionaceae	g__
298	24	71	89	43	5	18	116	40	17	15	25	56	71	p__Actinobacteria	c__Actinobacteria	o__Actinomycetales	f__Nocardiaceae	g__Nocardia
299	32	45	69	64	8	2	82	28	18	60	43	62	45	p__Proteobacteria	c__Gammaproteobacte	o__Enterobacteriales	f__Enterobacteriaceae	g__Gluconacetobacter
300	61	4	15	76	20	10	24	64	115	21	36	63	4	p__Proteobacteria	c__Gammaproteobacte	o__Oceanospirillales	f__Alcanivoracaceae	g__Alcanivorax
301	204	45	95	41	5	20	9	13	4	14	28	22	45	p__Proteobacteria	c__Alphaproteobacteri	o__Rhizobiales	f__Rhizobiaceae	g__Rhizobium
302	19	26	103	36	5	34	29	57	68	37	46	38	26	p__Proteobacteria	c__Betaproteobacteria	o__Burkholderiales	f__Comamonadaceae	g__Polaromonas
303	14	116	90	17	1	34	29	33	20	9	77	56	116	p__Gemmatimonadete	c__Gemmatimonadetes	o__Gemmatimonadales	f__	g__
304	26	66	3	20	6	21	210	43	35	26	24	15	66	p__TM7	c__TM7-3	o__I025	f__Rs-045	g__
305	38	136	86	18	41	10	76	28	25	11	10	14	136	p__Firmicutes	c__Bacilli	o__Bacillales	f__Planococcaceae	g__Lysinibacillus
306	46	46	61	48	4	6	57	43	42	23	57	48	46	p__Proteobacteria	c__Alphaproteobacteri	o__Rhizobiales	f__Phyllobacteriaceae	g__

307	57	14	70	69	31	25	32	25	39	29	21	69	14	p__Actinobacteria	c__Actinobacteria	o__Actinomycetales	f__Microbacteriaceae	g__Pseudoclavibacter
308	18	54	24	26	13	4	199	26	17	24	40	30	54	p__Firmicutes	c__Bacilli	o__Bacillales	f__Planococcaceae	g__Solibacillus
309	13	31	113	49	7	38	31	36	55	17	18	61	31	p__Actinobacteria	c__Actinobacteria	o__Actinomycetales	f__Micrococcaceae	g__Acaricomes
310	12	2	175	10	2	18	103	19	64	2	3	52	2	p__Actinobacteria	c__Actinobacteria	o__Actinomycetales	f__Dermabacteraceae	g__Devriesea
311	6	12	102	15	16	59	22	71	23	9	91	34	12	p__Planctomycetes	c__Planctomycetia	o__Pirellulales	f__Pirellulaceae	g__
312	41	51	56	38	17	27	53	18	36	24	43	46	51	p__Firmicutes	c__Clostridia	o__Clostridiales	f__Peptococcaceae	g__Peptococcus
313	19	38	101	68	4	19	18	38	59	23	21	36	38	p__Proteobacteria	c__Betaproteobacteria	o__Burkholderiales	f__Comamonadaceae	g__Acidovorax
314	12	25	26	39	4	53	16	70	27	6	58	98	25	p__Bacteroidetes	c__Cytophagia	o__Cytophagales	f__Cytophagaceae	g__
315	21	36	24	73	46	2	21	66	32	25	43	42	36	p__Fusobacteria	c__Fusobacteriia	o__Fusobacteriales	f__Leptotrichiaceae	g__Sneathia
316	27	22	15	32	11	8	34	59	20	49	40	112	22	p__Proteobacteria	c__Gammaproteobacte	o__Cardiobacteriales	f__Cardiobacteriaceae	g__Cardiobacterium
317	28	18	23	29	28	9	4	13	150	5	15	107	18	p__Firmicutes	c__Clostridia	o__Clostridiales	f__Veillonellaceae	g__Megamonas
318	11	51	196	23	1	5	21	63	18	2	5	32	51	p__Actinobacteria	c__Actinobacteria	o__Actinomycetales	f__Micromonosporacea	g__Micromonospora
319	18	60	78	24	3	50	30	29	47	28	28	32	60	p__Planctomycetes	c__Planctomycetia	o__Gemmatales	f__Isosphaeraceae	g__
320	118	11	34	36	6	9	11	18	21	11	83	65	11	p__Proteobacteria	c__Alphaproteobacteri	o__Sphingomonadales	f__Sphingomonadacea	g__Sphingobium
321	16	59	32	23	5	32	30	56	25	32	16	89	59	p__Bacteroidetes	c__Bacteroidia	o__Bacteroidales	f__Porphyromonadace	g__Tannerella
322	23	55	24	59	10	15	22	19	51	22	46	59	55	p__	c__	o__	f__	g__
323	49	12	6	13	3	3	5	15	4	19	14	259	12	p__Proteobacteria	c__Gammaproteobacte	o__Enterobacteriales	f__Enterobacteriaceae	g__Pantoea
324	2	83	27	74	13	62	19	53	7	18	15	20	83	p__Firmicutes	c__Clostridia	o__Clostridiales	f__[Tissierellaceae]	g__ph2
325	29	5	68	47	7	23	38	15	31	18	34	75	5	p__Actinobacteria	c__Actinobacteria	o__Actinomycetales	f__Dermabacteraceae	g__Helcobacillus
326	20	61	47	60	18	9	14	16	35	34	50	25	61	p__Actinobacteria	c__Actinobacteria	o__Actinomycetales	f__Pseudonocardiacea	g__Saccharopolyspora
327	25	105	36	19	3	7	54	25	30	9	13	62	105	p__Actinobacteria	c__Actinobacteria	o__Actinomycetales	f__Ruaniaceae	g__Ruania
328	11	49	18	19	1	42	32	38	13	31	81	50	49	p__Acidobacteria	c__Acidobacteria-6	o__iii1-15	f__	g__
329	53	24	62	37	3	18	49	27	25	21	14	50	24	p__Actinobacteria	c__Actinobacteria	o__Actinomycetales	f__Microbacteriaceae	g__Salinibacterium
330	26	21	96	16	6	8	102	15	22	18	7	43	21	p__Actinobacteria	c__Actinobacteria	o__Actinomycetales	f__Promicromonospora	g__Cellulosimicrobium
331	35	5	12	59	20	7	11	16	71	6	17	113	5	p__Proteobacteria	c__Gammaproteobacte	o__Enterobacteriales	f__Enterobacteriaceae	g__Plesiomonas
332	35	36	121	34	13	1	22	20	10	10	27	39	36	p__Firmicutes	c__Clostridia	o__Clostridiales	f__Ruminococcaceae	g__Oscillospira
333	20	30	54	15	7	39	35	31	11	22	24	79	30	p__Actinobacteria	c__Actinobacteria	o__Actinomycetales	f__Tsukamurellaceae	g__Tsukamurella
334	4	123	53	12	10	12	7	27	26	38	20	34	123	p__GN02	c__BD1-5	o__	f__	g__
335	7	20	53	54	6	33	15	54	36	7	34	41	20	p__Firmicutes	c__Erysipelotrichi	o__Erysipelotrichales	f__Erysipelotrichaceae	g__
336	118	4	21	29	2	13	70	24	16	4	43	13	4	p__Actinobacteria	c__Actinobacteria	o__Actinomycetales	f__Ruaniaceae	g__
337	23	12	157	21	10	2	15	59	21	5	10	22	12	p__Actinobacteria	c__Actinobacteria	o__Actinomycetales	f__Actinomycetaceae	g__Arcanobacterium
338	21	42	53	21	2	10	26	97	13	12	8	48	42	p__TM7	c__TM7-1	o__	f__	g__
339	5	18	48	22	4	7	112	30	61	6	30	7	18	p__Bacteroidetes	c__Cytophagia	o__Cytophagales	f__Cyclobacteriaceae	g__
340	14	15	62	12	7	47	29	29	21	40	24	31	15	p__FBP	c__	o__	f__	g__
341	6	35	38	13	7	10	36	65	21	2	91	7	35	p__Actinobacteria	c__Acidimicrobiia	o__Acidimicrobiales	f__AKIW874	g__
342	9	8	41	34	10	14	30	19	37	26	23	77	8	p__Actinobacteria	c__Actinobacteria	o__Actinomycetales	f__Actinosynnematace	g__
343	8	39	64	47	1	14	46	7	48	15	24	13	39	p__Actinobacteria	c__Actinobacteria	o__Actinomycetales	f__Cellulomonadaceae	g__Demequina
344	24	11	11	22	26	7	6	11	21	26	7	147	11	p__Proteobacteria	c__Gammaproteobacte	o__Enterobacteriales	f__Enterobacteriaceae	g__Salmonella
345	2	40	76	42	5	4	10	34	23	8	27	45	40	p__Proteobacteria	c__Betaproteobacteria	o__Burkholderiales	f__Comamonadaceae	g__Hydrogenophaga
346	203	8	15	9	1	3	1	9	27	1	17	18	8	p__Proteobacteria	c__Betaproteobacteria	o__Burkholderiales	f__Alcaligenaceae	g__Sutterella
347	17	11	43	39	5	24	34	26	27	22	17	46	11	p__Actinobacteria	c__Actinobacteria	o__Actinomycetales	f__Microbacteriaceae	g__Rathayibacter
348	5	163	21	4	1	7	18	42	1	13	16	14	163	p__Actinobacteria	c__Actinobacteria	o__Actinomycetales	f__Nocardiaceae	g__
349	3	37	48	18	16	5	23	51	26	5	38	34	37	p__Firmicutes	c__Clostridia	o__Clostridiales	f__[Tissierellaceae]	g__GW-34
350	7	50	25	9	7	8	27	47	9	36	42	34	50	p__Planctomycetes	c__Planctomycetia	o__Planctomycetales	f__Planctomycetaceae	g__Planctomyces
351	30	25	35	19	4	35	28	20	34	19	21	31	25	p__Firmicutes	c__Bacilli	o__Lactobacillales	f__Aerococcaceae	g__Alkalibacterium
352	10	65	27	6	9	21	20	14	79	5	19	25	65	p__Armatimonadetes	c__[Fimbriimonadia]	o__[Fimbriimonadales]	f__[Fimbriimonadaceae	g__Fimbriimonas
353	28	7	42	33	9	18	37	22	26	19	17	41	7	p__Actinobacteria	c__Actinobacteria	o__Actinomycetales	f__Beutenbergiaceae	g__
354	37	16	64	18	4	27	51	32	15	9	2	22	16	p__Bacteroidetes	c__Bacteroidia	o__Bacteroidales	f__Porphyromonadace	g__Paludibacter
355	8	27	13	40	6	16	36	51	5	27	55	13	27	p__TM7	c__TM7-3	o__CW040	f__F16	g__
356	12	105	5	6	4	11	52	1	4	14	28	53	105	p__Actinobacteria	c__Actinobacteria	o__Actinomycetales	f__Gordoniaceae	g__Millisia
357	9	10	20	21	1	25	59	88	9	3	15	33	10	p__Actinobacteria	c__Actinobacteria	o__Actinomycetales	f__Nocardiopsaceae	g__Nocardiopsis
358	4	14	15	38	1	32	5	26	41	3	69	44	14	p__Proteobacteria	c__Betaproteobacteria	o__MND1	f__	g__
359	23	31	47	29	2	12	20	22	24	19	16	46	31	p__Proteobacteria	c__Betaproteobacteria	o__Burkholderiales	f__Comamonadaceae	g__Methylibium
360	17	109	6	14	21	16	3	20	36	11	10	17	109	p__Proteobacteria	c__Betaproteobacteria	o__Methylophilales	f__Methylophilaceae	g__
361	17	19	21	53	6	5	13	16	32	35	24	33	19	p__Proteobacteria	c__Betaproteobacteria	o__Burkholderiales	f__Oxalobacteraceae	g__Massilia
362	28	1	141	24	1	14	11	3	10	13	6	22	1	p__Firmicutes	c__Bacilli	o__Bacillales	f__Bacillaceae	g__Geobacillus
363	3	24	38	18	6	5	49	14	4	6	73	27	24	p__Actinobacteria	c__Actinobacteria	o__Actinomycetales	f__Intrasporangiaceae	g__Kribbia
364	9	31	7	38	1	16	20	25	14	20	44	39	31	p__Proteobacteria	c__Alphaproteobacteri	o__Rhizobiales	f__Aurantimonadaceae	g__
365	19	12	17	51	12	21	29	4	22	19	39	17	12	p__Firmicutes	c__Clostridia	o__Clostridiales	f__[Acidaminobacterac	g__
366	40	5	16	21	3	7	9	29	32	11	25	58	5	p__Proteobacteria	c__Alphaproteobacteri	o__Sphingomonadales	f__Sphingomonadacea	g__Novosphingobium
367	29	28	9	9	3	52	14	18	15	26	26	23	28	p__Proteobacteria	c__Alphaproteobacteri	o__Sphingomonadales	f__	g__
368	27	6	31	38	7	11	12	11	34	9	25	30	6	p__Proteobacteria	c__Alphaproteobacteri	o__Rhizobiales	f__Rhizobiaceae	g__
369	1	67	21	53	1	5	2	30	15	6	3	33	67	p__Firmicutes	c__Clostridia	o__Clostridiales	f__Lachnospiraceae	g__Lachnobacterium
370	2	29	39	26	16	9	1	21	11	42	30	10	29	p__Firmicutes	c__Clostridia	o__Clostridiales	f__[Tissierellaceae]	g__Helcococcus
371	35	1	14	10	3	29	4	17	10	52	25	32	1	p__Firmicutes	c__Bacilli	o__Lactobacillales	f__Lactobacillaceae	g__Pediococcus

372	10	8	5	21	20	5	5	15	19	10	83	29	8	p__Proteobacteria	c__Gammaproteobacte	o__Vibrionales	f__Pseudoalteromonac	g__Vibrio
373	11	22	60	2	1	12	13	27	22	7	13	40	22	p__Actinobacteria	c__Acidimicrobiia	o__Acidimicrobiales	f__EB1017	g__
374	9	23	31	12	1	9	13	11	52	21	7	40	23	p__Proteobacteria	c__Betaproteobacteria	o__Neisseriales	f__Neisseriaceae	g__Eikenella
375	9	16	64	3	1	35	7	15	26	5	10	38	16	p__TM7	c__TM7-3	o__I025	f__	g__
376	17	95	10	26	5	8	5	11	6	8	6	30	95	p__Proteobacteria	c__Gammaproteobacte	o__Aeromonadales	f__Aeromonadaceae	g__Tolumonas
377	21	40	64	7	8	4	4	3	13	18	27	14	40	p__Actinobacteria	c__Actinobacteria	o__Actinomycetales	f__Thermomonosporac	g__Actinomadura
378	93	5	4	11	2	10	7	26	12	22	15	15	5	p__Proteobacteria	c__Gammaproteobacte	o__Enterobacteriales	f__Enterobacteriaceae	g__Proteus
379	4	13	32	30	2	13	5	25	26	7	21	40	13	p__Proteobacteria	c__Alphaproteobacteri	o__Rhizobiales	f__Hyphomicrobiaceae	g__Rhodoplanes
380	13	7	11	47	3	13	27	11	11	41	14	12	7	p__Proteobacteria	c__Alphaproteobacteri	o__Rickettsiales	f__mitochondria	g__Oenothera
381	9	35	14	18	6	12	22	8	15	13	30	17	35	p__Actinobacteria	c__Thermoleophilia	o__Solirubrobacterales	f__Solirubrobacteracea	g__Solirubrobacter
382	4	13	20	20	3	16	67	3	9	8	7	26	13	p__Actinobacteria	c__Actinobacteria	o__Actinomycetales	f__Dietziaceae	g__
383	51	2	11	16	8	12	28	7	11	16	6	28	2	p__Actinobacteria	c__Actinobacteria	o__Actinomycetales	f__Promicromonospora	g__
384	9	3	5	15	4	55	6	6	16	10	46	19	3	p__Actinobacteria	c__Actinobacteria	o__Actinomycetales	f__Micromonosporacea	g__Virgisporangium
385	24	8	11	38	2	2	10	13	26	5	37	13	8	p__Proteobacteria	c__Gammaproteobacte	o__Alteromonadales	f__[Chromatiaceae]	g__Alishewanella
386	16	11	16	23	1	12	17	13	16	19	14	25	11	p__Actinobacteria	c__Actinobacteria	o__Actinomycetales	f__Microbacteriaceae	g__Agrococcus
387	6	6	23	67	1	4	5	2	5	20	11	31	6	p__Proteobacteria	c__Gammaproteobacte	o__Pseudomonadales	f__Moraxellaceae	g__Perlucidibaca
388	6	43	16	21	3	12	15	5	8	8	15	29	43	p__Actinobacteria	c__Rubrobacteria	o__Rubrobacterales	f__Rubrobacteraceae	g__
389	3	22	64	14	3	16	7	8	16	4	19	3	22	p__Proteobacteria	c__Betaproteobacteria	o__Burkholderiales	f__Burkholderiaceae	g__
390	2	37	11	9	19	6	8	14	16	2	26	28	37	p__Proteobacteria	c__Deltaproteobacteria	o__Bdellovibrionales	f__Bacteriovoracaceae	g__
391	15	18	8	35	3	3	11	5	18	49	6	7	18	p__Proteobacteria	c__Alphaproteobacteri	o__Rickettsiales	f__mitochondria	g__Sarcandra
392	21	19	47	17	1	1	25	5	13	6	6	7	19	p__Proteobacteria	c__Betaproteobacteria	o__Burkholderiales	f__Alcaligenaceae	g__
393	8	8	3	46	1	11	11	9	9	17	6	27	8	p__Proteobacteria	c__Alphaproteobacteri	o__Rickettsiales	f__mitochondria	g__Lupinus
394	7	3	15	8	1	5	2	10	16	18	29	39	3	p__Proteobacteria	c__Alphaproteobacteri	o__	f__	g__
395	3	3	6	34	1	9	5	2	23	44	12	11	3	p__Proteobacteria	c__Alphaproteobacteri	o__Rickettsiales	f__mitochondria	g__Carludovica
396	8	1	5	40	12	13	3	12	16	2	7	33	1	p__Proteobacteria	c__Betaproteobacteria	o__Burkholderiales	f__Comamonadaceae	g__Delftia
397	2	24	18	5	2	29	7	7	14	6	5	26	24	p__Bacteroidetes	c__[Rhodothermi]	o__[Rhodothermales]	f__Rhodothermaceae	g__Rubricoccus
398	8	1	4	57	3	6	13	6	11	23	5	7	1	p__Proteobacteria	c__Alphaproteobacteri	o__Rickettsiales	f__mitochondria	g__Citrullus
399	8	3	6	21	2	16	12	3	23	10	2	35	3	p__Actinobacteria	c__Actinobacteria	o__Actinomycetales	f__Actinosynnematace	g__Actinokineospora
400	38	5	12	26	5	6	10	2	2	2	19	12	5	p__Proteobacteria	c__Betaproteobacteria	o__Burkholderiales	f__Comamonadaceae	g__Rhodoferax
401	6	10	19	17	8	4	5	13	25	9	12	11	10	p__Proteobacteria	c__Gammaproteobacte	o__	f__	g__
402	12	19	5	27	3	8	4	4	17	1	21	10	19	p__Proteobacteria	c__Betaproteobacteria	o__Burkholderiales	f__Comamonadaceae	g__Limnohabitans
403	13	3	7	26	2	3	5	12	8	28	7	17	3	p__Proteobacteria	c__Alphaproteobacteri	o__Rickettsiales	f__mitochondria	g__Carica
404	3	21	15	13	3	6	19	3	13	7	16	6	21	p__Proteobacteria	c__Betaproteobacteria	o__ASSO-13	f__	g__
405	14	7	4	13	2	1	6	1	17	4	50	6	7	p__Proteobacteria	c__Betaproteobacteria	o__Burkholderiales	f__Alcaligenaceae	g__Achromobacter
406	1	17	2	2	2	1	4	1	19	1	32	36	17	p__Bacteroidetes	c__Flavobacteriia	o__Flavobacteriales	f__Flavobacteriaceae	g__Salinimicrobium
407	3	12	8	11	3	4	4	14	2	3	27	21	12	p__Proteobacteria	c__Alphaproteobacteri	o__Rhodospirillales	f__Acetobacteraceae	g__Roseococcus
408	10	4	15	6	3	2	17	10	5	12	5	21	4	p__Actinobacteria	c__Actinobacteria	o__Actinomycetales	f__Microbacteriaceae	g__Clavibacter
409	7	2	12	15	5	7	16	1	9	2	8	24	2	p__Actinobacteria	c__Actinobacteria	o__Actinomycetales	f__Promicromonospora	g__Luteimicrobium
410	7	1	9	1	1	10	22	7	17	3	10	19	1	p__Firmicutes	c__Clostridia	o__Clostridiales	f__Clostridiaceae	g__SMB53
411	5	3	16	9	3	7	15	5	11	13	5	15	3	p__Actinobacteria	c__Actinobacteria	o__Actinomycetales	f__Micrococcaceae	g__Renibacterium
412	3	9	2	14	1	3	4	2	18	4	6	36	9	p__Actinobacteria	c__Actinobacteria	o__Actinomycetales	f__Micromonosporacea	g__Pilimelia
413	3	9	8	13	2	6	13	5	8	11	10	8	9	p__Firmicutes	c__Bacilli	o__Bacillales	f__Planococcaceae	g__Paenisporosarcina
414	1	11	9	8	1	12	4	19	2	3	10	15	11	p__Actinobacteria	c__Actinobacteria	o__Actinomycetales	f__Nocardiopsaceae	g__
415	5	2	13	18	1	8	13	3	7	5	9	11	2	p__Actinobacteria	c__Actinobacteria	o__Actinomycetales	f__Intrasporangiaceae	g__Intrasporangium
416	7	9	10	13	1	4	9	7	14	4	2	9	9	p__Actinobacteria	c__Actinobacteria	o__Actinomycetales	f__Microbacteriaceae	g__Herbiconiux
417	1	1	6	2	2	1	40	1	7	1	1	5	1	p__Firmicutes	c__Bacilli	o__Bacillales	f__Planococcaceae	g__Viridibacillus
418	4	2	8	14	1	6	2	5	6	5	2	9	2	p__Actinobacteria	c__Actinobacteria	o__Actinomycetales	f__Pseudonocardiacea	g__Jiangella
419	5	2	3	8	3	2	9	2	9	6	4	2	2	p__Actinobacteria	c__Actinobacteria	o__Actinomycetales	f__Microbacteriaceae	g__Curtobacterium
420	2	2	3	11	4	5	4	2	4	1	2	8	2	p__Actinobacteria	c__Actinobacteria	o__Actinomycetales	f__Microbacteriaceae	g__Yonghaparkia

Table S5. Plant species identified using the mitochondrial ribosomal genes.

Chloroplast

S000323143	Calycanthus floridus var. glaucus (T); AJ428413
S000528956	Pinus thunbergii; D17510
S000529368	Cuscuta reflexa; X72584
S000529388	Zea mays; X86563
S000529451	Solanum nigrum; Y18934
S000531305	Cucumis sativus (T); AJ970307
S000531424	Physcomitrella patens subsp. patens (T); AP005672
S000575181	Eucalyptus globulus subsp. globulus; AY780259
S000609085	Lactuca sativa (T); AP007232
S000610747	Phalaenopsis aphrodite subsp. formosana; AY916449
S000626854	Glycine max (T); DQ317523
S000641069	Gossypium hirsutum (T); DQ345959
S000641682	Lycopersicon esculentum; AY216521
S000641743	Ricinus communis; L37580
S000641766	Sphagnum palustre; U24592
S000641768	Nicotiana tabacum; V00165
S000641769	Doodia maxima; U24583
S000641771	Juniperus virginiana; U24586
S000641773	Pisum sativum; cr. Progress No.9; pBX5; X51598
S000674963	Helianthus annuus (T); DQ383815
S000675135	Glechoma hederacea; DQ417652
S001020332	Buxus microphylla (T); EF380351
S001020365	Coffea arabica (T); EF044213
S001020373	Cuscuta exaltata (T); EU189132
S001020385	Cuscuta reflexa (T); AM711640
S001020389	Cycas taitungensis (T); AP009339
S001020393	Daucus carota (T); DQ898156
S001020397	Dioscorea elephantipes (T); EF380353
S001020405	Drimys granadensis (T); DQ887676
S001020421	Ipomoea purpurea (T); EU118126
S001020439	Liriodendron tulipifera (T); DQ899947
S001020496	Phaseolus vulgaris (T); DQ886273
S001020502	Piper cenocladum (T); DQ887677
S001020510	Populus alba (T); AP008956

Mitochondria

k__Bacteria	p__Proteobacteria	c__Alphaproteobacteria	o__Rickettsiales	f__mitochondria	g__Abies	s__homolepis
k__Bacteria	p__Proteobacteria	c__Alphaproteobacteria	o__Rickettsiales	f__mitochondria	g__Anomodon	s__rugelii
k__Bacteria	p__Proteobacteria	c__Alphaproteobacteria	o__Rickettsiales	f__mitochondria	g__Arabidopsis	s__thaliana
k__Bacteria	p__Proteobacteria	c__Alphaproteobacteria	o__Rickettsiales	f__mitochondria	g__Aristolochia	s__macrophylla
k__Bacteria	p__Proteobacteria	c__Alphaproteobacteria	o__Rickettsiales	f__mitochondria	g__Asarum	s__canadense
k__Bacteria	p__Proteobacteria	c__Alphaproteobacteria	o__Rickettsiales	f__mitochondria	g__Azolla	s__pinnata
k__Bacteria	p__Proteobacteria	c__Alphaproteobacteria	o__Rickettsiales	f__mitochondria	g__Calycanthus	s__floridus
k__Bacteria	p__Proteobacteria	c__Alphaproteobacteria	o__Rickettsiales	f__mitochondria	g__Carica	s__papaya
k__Bacteria	p__Proteobacteria	c__Alphaproteobacteria	o__Rickettsiales	f__mitochondria	g__Carludovica	s__palmata
k__Bacteria	p__Proteobacteria	c__Alphaproteobacteria	o__Rickettsiales	f__mitochondria	g__Citrullus	s__lanatus
k__Bacteria	p__Proteobacteria	c__Alphaproteobacteria	o__Rickettsiales	f__mitochondria	g__Didymeles	s__perrieri
k__Bacteria	p__Proteobacteria	c__Alphaproteobacteria	o__Rickettsiales	f__mitochondria	g__Diplazium	s__pycnocarpon
k__Bacteria	p__Proteobacteria	c__Alphaproteobacteria	o__Rickettsiales	f__mitochondria	g__Euptelea	s__polyandra
k__Bacteria	p__Proteobacteria	c__Alphaproteobacteria	o__Rickettsiales	f__mitochondria	g__Galbulimima	s__belgraveana
k__Bacteria	p__Proteobacteria	c__Alphaproteobacteria	o__Rickettsiales	f__mitochondria	g__Grevillea	s__robusta
k__Bacteria	p__Proteobacteria	c__Alphaproteobacteria	o__Rickettsiales	f__mitochondria	g__Gyrocarpus	s__americanus
k__Bacteria	p__Proteobacteria	c__Alphaproteobacteria	o__Rickettsiales	f__mitochondria	g__Hypseocharis	s__pimpinellifolia
k__Bacteria	p__Proteobacteria	c__Alphaproteobacteria	o__Rickettsiales	f__mitochondria	g__Lupinus	s__luteus
k__Bacteria	p__Proteobacteria	c__Alphaproteobacteria	o__Rickettsiales	f__mitochondria	g__Nageia	s__nagi
k__Bacteria	p__Proteobacteria	c__Alphaproteobacteria	o__Rickettsiales	f__mitochondria	g__Nelumbo	s__nucifera
k__Bacteria	p__Proteobacteria	c__Alphaproteobacteria	o__Rickettsiales	f__mitochondria	g__Oenothera	s__berteroana
k__Bacteria	p__Proteobacteria	c__Alphaproteobacteria	o__Rickettsiales	f__mitochondria	g__Phaeoceros	s__laevis
k__Bacteria	p__Proteobacteria	c__Alphaproteobacteria	o__Rickettsiales	f__mitochondria	g__Plantago	s__sericea
k__Bacteria	p__Proteobacteria	c__Alphaproteobacteria	o__Rickettsiales	f__mitochondria	g__Pleurozia	s__purpurea
k__Bacteria	p__Proteobacteria	c__Alphaproteobacteria	o__Rickettsiales	f__mitochondria	g__Raphanus	s__sativus
k__Bacteria	p__Proteobacteria	c__Alphaproteobacteria	o__Rickettsiales	f__mitochondria	g__	s__
k__Bacteria	p__Proteobacteria	c__Alphaproteobacteria	o__Rickettsiales	f__mitochondria	g__Sarcandra	s__grandifolia
k__Bacteria	p__Proteobacteria	c__Alphaproteobacteria	o__Rickettsiales	f__mitochondria	g__Syntrichia	s__ruralis
k__Bacteria	p__Proteobacteria	c__Alphaproteobacteria	o__Rickettsiales	f__mitochondria	g__Zea	s__luxurians

Table S6. OTUs with differential abundances between trains and stations (p < 1e-5).

Enriched OTUs in stations

OTU	log2Fold Change	*pvalue*	*padj*	Phylum	Class	Order	Family	Genus
411738	5.35	2.3E-12	5.7E-08	p__Actinobacteria	c__Actinobacteria	o__Actinomycetales	f__Micrococcaceae	g__
7460	5.03	7.2E-11	1.2E-06	p__Firmicutes	c__Bacilli	o__Lactobacillales	f__Carnobacteriaceae	g__
449580	4.61	2.4E-09	1.1E-05	p__Actinobacteria	c__Actinobacteria	o__Actinomycetales	f__Kineosporiaceae	g__Kineococcus
375356	4.45	2.8E-09	1.1E-05	p__Bacteroidetes	c__Cytophagia	o__Cytophagales	f__Cytophagaceae	g__Adhaeribacter
501268	4.33	4.1E-06	1.7E-03	p__Firmicutes	c__Clostridia	o__Clostridiales	f__	g__
433442	4.11	2.0E-05	5.4E-03	p__Actinobacteria	c__Actinobacteria	o__Actinomycetales	f__Nocardioidaceae	g__
406101	3.96	2.1E-08	4.4E-05	p__Actinobacteria	c__Actinobacteria	o__Actinomycetales	f__Actinomycetaceae	g__Actinomyces
417557	3.86	1.6E-07	1.8E-04	p__Gemmatimonadetes	c__Gemm-1	o__	f__	g__
438980	3.84	1.2E-12	5.7E-08	p__Actinobacteria	c__Actinobacteria	o__Actinomycetales	f__Micrococcaceae	g__Kocuria
610982	3.70	3.6E-05	8.2E-03	p__Firmicutes	c__Clostridia	o__Clostridiales	f__Lachnospiraceae	g__Shuttleworthia
436120	3.57	1.4E-06	8.5E-04	p__Actinobacteria	c__Actinobacteria	o__Actinomycetales	f__Micromonosporaceae	g__Micromonospora
668556	3.36	2.2E-05	5.7E-03	p__Actinobacteria	c__Actinobacteria	o__Actinomycetales	f__Geodermatophilaceae	g__Blastococcus
396691	3.29	1.4E-05	4.1E-03	p__Actinobacteria	c__Actinobacteria	o__Actinomycetales	f__Pseudonocardiaceae	g__Actinomycetospora
428412	3.28	9.8E-07	6.5E-04	p__Actinobacteria	c__Actinobacteria	o__Actinomycetales	f__Geodermatophilaceae	g__Geodermatophilus
364075	3.23	2.4E-05	6.0E-03	p__Bacteroidetes	c__Flavobacteriia	o__Flavobacteriales	f__Flavobacteriaceae	g__Flavobacterium
410344	3.23	3.0E-06	1.4E-03	p__Actinobacteria	c__Actinobacteria	o__Actinomycetales	f__Micrococcaceae	g__Arthrobacter
612064	3.20	6.6E-06	2.4E-03	p__Proteobacteria	c__Alphaproteobacteria	o__Rhodobacterales	f__Rhodobacteraceae	g__Paracoccus
438435	3.15	1.7E-06	9.7E-04	p__Actinobacteria	c__Actinobacteria	o__Actinomycetales	f__Micrococcaceae	g__
405607	3.09	3.0E-05	7.2E-03	p__Actinobacteria	c__Actinobacteria	o__Actinomycetales	f__Actinomycetaceae	g__
610498	3.07	4.2E-05	9.1E-03	p__Firmicutes	c__Clostridia	o__Clostridiales	f__Lachnospiraceae	g__Blautia
427945	3.05	5.6E-07	4.3E-04	p__Actinobacteria	c__Actinobacteria	o__Actinomycetales	f__Geodermatophilaceae	g__Geodermatophilus
410694	3.05	4.0E-06	1.7E-03	p__Actinobacteria	c__Actinobacteria	o__Actinomycetales	f__Intrasporangiaceae	g__
577669	3.01	1.0E-09	5.6E-06	p__Cyanobacteria	c__Chloroplast	o__Streptophyta	f__	g__
586265	3.00	4.0E-05	8.7E-03	p__Chloroflexi	c__Thermomicrobia	o__JG30-KF-CM45	f__	g__
401598	2.89	1.1E-05	3.6E-03	p__Actinobacteria	c__Actinobacteria	o__Actinomycetales	f__Micrococcaceae	g__
426514	2.88	2.5E-08	5.0E-05	p__Actinobacteria	c__Actinobacteria	o__Actinomycetales	f__Dermatophilaceae	g__Kineosphaera
418815	2.85	2.2E-05	5.8E-03	p__[Thermi]	c__Deinococci	o__Deinococcales	f__Deinococcaceae	g__Deinococcus
7206	2.83	8.2E-08	1.1E-04	p__Firmicutes	c__Bacilli	o__Lactobacillales	f__Streptococcaceae	g__Lactococcus
416395	2.81	2.8E-08	5.1E-05	p__Actinobacteria	c__Actinobacteria	o__Actinomycetales	f__Micrococcaceae	g__Kocuria
437788	2.81	3.3E-07	2.9E-04	p__Actinobacteria	c__Actinobacteria	o__Actinomycetales	f__Geodermatophilaceae	g__Geodermatophilus
452557	2.79	4.2E-05	9.1E-03	p__Proteobacteria	c__Gammaproteobacteria	o__Pseudomonadales	f__Pseudomonadaceae	g__
420367	2.74	5.5E-06	2.1E-03	p__Actinobacteria	c__Actinobacteria	o__Actinomycetales	f__	g__
448521	2.74	1.2E-05	3.8E-03	p__Actinobacteria	c__Actinobacteria	o__Actinomycetales	f__	g__
658	2.73	2.4E-05	6.0E-03	p__Firmicutes	c__Bacilli	o__Lactobacillales	f__Streptococcaceae	g__Lactococcus
451158	2.73	1.5E-05	4.5E-03	p__Actinobacteria	c__Actinobacteria	o__Actinomycetales	f__Intrasporangiaceae	g__Terracoccus
611728	2.73	3.8E-05	8.7E-03	p__Proteobacteria	c__Gammaproteobacteria	o__Pseudomonadales	f__Pseudomonadaceae	g__
453036	2.70	2.4E-05	6.1E-03	p__Actinobacteria	c__Actinobacteria	o__Actinomycetales	f__Nocardioidaceae	g__
597451	2.65	2.2E-06	1.2E-03	p__Actinobacteria	c__Acidimicrobiia	o__Acidimicrobiales	f__	g__
9657	2.62	3.1E-08	5.3E-05	p__Firmicutes	c__Bacilli	o__Bacillales	f__Paenibacillaceae	g__
602533	2.60	2.8E-05	6.8E-03	p__Proteobacteria	c__Alphaproteobacteria	o__Rhodospirillales	f__Acetobacteraceae	g__
418679	2.57	2.1E-05	5.6E-03	p__Actinobacteria	c__Actinobacteria	o__Actinomycetales	f__Nocardioidaceae	g__Nocardioides
451608	2.55	2.6E-05	6.5E-03	p__Actinobacteria	c__Actinobacteria	o__Actinomycetales	f__Dermatophilaceae	g__Kineosphaera
438886	2.50	8.9E-06	3.0E-03	p__Actinobacteria	c__Actinobacteria	o__Actinomycetales	f__Micrococcaceae	g__Arthrobacter
419138	2.48	5.0E-06	2.0E-03	p__Actinobacteria	c__Actinobacteria	o__Actinomycetales	f__Nocardioidaceae	g__
412066	2.47	3.6E-09	1.3E-05	p__Actinobacteria	c__Actinobacteria	o__Actinomycetales	f__	g__
415729	2.46	4.5E-05	9.5E-03	p__Actinobacteria	c__Actinobacteria	o__Actinomycetales	f__Micrococcaceae	g__Kocuria
610499	2.41	2.4E-05	6.0E-03	p__Firmicutes	c__Clostridia	o__Clostridiales	f__Lachnospiraceae	g__
410539	2.38	2.4E-06	1.2E-03	p__Actinobacteria	c__Actinobacteria	o__Actinomycetales	f__Micrococcaceae	g__
446671	2.38	3.2E-07	2.9E-04	p__Actinobacteria	c__Actinobacteria	o__Actinomycetales	f__Dermatophilaceae	g__Piscicoccus
409978	2.34	2.3E-07	2.4E-04	p__Actinobacteria	c__Actinobacteria	o__Actinomycetales	f__Brevibacteriaceae	g__Brevibacterium
438316	2.31	3.3E-05	7.7E-03	p__Actinobacteria	c__Actinobacteria	o__Actinomycetales	f__Geodermatophilaceae	g__Geodermatophilus
417344	2.29	2.4E-07	2.4E-04	p__Actinobacteria	c__Actinobacteria	o__Actinomycetales	f__Micrococcaceae	g__Kocuria
407597	2.29	1.1E-05	3.5E-03	p__Actinobacteria	c__Actinobacteria	o__Actinomycetales	f__Nocardioidaceae	g__
429245	2.28	2.9E-06	1.4E-03	p__Actinobacteria	c__Actinobacteria	o__Actinomycetales	f__	g__
609729	2.26	6.1E-07	4.6E-04	p__Proteobacteria	c__Alphaproteobacteria	o__Rhodospirillales	f__Rhodospirillaceae	g__Skermanella
428144	2.24	5.3E-09	1.6E-05	p__Actinobacteria	c__Actinobacteria	o__Actinomycetales	f__Micrococcaceae	g__Arthrobacter
416482	2.17	4.3E-05	9.2E-03	p__Actinobacteria	c__Actinobacteria	o__Actinomycetales	f__Micrococcaceae	g__
5768	2.13	2.7E-05	6.7E-03	p__Firmicutes	c__Bacilli	o__Lactobacillales	f__Leuconostocaceae	g__Leuconostoc
453381	2.12	4.4E-05	9.3E-03	p__Actinobacteria	c__Actinobacteria	o__Actinomycetales	f__Geodermatophilaceae	g__Geodermatophilus
415571	2.11	4.6E-08	7.1E-05	p__Actinobacteria	c__Actinobacteria	o__Actinomycetales	f__Nocardioidaceae	g__
375862	2.07	4.5E-06	1.9E-03	p__Actinobacteria	c__Actinobacteria	o__Actinomycetales	f__Micrococcaceae	g__
441781	2.06	9.4E-06	3.1E-03	p__Actinobacteria	c__Actinobacteria	o__Actinomycetales	f__Micrococcaceae	g__Kocuria
428699	2.06	6.0E-06	2.2E-03	p__Actinobacteria	c__Actinobacteria	o__Actinomycetales	f__Micrococcaceae	g__
437408	2.04	2.7E-08	5.1E-05	p__Actinobacteria	c__Actinobacteria	o__Actinomycetales	f__Micrococcaceae	g__Kocuria
536	2.04	5.3E-06	2.0E-03	p__Firmicutes	c__Bacilli	o__Lactobacillales	f__Streptococcaceae	g__Lactococcus
424791	2.04	5.8E-08	8.6E-05	p__Actinobacteria	c__Actinobacteria	o__Actinomycetales	f__Micrococcaceae	g__Kocuria
426510	2.03	2.5E-05	6.3E-03	p__Cyanobacteria	c__Chloroplast	o__Streptophyta	f__	g__
434933	2.02	6.2E-06	2.3E-03	p__Actinobacteria	c__Actinobacteria	o__Actinomycetales	f__Cellulomonadaceae	g__Actinotalea
435669	2.01	5.4E-06	2.1E-03	p__Actinobacteria	c__Actinobacteria	o__Actinomycetales	f__Micrococcaceae	g__Kocuria
397576	2.00	7.2E-06	2.6E-03	p__Actinobacteria	c__Actinobacteria	o__Actinomycetales	f__Beutenbergiaceae	g__Beutenbergia
438390	1.99	1.1E-06	7.2E-04	p__Actinobacteria	c__Actinobacteria	o__Actinomycetales	f__Micrococcaceae	g__
581015	1.98	1.8E-06	1.0E-03	p__Chloroflexi	c__Thermomicrobia	o__JG30-KF-CM45	f__	g__
403428	1.97	1.1E-05	3.5E-03	p__Actinobacteria	c__Actinobacteria	o__Actinomycetales	f__Corynebacteriaceae	g__Corynebacterium
412109	1.97	1.3E-05	4.1E-03	p__Actinobacteria	c__Actinobacteria	o__Actinomycetales	f__Micrococcaceae	g__
421078	1.96	4.8E-07	3.9E-04	p__Actinobacteria	c__Actinobacteria	o__Actinomycetales	f__Micrococcaceae	g__Kocuria
446883	1.96	4.8E-06	1.9E-03	p__Actinobacteria	c__Actinobacteria	o__Actinomycetales	f__	g__
436034	1.95	1.7E-06	9.7E-04	p__Actinobacteria	c__Actinobacteria	o__Actinomycetales	f__Geodermatophilaceae	g__Modestobacter
420067	1.94	4.9E-06	2.0E-03	p__Actinobacteria	c__Actinobacteria	o__Actinomycetales	f__Nocardioidaceae	g__
447794	1.94	3.4E-06	1.6E-03	p__Actinobacteria	c__Actinobacteria	o__Actinomycetales	f__Nocardioidaceae	g__

449670	1.92	4.4E-05	9.3E-03	p__Actinobacteria	c__Actinobacteria	o__Actinomycetales	f__Micrococcaceae	g__
423219	1.85	1.9E-05	5.3E-03	p__Actinobacteria	c__Actinobacteria	o__Actinomycetales	f__Intrasporangiaceae	g__Serinicoccus
449755	1.84	3.0E-06	1.4E-03	p__Actinobacteria	c__Actinobacteria	o__Actinomycetales	f__Propionibacteriaceae	g__Tessaracoccus
435756	1.82	3.6E-06	1.6E-03	p__Actinobacteria	c__Actinobacteria	o__Actinomycetales	f__Micrococcaceae	g__Micrococcus
408004	1.82	1.7E-05	4.9E-03	p__Actinobacteria	c__Actinobacteria	o__Actinomycetales	f__Cellulomonadaceae	g__
429560	1.82	1.4E-05	4.2E-03	p__Actinobacteria	c__Actinobacteria	o__Actinomycetales	f__Micrococcaceae	g__Kocuria
439201	1.81	3.9E-05	8.7E-03	p__Actinobacteria	c__Actinobacteria	o__Actinomycetales	f__Dietziaceae	g__Dietzia
426384	1.81	7.0E-08	9.9E-05	p__Actinobacteria	c__Actinobacteria	o__Actinomycetales	f__Intrasporangiaceae	g__Serinicoccus
435228	1.78	4.0E-05	8.7E-03	p__Actinobacteria	c__Actinobacteria	o__Actinomycetales	f__Dermabacteraceae	g__Brachybacterium
428009	1.77	2.4E-05	6.0E-03	p__Actinobacteria	c__Actinobacteria	o__Actinomycetales	f__Corynebacteriaceae	g__Corynebacterium
398075	1.77	3.7E-06	1.6E-03	p__Actinobacteria	c__Actinobacteria	o__Actinomycetales	f__Micrococcaceae	g__
448725	1.77	3.8E-05	8.7E-03	p__Actinobacteria	c__Actinobacteria	o__Actinomycetales	f__Sanguibacteraceae	g__Sanguibacter
415588	1.76	4.0E-06	1.7E-03	p__Actinobacteria	c__Actinobacteria	o__Actinomycetales	f__Geodermatophilaceae	g__Blastococcus
426580	1.75	8.9E-06	3.0E-03	p__Actinobacteria	c__Actinobacteria	o__Actinomycetales	f__Micrococcaceae	g__
448932	1.73	4.6E-05	9.5E-03	p__Actinobacteria	c__Actinobacteria	o__Actinomycetales	f__Propionibacteriaceae	g__Luteococcus
410839	1.72	1.2E-06	7.3E-04	p__Actinobacteria	c__Actinobacteria	o__Actinomycetales	f__Geodermatophilaceae	g__Blastococcus
407509	1.72	3.8E-05	8.7E-03	p__Actinobacteria	c__Actinobacteria	o__Actinomycetales	f__Cellulomonadaceae	g__
432470	1.70	3.0E-07	2.9E-04	p__Actinobacteria	c__Actinobacteria	o__Actinomycetales	f__Intrasporangiaceae	g__
447448	1.69	9.7E-08	1.3E-04	p__Actinobacteria	c__Actinobacteria	o__Actinomycetales	f__Nocardioidaceae	g__
407659	1.69	3.4E-07	2.9E-04	p__Actinobacteria	c__Actinobacteria	o__Actinomycetales	f__Micrococcaceae	g__
426344	1.68	1.8E-05	5.1E-03	p__Actinobacteria	c__Actinobacteria	o__Actinomycetales	f__Micrococcaceae	g__
433066	1.67	2.7E-06	1.3E-03	p__Actinobacteria	c__Actinobacteria	o__Actinomycetales	f__Geodermatophilaceae	g__Blastococcus
447895	1.65	2.1E-06	1.2E-03	p__Actinobacteria	c__Actinobacteria	o__Actinomycetales	f__Microbacteriaceae	g__Microbacterium
405577	1.63	4.7E-06	1.9E-03	p__Actinobacteria	c__Actinobacteria	o__Actinomycetales	f__Micrococcaceae	g__Arthrobacter
420948	1.63	1.4E-08	3.3E-05	p__Actinobacteria	c__Actinobacteria	o__Actinomycetales	f__Micrococcaceae	g__
447574	1.63	5.4E-06	2.1E-03	p__Actinobacteria	c__Actinobacteria	o__Actinomycetales	f__Dietziaceae	g__Dietzia
422866	1.63	3.9E-07	3.3E-04	p__Actinobacteria	c__Actinobacteria	o__Actinomycetales	f__Micrococcaceae	g__Kocuria
405937	1.61	3.0E-08	5.3E-05	p__Actinobacteria	c__Actinobacteria	o__Actinomycetales	f__Micrococcaceae	g__
383627	1.60	1.2E-07	1.5E-04	p__Actinobacteria	c__Actinobacteria	o__Actinomycetales	f__Micrococcaceae	g__Kocuria
14623	1.60	3.3E-05	7.8E-03	p__Firmicutes	c__Bacilli	o__Lactobacillales	f__Carnobacteriaceae	g__Desemzia
412443	1.59	4.1E-05	8.9E-03	p__Actinobacteria	c__Actinobacteria	o__Actinomycetales	f__	g__
411771	1.59	8.5E-07	5.8E-04	p__Actinobacteria	c__Actinobacteria	o__Actinomycetales	f__Micrococcaceae	g__
436075	1.58	1.0E-05	3.4E-03	p__Actinobacteria	c__Actinobacteria	o__Actinomycetales	f__Cellulomonadaceae	g__Cellulomonas
433432	1.54	8.6E-07	5.8E-04	p__Actinobacteria	c__Actinobacteria	o__Actinomycetales	f__Micrococcaceae	g__Kocuria
415433	1.53	2.8E-06	1.4E-03	p__Actinobacteria	c__Actinobacteria	o__Actinomycetales	f__Micrococcaceae	g__
426399	1.50	3.1E-05	7.5E-03	p__Actinobacteria	c__Actinobacteria	o__Actinomycetales	f__Dermabacteraceae	g__Brachybacterium
436574	1.48	9.4E-06	3.1E-03	p__Actinobacteria	c__Actinobacteria	o__Actinomycetales	f__Geodermatophilaceae	g__Blastococcus
7642	1.47	3.0E-06	1.4E-03	p__Firmicutes	c__Bacilli	o__Lactobacillales	f__Aerococcaceae	g__Aerococcus
437725	1.45	1.1E-06	7.2E-04	p__Actinobacteria	c__Actinobacteria	o__Actinomycetales	f__Geodermatophilaceae	g__Blastococcus
448056	1.44	2.0E-05	5.4E-03	p__Actinobacteria	c__Actinobacteria	o__Actinomycetales	f__Micrococcaceae	g__
6544	1.43	1.1E-05	3.6E-03	p__Firmicutes	c__Bacilli	o__Lactobacillales	f__Aerococcaceae	g__Aerococcus
411341	1.43	4.6E-05	9.5E-03	p__Actinobacteria	c__Actinobacteria	o__Actinomycetales	f__Micrococcaceae	g__
407471	1.38	5.9E-08	8.6E-05	p__Actinobacteria	c__Actinobacteria	o__Actinomycetales	f__	g__
401629	1.37	1.3E-06	7.9E-04	p__Actinobacteria	c__Actinobacteria	o__Actinomycetales	f__Cellulomonadaceae	g__
435316	1.33	4.8E-05	9.9E-03	p__Actinobacteria	c__Actinobacteria	o__Actinomycetales	f__Micrococcaceae	g__Kocuria
610318	1.29	3.4E-05	8.0E-03	p__Proteobacteria	c__Alphaproteobacteria	o__Rhodobacterales	f__Rhodobacteraceae	g__Paracoccus
436581	1.26	6.4E-06	2.3E-03	p__Actinobacteria	c__Actinobacteria	o__Actinomycetales	f__Micrococcaceae	g__Microbispora
436818	1.26	4.4E-05	9.3E-03	p__Actinobacteria	c__Actinobacteria	o__Actinomycetales	f__Micrococcaceae	g__Micrococcus
397705	1.20	4.4E-05	9.3E-03	p__Actinobacteria	c__Actinobacteria	o__Actinomycetales	f__Micrococcaceae	g__
13672	1.16	4.5E-05	9.4E-03	p__Firmicutes	c__Bacilli	o__Lactobacillales	f__Aerococcaceae	g__Aerococcus
426782	1.11	2.7E-05	6.6E-03	p__Actinobacteria	c__Actinobacteria	o__Actinomycetales	f__Micrococcaceae	g__
412566	1.11	6.3E-06	2.3E-03	p__Actinobacteria	c__Actinobacteria	o__Actinomycetales	f__Micrococcaceae	g__

Enriched OTUs in trains

OTU	log2Fold Change	*pvalue*	*padj*	Phylum	Class	Order	Family	Genus
447246	-1.12	1.4E-05	4.3E-03	p__Actinobacteria	c__Actinobacteria	o__Actinomycetales	f__Propionibacteriaceae	g__Propionibacterium
446431	-1.27	3.5E-05	8.0E-03	p__Actinobacteria	c__Actinobacteria	o__Actinomycetales	f__Propionibacteriaceae	g__Propionibacterium
425846	-1.28	4.9E-07	3.9E-04	p__Actinobacteria	c__Actinobacteria	o__Actinomycetales	f__Propionibacteriaceae	g__Propionibacterium
400244	-1.33	1.0E-06	6.7E-04	p__Actinobacteria	c__Actinobacteria	o__Actinomycetales	f__Propionibacteriaceae	g__Propionibacterium
435908	-1.34	4.6E-05	9.5E-03	p__Actinobacteria	c__Actinobacteria	o__Actinomycetales	f__Corynebacteriaceae	g__Corynebacterium
414180	-1.34	5.2E-07	4.1E-04	p__Actinobacteria	c__Actinobacteria	o__Actinomycetales	f__Propionibacteriaceae	g__Propionibacterium
416710	-1.34	4.1E-05	8.9E-03	p__Actinobacteria	c__Actinobacteria	o__Actinomycetales	f__Corynebacteriaceae	g__Corynebacterium
448607	-1.36	6.5E-07	4.7E-04	p__Actinobacteria	c__Actinobacteria	o__Actinomycetales	f__Propionibacteriaceae	g__Propionibacterium
434671	-1.37	2.5E-05	6.3E-03	p__Actinobacteria	c__Actinobacteria	o__Actinomycetales	f__Corynebacteriaceae	g__Corynebacterium
433788	-1.37	1.4E-07	1.6E-04	p__Actinobacteria	c__Actinobacteria	o__Actinomycetales	f__Propionibacteriaceae	g__Propionibacterium
421710	-1.38	7.8E-08	1.1E-04	p__Actinobacteria	c__Actinobacteria	o__Actinomycetales	f__Propionibacteriaceae	g__Propionibacterium
435367	-1.39	4.3E-06	1.8E-03	p__Actinobacteria	c__Actinobacteria	o__Actinomycetales	f__Corynebacteriaceae	g__Corynebacterium
443776	-1.44	3.6E-06	1.6E-03	p__Actinobacteria	c__Actinobacteria	o__Actinomycetales	f__Corynebacteriaceae	g__Corynebacterium
451969	-1.45	4.7E-06	1.9E-03	p__Actinobacteria	c__Actinobacteria	o__Actinomycetales	f__Propionibacteriaceae	g__Propionibacterium
453162	-1.46	2.1E-05	5.5E-03	p__Actinobacteria	c__Actinobacteria	o__Actinomycetales	f__Corynebacteriaceae	g__Corynebacterium
415663	-1.49	2.8E-05	6.7E-03	p__Actinobacteria	c__Actinobacteria	o__Actinomycetales	f__Propionibacteriaceae	g__Propionibacterium
433784	-1.49	3.3E-07	2.9E-04	p__Actinobacteria	c__Actinobacteria	o__Actinomycetales	f__Propionibacteriaceae	g__Propionibacterium
417311	-1.57	2.0E-05	5.4E-03	p__Actinobacteria	c__Actinobacteria	o__Actinomycetales	f__Corynebacteriaceae	g__Corynebacterium
434807	-1.63	3.2E-05	7.5E-03	p__Actinobacteria	c__Actinobacteria	o__Actinomycetales	f__Pseudonocardiaceae	g__
444262	-1.63	1.6E-05	4.7E-03	p__Actinobacteria	c__Actinobacteria	o__Actinomycetales	f__Propionibacteriaceae	g__Propionibacterium
434203	-1.65	1.6E-06	9.4E-04	p__Actinobacteria	c__Actinobacteria	o__Actinomycetales	f__Propionibacteriaceae	g__Propionibacterium
464862	-1.66	7.6E-07	5.4E-04	p__Actinobacteria	c__Actinobacteria	o__Actinomycetales	f__Propionibacteriaceae	g__Propionibacterium
426909	-1.67	3.7E-06	1.6E-03	p__Actinobacteria	c__Actinobacteria	o__Actinomycetales	f__Propionibacteriaceae	g__Propionibacterium
403857	-1.67	1.9E-05	5.2E-03	p__Actinobacteria	c__Actinobacteria	o__Actinomycetales	f__Propionibacteriaceae	g__Propionibacterium
425788	-1.68	2.9E-05	7.0E-03	p__Actinobacteria	c__Actinobacteria	o__Actinomycetales	f__Propionibacteriaceae	g__Propionibacterium
418259	-1.68	3.0E-06	1.4E-03	p__Actinobacteria	c__Actinobacteria	o__Actinomycetales	f__Propionibacteriaceae	g__Propionibacterium
435213	-1.69	1.9E-05	5.3E-03	p__Actinobacteria	c__Actinobacteria	o__Actinomycetales	f__Corynebacteriaceae	g__Corynebacterium
423569	-1.70	2.6E-06	1.3E-03	p__Actinobacteria	c__Actinobacteria	o__Actinomycetales	f__Corynebacteriaceae	g__Corynebacterium
436913	-1.73	3.9E-05	8.7E-03	p__Actinobacteria	c__Actinobacteria	o__Actinomycetales	f__Corynebacteriaceae	g__Corynebacterium
407339	-1.75	8.5E-06	2.9E-03	p__Actinobacteria	c__Actinobacteria	o__Actinomycetales	f__Propionibacteriaceae	g__Propionibacterium

452188	-1.75	1.8E-05	5.1E-03	p__Actinobacteria	c__Actinobacteria	o__Actinomycetales	f__Propionibacteriaceae	g__Propionibacterium
416612	-1.76	1.7E-05	4.9E-03	p__Actinobacteria	c__Actinobacteria	o__Actinomycetales	f__Corynebacteriaceae	g__Corynebacterium
435523	-1.76	4.5E-07	3.7E-04	p__Actinobacteria	c__Actinobacteria	o__Actinomycetales	f__Corynebacteriaceae	g__Corynebacterium
458515	-1.76	2.3E-07	2.4E-04	p__Actinobacteria	c__Actinobacteria	o__Actinomycetales	f__Corynebacteriaceae	g__Corynebacterium
433156	-1.76	8.1E-07	5.7E-04	p__Actinobacteria	c__Actinobacteria	o__Actinomycetales	f__Corynebacteriaceae	g__Corynebacterium
461651	-1.76	2.2E-05	5.8E-03	p__Actinobacteria	c__Actinobacteria	o__Actinomycetales	f__Propionibacteriaceae	g__Propionibacterium
430847	-1.78	8.8E-06	3.0E-03	p__Actinobacteria	c__Actinobacteria	o__Actinomycetales	f__Propionibacteriaceae	g__Propionibacterium
458400	-1.79	1.8E-05	5.1E-03	p__Actinobacteria	c__Actinobacteria	o__Actinomycetales	f__Propionibacteriaceae	g__Propionibacterium
403328	-1.80	1.7E-05	5.0E-03	p__Actinobacteria	c__Actinobacteria	o__Actinomycetales	f__Corynebacteriaceae	g__Corynebacterium
399319	-1.80	5.3E-07	4.1E-04	p__Actinobacteria	c__Actinobacteria	o__Actinomycetales	f__Propionibacteriaceae	g__Propionibacterium
434390	-1.82	7.8E-06	2.7E-03	p__Actinobacteria	c__Actinobacteria	o__Actinomycetales	f__Corynebacteriaceae	g__Corynebacterium
410753	-1.86	7.5E-10	4.7E-06	p__Actinobacteria	c__Actinobacteria	o__Actinomycetales	f__Corynebacteriaceae	g__Corynebacterium
435535	-1.86	1.7E-05	5.0E-03	p__Actinobacteria	c__Actinobacteria	o__Actinomycetales	f__Corynebacteriaceae	g__Corynebacterium
389672	-1.88	3.2E-06	1.5E-03	p__Actinobacteria	c__Actinobacteria	o__Actinomycetales	f__Corynebacteriaceae	g__Corynebacterium
419191	-1.90	7.2E-06	2.6E-03	p__Firmicutes	c__Clostridia	o__Clostridiales	f__Veillonellaceae	g__Veillonella
350172	-1.91	1.4E-06	8.4E-04	p__Actinobacteria	c__Actinobacteria	o__Actinomycetales	f__Corynebacteriaceae	g__Corynebacterium
441990	-1.91	2.1E-05	5.6E-03	p__Actinobacteria	c__Actinobacteria	o__Actinomycetales	f__Corynebacteriaceae	g__Corynebacterium
431043	-1.92	3.0E-05	7.1E-03	p__Actinobacteria	c__Actinobacteria	o__Actinomycetales	f__Corynebacteriaceae	g__Corynebacterium
611945	-1.93	1.7E-05	5.0E-03	p__Actinobacteria	c__Actinobacteria	o__Actinomycetales	f__Corynebacteriaceae	g__Corynebacterium
436102	-1.94	1.6E-06	9.2E-04	p__Actinobacteria	c__Actinobacteria	o__Actinomycetales	f__Corynebacteriaceae	g__Corynebacterium
26049	-1.94	2.2E-06	1.2E-03	p__Actinobacteria	c__Actinobacteria	o__Actinomycetales	f__Corynebacteriaceae	g__Corynebacterium
334952	-1.94	2.4E-06	1.2E-03	p__Actinobacteria	c__Actinobacteria	o__Actinomycetales	f__Propionibacteriaceae	g__Propionibacterium
420450	-1.95	1.4E-05	4.2E-03	p__Actinobacteria	c__Actinobacteria	o__Actinomycetales	f__Propionibacteriaceae	g__Propionibacterium
425500	-1.95	2.3E-07	2.4E-04	p__Actinobacteria	c__Actinobacteria	o__Actinomycetales	f__Corynebacteriaceae	g__Corynebacterium
395460	-1.96	1.7E-06	9.7E-04	p__Actinobacteria	c__Actinobacteria	o__Actinomycetales	f__Corynebacteriaceae	g__Corynebacterium
424449	-1.96	4.6E-06	1.9E-03	p__Actinobacteria	c__Actinobacteria	o__Actinomycetales	f__Corynebacteriaceae	g__Corynebacterium
446158	-1.98	4.6E-05	9.5E-03	p__Actinobacteria	c__Actinobacteria	o__Actinomycetales	f__Corynebacteriaceae	g__Corynebacterium
431344	-1.98	7.5E-06	2.6E-03	p__Actinobacteria	c__Actinobacteria	o__Actinomycetales	f__Propionibacteriaceae	g__Propionibacterium
434996	-2.01	1.3E-05	3.9E-03	p__Actinobacteria	c__Actinobacteria	o__Actinomycetales	f__Propionibacteriaceae	g__Propionibacterium
466644	-2.02	5.6E-10	4.7E-06	p__Actinobacteria	c__Actinobacteria	o__Actinomycetales	f__Propionibacteriaceae	g__Propionibacterium
431951	-2.04	3.9E-06	1.7E-03	p__Actinobacteria	c__Actinobacteria	o__Actinomycetales	f__Corynebacteriaceae	g__Corynebacterium
341093	-2.04	1.1E-08	2.7E-05	p__Actinobacteria	c__Actinobacteria	o__Actinomycetales	f__Actinomycetaceae	g__Actinomyces
38569	-2.07	3.1E-06	1.4E-03	p__Actinobacteria	c__Actinobacteria	o__Actinomycetales	f__Corynebacteriaceae	g__Corynebacterium
419772	-2.08	1.0E-07	1.3E-04	p__Actinobacteria	c__Actinobacteria	o__Actinomycetales	f__Corynebacteriaceae	g__Corynebacterium
424567	-2.08	7.6E-06	2.7E-03	p__Actinobacteria	c__Actinobacteria	o__Actinomycetales	f__Propionibacteriaceae	g__Propionibacterium
418160	-2.09	5.3E-09	1.6E-05	p__Actinobacteria	c__Actinobacteria	o__Actinomycetales	f__Corynebacteriaceae	g__Corynebacterium
455900	-2.13	3.4E-08	5.7E-05	p__Actinobacteria	c__Actinobacteria	o__Actinomycetales	f__Corynebacteriaceae	g__Corynebacterium
409973	-2.17	2.5E-05	6.3E-03	p__Actinobacteria	c__Actinobacteria	o__Actinomycetales	f__Propionibacteriaceae	g__Propionibacterium
439060	-2.18	3.9E-05	8.7E-03	p__Actinobacteria	c__Actinobacteria	o__Actinomycetales	f__Corynebacteriaceae	g__Corynebacterium
423144	-2.19	1.1E-05	3.4E-03	p__Actinobacteria	c__Actinobacteria	o__Actinomycetales	f__Corynebacteriaceae	g__Corynebacterium
356146	-2.19	4.0E-05	8.7E-03	p__Actinobacteria	c__Actinobacteria	o__Actinomycetales	f__Corynebacteriaceae	g__Corynebacterium
471342	-2.20	8.6E-06	2.9E-03	p__Actinobacteria	c__Actinobacteria	o__Actinomycetales	f__Corynebacteriaceae	g__Corynebacterium
418098	-2.21	1.1E-06	6.9E-04	p__Actinobacteria	c__Actinobacteria	o__Actinomycetales	f__Corynebacteriaceae	g__Corynebacterium
433751	-2.23	1.2E-07	1.5E-04	p__Actinobacteria	c__Actinobacteria	o__Actinomycetales	f__Corynebacteriaceae	g__Corynebacterium
432972	-2.26	1.6E-07	1.8E-04	p__Actinobacteria	c__Actinobacteria	o__Actinomycetales	f__Corynebacteriaceae	g__Corynebacterium
437733	-2.27	2.4E-06	1.2E-03	p__Actinobacteria	c__Actinobacteria	o__Actinomycetales	f__Corynebacteriaceae	g__Corynebacterium
434362	-2.28	7.3E-07	5.3E-04	p__Actinobacteria	c__Actinobacteria	o__Actinomycetales	f__Corynebacteriaceae	g__Corynebacterium
446618	-2.34	1.5E-08	3.4E-05	p__Actinobacteria	c__Actinobacteria	o__Actinomycetales	f__Corynebacteriaceae	g__Corynebacterium
408789	-2.37	5.5E-06	2.1E-03	p__Actinobacteria	c__Actinobacteria	o__Actinomycetales	f__Corynebacteriaceae	g__Corynebacterium
425647	-2.39	5.8E-06	2.2E-03	p__Actinobacteria	c__Actinobacteria	o__Actinomycetales	f__Brevibacteriaceae	g__Brevibacterium
358633	-2.41	2.7E-07	2.6E-04	p__Actinobacteria	c__Actinobacteria	o__Actinomycetales	f__Propionibacteriaceae	g__Propionibacterium
414133	-2.45	3.4E-05	7.8E-03	p__Actinobacteria	c__Actinobacteria	o__Actinomycetales	f__Corynebacteriaceae	g__Corynebacterium
454757	-2.47	2.0E-08	4.4E-05	p__Actinobacteria	c__Actinobacteria	o__Actinomycetales	f__Propionibacteriaceae	g__Propionibacterium
404744	-2.47	2.5E-06	1.3E-03	p__Actinobacteria	c__Actinobacteria	o__Actinomycetales	f__Corynebacteriaceae	g__Corynebacterium
416059	-2.47	2.4E-07	2.4E-04	p__Actinobacteria	c__Actinobacteria	o__Actinomycetales	f__Corynebacteriaceae	g__Corynebacterium
461321	-2.49	3.8E-08	6.2E-05	p__Actinobacteria	c__Actinobacteria	o__Actinomycetales	f__Corynebacteriaceae	g__Corynebacterium
462032	-2.49	9.4E-06	3.1E-03	p__Actinobacteria	c__Actinobacteria	o__Actinomycetales	f__Propionibacteriaceae	g__Propionibacterium
431287	-2.50	1.3E-06	8.1E-04	p__Actinobacteria	c__Actinobacteria	o__Actinomycetales	f__Corynebacteriaceae	g__Corynebacterium
444424	-2.53	1.5E-05	4.4E-03	p__Actinobacteria	c__Actinobacteria	o__Actinomycetales	f__Corynebacteriaceae	g__Corynebacterium
455972	-2.53	6.3E-07	4.6E-04	p__Actinobacteria	c__Actinobacteria	o__Actinomycetales	f__Corynebacteriaceae	g__Corynebacterium
453926	-2.56	3.4E-09	1.3E-05	p__Actinobacteria	c__Actinobacteria	o__Actinomycetales	f__Propionibacteriaceae	g__Propionibacterium
427900	-2.56	1.1E-08	2.7E-05	p__Actinobacteria	c__Actinobacteria	o__Actinomycetales	f__Propionibacteriaceae	g__Propionibacterium
427798	-2.60	2.3E-05	5.9E-03	p__Actinobacteria	c__Actinobacteria	o__Actinomycetales	f__Streptomycetaceae	g__Streptomyces
358230	-2.61	5.1E-09	1.6E-05	p__Actinobacteria	c__Actinobacteria	o__Actinomycetales	f__Corynebacteriaceae	g__Corynebacterium
425037	-2.64	2.1E-07	2.3E-04	p__Actinobacteria	c__Actinobacteria	o__Actinomycetales	f__Corynebacteriaceae	g__Corynebacterium
437521	-2.68	2.6E-07	2.5E-04	p__Actinobacteria	c__Actinobacteria	o__Actinomycetales	f__Corynebacteriaceae	g__Corynebacterium
426092	-2.71	7.1E-10	4.7E-06	p__Actinobacteria	c__Actinobacteria	o__Actinomycetales	f__Corynebacteriaceae	g__Corynebacterium
466881	-2.71	4.3E-05	9.2E-03	p__Actinobacteria	c__Actinobacteria	o__Actinomycetales	f__Propionibacteriaceae	g__Propionibacterium
446424	-2.73	3.7E-10	3.7E-06	p__Actinobacteria	c__Actinobacteria	o__Actinomycetales	f__Corynebacteriaceae	g__Corynebacterium
445124	-2.73	1.3E-09	6.5E-06	p__Actinobacteria	c__Actinobacteria	o__Actinomycetales	f__Corynebacteriaceae	g__Corynebacterium
427176	-2.78	2.7E-10	3.4E-06	p__Actinobacteria	c__Actinobacteria	o__Actinomycetales	f__Corynebacteriaceae	g__Corynebacterium
423665	-2.82	9.5E-09	2.6E-05	p__Actinobacteria	c__Actinobacteria	o__Actinomycetales	f__Corynebacteriaceae	g__Corynebacterium
437404	-2.83	4.1E-07	3.4E-04	p__Actinobacteria	c__Actinobacteria	o__Actinomycetales	f__Corynebacteriaceae	g__Corynebacterium
431002	-2.88	2.1E-05	5.6E-03	p__Actinobacteria	c__Actinobacteria	o__Actinomycetales	f__Corynebacteriaceae	g__Corynebacterium
426000	-2.91	8.4E-06	2.9E-03	p__Actinobacteria	c__Actinobacteria	o__Actinomycetales	f__Corynebacteriaceae	g__Corynebacterium
451691	-2.92	1.7E-05	4.9E-03	p__Proteobacteria	c__Betaproteobacteria	o__Neisseriales	f__Neisseriaceae	g__
445944	-2.92	8.5E-06	2.9E-03	p__Actinobacteria	c__Actinobacteria	o__Actinomycetales	f__Corynebacteriaceae	g__Corynebacterium
474905	-2.97	3.8E-05	8.7E-03	p__Actinobacteria	c__Actinobacteria	o__Actinomycetales	f__Micrococcaceae	g__Rothia
428433	-3.08	1.8E-06	1.0E-03	p__Actinobacteria	c__Actinobacteria	o__Bifidobacteriales	f__Bifidobacteriaceae	g__Gardnerella
428500	-3.14	9.7E-07	6.5E-04	p__Actinobacteria	c__Actinobacteria	o__Bifidobacteriales	f__Bifidobacteriaceae	g__Gardnerella
458116	-3.26	4.2E-06	1.8E-03	p__Actinobacteria	c__Actinobacteria	o__Actinomycetales	f__Propionibacteriaceae	g__Propionibacterium
444595	-3.37	3.6E-06	1.6E-03	p__Actinobacteria	c__Actinobacteria	o__Actinomycetales	f__Corynebacteriaceae	g__Corynebacterium

Table S7. Sample metadata.

ID	Name	Line*	Type	Date**	Time	T (ºC)	H(%)***	Influx_Quarterly	Inaguratior	Level	Wheel	Zone	Surroundings
AM01	Indios_verdes	3	Turnstile	02/05/16	14:23	30.4	27	10176457	1979	Underground	Rubber	North	Bus_station
AM02	Petroleo	5	Turnstile	29/04/16	13:17	28.3	30	499350	1982	Underground	Rubber	North	Office_building_area
AM03	Rosario	7	Turnstile	29/04/16	12:40	29.2	29	3220719	1983	Elevated	Rubber	North	Bus_station
AM04	Cuatro_Caminos	2	Turnstile	29/04/16	11:42	30.3	30	9523016	1984	Street_level	Rubber	West	Bus_station
AM05	Tacubaya	9	Turnstile	27/04/16	13:41	30.4	26	4190568	1988	Underground	Rubber	West	Bus_station
AM06	Observatorio	1	Turnstile	04/05/16	14:35	32.2	28	6489055	1972	Elevated	Rubber	West	Central_bus_station
AM07	Barranca	7	Turnstile	27/04/16	12:20	28.6	28	3347042	1985	Deep_Underground	Rubber	South	Office_building_area
AM08	Universidad	3	Turnstile	27/04/16	11:44	28.6	31	6353423	1983	Elevated	Rubber	South	Bus_station
AM09	Tasqueña	2	Turnstile	27/04/16	10:50	25.9	38	6856836	1970	Elevated	Rubber	South	Central_bus_station
AM10	Tlahuac	12	Turnstile	04/05/16	12:40	28.5	29	2997741	2012	Elevated	Steel	South	Bus_station
AM11	Buenavista	11	Turnstile	28/04/16	13:04	31.3	27	4866765	1999	Underground	Rubber	North	Central_bus_station
AM12	Constitucion	8	Turnstile	03/05/16	13:10	32.1	34	7934733	1994	Elevated	Rubber	East	Bus_station
AM13	La_Paz	10	Turnstile	03/05/16	12:02	29.1	37	2772477	1991	Elevated	Steel	East	Bus_station
AM14	Pantitlan	10	Turnstile	03/05/16	11:25	26.9	41	10023285	1991	Underground	Steel	East	Bus_station
AM15	Azteca	11	Turnstile	02/05/16	12:43	30.8	26	5604284	2000	Elevated	Rubber	East	Office_building_area
AM16	Carrera	6	Turnstile	02/05/16	13:46	30.2	26	2308196	1986	Underground	Rubber	North	Office_building_area
AM17	Deportivo	3	Turnstile	02/05/16	14:07	30.7	26	3152077	1979	Underground	Rubber	North	Park
AM18	Tacuba	2	Turnstile	29/04/16	12:05	29.6	29	3166159	1983	Underground	Rubber	North	Market
AM19	Muzquiz	11	Turnstile	02/05/16	12:30	30.8	28	2990086	2000	Elevated	Rubber	East	Office_building_area
AM20	San_Juan	8	Turnstile	28/04/16	13:26	30.6	27	2636193	1994	Underground	Rubber	Downtown	Office_building_area
AM21	Zocalo	2	Turnstile	28/04/16	11:42	29.1	31	6118795	1970	Underground	Rubber	Downtown	Plaza
AM22	Insurgentes	1	Turnstile	28/04/16	14:20	32.2	27	6489055	1969	Elevated	Rubber	Downtown	Office_building_area
AM23	Merced	1	Turnstile	29/04/16	14:30	31.4	30	4377638	1969	Street_level	Rubber	Downtown	Market
AM24	Chilpancingo	9	Turnstile	27/04/16	13:25	31.3	26	3893446	1988	Underground	Rubber	Downtown	Office_building_area
AM25	Train_L1	1	Handrail	28/04/16	14:25	32.5	27	61536150	1969	Underground	Rubber	Downtown	NA
AM26	Train_L2	2	Handrail	29/04/16	12:09	30.9	29	66232325	1970	Mixed	Rubber	Downtown	NA
AM27	Train_L3	3	Handrail	27/04/16	11:25	29.3	35	54258403	1983	Underground	Rubber	Downtown	NA
AM28	Train_L4	4	Handrail	02/05/16	13:35	32.7	26	7146862	1981	Elevated	Rubber	East	NA
AM29	Train_L5	5	Handrail	29/04/16	13:30	30.0	30	20137141	1983	Mixed	Rubber	North	NA
AM30	Train_L6	6	Handrail	29/04/16	12:50	28.8	30	11810259	1986	Underground	Rubber	North	NA
AM31	Train_L7	7	Handrail	27/04/16	12:35	27.7	30	24196452	1988	Deep_Underground	Rubber	West	NA
AM32	Train_L8	8	Handrail	03/05/16	13:29	31.6	34	32295124	1994	Mixed	Rubber	South	NA
AM33	Train_L9	9	Handrail	04/05/16	13:50	32.1	30	27481033	1988	Underground	Rubber	Downtown	NA
AM34	Train_L10	10	Handrail	03/05/16	11:38	27.9	41	23624923	1991	Street_level	Steel	East	NA
AM35	Train_L11	11	Handrail	02/05/16	12:50	31.3	26	39089567	2000	Mixed	Rubber	East	NA
AM36	Train_12	12	Handrail	04/05/16	12:05	27.6	37	23252166	2012	Mixed	Steel	South	NA
AM38	Train_L2_C	2	Handrail	28/04/16	12:23	31.1	26	66232325	1971	Mixed	Rubber	Downtown	NA
AM39	Train_L3_C	3	Handrail	27/04/16	11:55	29.3	32	54258403	1984	Underground	Rubber	Downtown	NA
AM40	Train_L4_C	4	Handrail	02/05/16	13:20	33.3	25	7146862	1981	Elevated	Rubber	East	NA
AM41	Train_L5_C	5	Handrail	29/04/16	13:29	30.2	30	20137141	1984	Mixed	Rubber	North	NA
AM42	Train_L6_C	6	Handrail	29/04/16	13:05	29.3	32	11810259	1986	Underground	Rubber	North	NA
AM43	Train_L7_C	7	Handrail	27/04/16	12:57	31.0	29	24196452	1988	Deep_Underground	Rubber	West	NA
AM44	Train_L8_C	8	Handrail	03/05/16	13:20	31.4	33	32295124	1994	Mixed	Rubber	South	NA
AM45	Train_L9_C	9	Handrail	04/05/16	14:06	32.3	30	27481033	1988	Underground	Rubber	Downtown	NA
AM46	Train_L10_C	10	Handrail	03/05/16	11:30	27.1	41	23624923	1991	Street_level	Steel	East	NA
AM47	Train_L10_C	11	Handrail	02/05/16	12:39	31.2	29	39089567	2000	Mixed	Rubber	East	NA
AM48	Train_12_C	12	Handrail	04/05/16	12:15	27.8	35	23252166	2012	Mixed	Steel	South	NA

* Line 10 is also called Line A. Line 12 is also called Line B.
** Date DD/MM/YY
*** Humidity (%)
